# Burden of chronic obstructive pulmonary disease and its attributable risk factors in 204 countries and territories, 1990–2021: results from the Global Burden of Disease Study 2021

**DOI:** 10.1136/bmjph-2024-002489

**Published:** 2026-01-13

**Authors:** Zhong Cao, Xunliang Tong, Liu He, Yuheng Luo, Ke Huang, Wei Li, Hongtao Niu, Qiushi Chen, Lirui Jiao, Sebastian Vollmer, Blake Thompson, Pascal Geldsetzer, Rifat Atun, Till Bärnighausen, Chen Wang, Ting Yang, Simiao Chen

**Affiliations:** 1 Heidelberg Institute of Global Health, Faculty of Medicine and University Hospital, Heidelberg University, Heidelberg, Germany; 2 Department of Pulmonary and Critical Care Medicine, National Center of Gerontology, Beijing Hospital, Institute of Geriatric Medicine, Chinese Academy of Medical Sciences, Beijing, China; 3 School of Population Medicine and Public Health, Chinese Academy of Medical Sciences & Peking Union Medical College, Beijing, China; 4 School of Health Policy and Management, Chinese Academy of Medical Sciences & Peking Union Medical College, Beijing, China; 5 Department of Pulmonary and Critical Care Medicine, China-Japan Friendship Hospital, Beijing, China; 6 Harold and Inge Marcus Department of Industrial and Manufacturing Engineering, The Pennsylvania State University, University Park, Illinois, USA; 7 Department of Health Policy and Management, The University of North Carolina at Chapel Hill Gillings School of Global Public Health, Chapel Hill, North Carolina, USA; 8 Department of Economics & Centre for Modern Indian Studies, University of Gottingen, Gottingen, Germany; 9 Surveillance and Health Equity Science, American Cancer Society, Atlanta, Georgia, USA; 10 Stanford University School of Medicine, Stanford, California, USA; 11 Department of Medicine, Stanford University School of Medicine, Stanford, California, USA; 12 Harvard T H Chan School of Public Health, Boston, Massachusetts, USA; 13 Global Health and Population, Harvard University T H Chan School of Public Health, Boston, Massachusetts, USA

**Keywords:** Public Health, Epidemiology, Epidemiologic Factors

## Abstract

**Background:**

Chronic obstructive pulmonary disease (COPD) remains a major global health challenge, contributing significantly to morbidity and mortality. This study aims to provide a comprehensive analysis of the burden of COPD by age, sex and Sociodemographic Index (SDI), in addition to its attributable risk factors across 204 countries and territories from 1990 to 2021.

**Methods:**

This study is a systematic analysis of data from the Global Burden of Disease (GBD) 2021 from 1990 to 2021 across 204 countries and territories. The study calculates age-standardised rates (ASRs) for prevalence, deaths and disability-adjusted life-years (DALYs) by adjusting rates to a global age distribution and computed estimated annual percentage changes (EAPC) for these ASRs and the relative COPD burden, while also exploring the relationships between the SDI and age-standardised DALYs per 1000 population via linear regression.

**Results:**

In 2021, there were an estimated 213.4 million prevalent COPD cases globally, with an ASR of 2512.9 per 100 000. From 1990 to 2021, the EAPC for ASRs in prevalence was −0.044%, while the EAPC for percentage in prevalence was 1.224%. COPD caused 3.7 million deaths, with an ASR of 45.2 per 100 000, and 79.8 million DALYs, with an ASR of 940.7 per 100 000. The leading risk factor for COPD globally was particulate matter pollution, where it accounted for 41.7% of the global DALYs. Appreciable geographical and demographic variations were observed, with North America exhibiting the greatest ASRs for prevalence and South Asia showing the greatest ASRs for death rates.

**Conclusions:**

The study highlights the persistent and evolving global burden of COPD, emphasising the significant impact of environmental factors such as particulate matter pollution. It underscores the need for targeted public health interventions and resource allocation, particularly in low-income and middle-income countries, to mitigate the growing COPD challenge. To enhance COPD management, the recommendations include implementing regional plans to mitigate particulate pollution, strengthening surveillance of air quality and health outcomes, developing integrated health strategies and supporting a global framework for air quality improvement.

**WHAT IS ALREADY KNOWN ON THIS TOPIC**

- Chronic obstructive pulmonary disease (COPD) is the third leading cause of mortality and disability worldwide, with key risk factors including smoking, air pollution and occupational exposure. Previous studies have highlighted a decline in COPD-related age-standardised mortality and disability-adjusted life-years (DALY) rates over the past decades.

**WHAT THIS STUDY ADDS**

- This study provides an updated, comprehensive assessment of the global COPD burden using Global Burden of Disease (GBD) 2021, revealing approximately 213.4 million cases worldwide and 3.7 million deaths. While the estimated annual percentage change (EAPC) for age-standardised rates (ASRs) in prevalence was slightly negative (–0.044%) between 1990 and 2021, the EAPC for the percentage of COPD relative to all-cause prevalence was notably positive (1.224%), highlighting a novel dimension introduced in this study that reveals COPD’s rising relative importance in the global disease landscape. Particulate matter pollution now surpasses smoking as the leading risk factor, contributing over 41.7% of the global DALYs. It reveals geographical variations, with North America showing the highest prevalence ASRs and South Asia the highest death ASRs.

**HOW THIS STUDY MIGHT AFFECT RESEARCH, PRACTICE OR POLICY**

- This study underscores the need for region-specific health policies to address the shifting COPD burden. Adapting interventions to specific regional risk factors, such as particulate matter pollution, can improve disease management and prevention.

## Introduction

Chronic obstructive pulmonary disease (COPD) continues to be a major cause of morbidity and mortality worldwide, presenting significant challenges in both diagnosis and management. Globally, COPD remains the third leading cause of death, contributing substantially to the Global Burden of Disease (GBD).^1^,^3^ The disease’s prevalence and impact vary significantly by region, influenced by factors such as smoking rates, air quality and access to healthcare.^4^

Understanding the global distribution and determinants of COPD is crucial for effective public health interventions and resource allocation. The GBD studies have consistently provided critical data that inform health policies and research priorities.^5^ However, the dynamic nature of global health risks, such as changes in smoking patterns, air pollution levels and demographic transitions, necessitates regular updates and comprehensive analyses of the evolving landscape.^6^

Despite the wealth of information provided by previous GBD studies, several critical knowledge gaps persist that limit our ability to develop effective, targeted interventions. First, while previous GBD analyses have documented a decreasing trend in age-standardised death rates from COPD, despite increasing absolute numbers due to population growth and ageing from 1990 to 2017,^7^ there is a lack of comprehensive analysis examining how COPD’s relative contribution to the total disease burden has evolved over time. Traditional approaches focus solely on changes in disease-specific rates but fail to capture whether COPD is becoming proportionally more or less important within the broader landscape of global health challenges. Second, although recent studies have recognised particulate matter pollution as a key emerging risk factor for COPD,^8^ few studies have quantitatively assessed the regional heterogeneity in the relative contribution of risk factors, especially the extent to which particulate pollution surpasses smoking in specific geographic settings. Third, the most recent comprehensive global analysis using GBD 2019 data does not capture the potential impacts of recent environmental policy changes, demographic transitions and the evolving global risk factor landscape that may have substantially altered COPD epidemiology patterns from 2019 to 2021. Fourth, there is limited understanding of how this relationship varies across the full spectrum of the Sociodemographic Index (SDI), particularly in countries undergoing rapid economic and environmental transitions. To address these knowledge gaps, our study leverages GBD 2021 data to provide an updated and comprehensive analysis of global COPD burden from 1990 to 2021. The GBD 2021 update significantly enhances our comprehension of COPD globally, providing a sophisticated analysis that reflects the latest epidemiological data and methodological advancements.^3^ Due to the data update in the GBD, we can analyse the shifting dynamics of COPD prevalence, deaths and disability-adjusted life-years (DALYs), highlighting the persistent growth in the disease burden, especially within low-income and middle-income countries (LMICs).

Our study addresses key research questions about the geographical and demographic variations in COPD burden and the extent to which socioeconomic factors influence these patterns. Furthermore, we have delved deep into the risk factors contributing to COPD, quantifying the impacts of smoking, air pollution and occupational exposures. These refined insights are crucial for public health authorities to tailor interventions more effectively and allocate resources more strategically. Our study not only underscores the ongoing global challenge posed by COPD but also highlights the critical need for targeted preventive strategies and enhanced clinical management to curb its impact. Through detailed graphical presentations in the subsequent sections of this article, we provide a visual exposition of these trends and factors, aiming to guide future policy and research directions to mitigate the COPD burden worldwide.

## Methodology

### Data sources

This analysis is based on data provided by the GBD 2021 update, which integrates a vast array of data sources including hospital and outpatient databases, civil registration systems and population-based epidemiological studies.^9^ These sources undergo meticulous vetting and standardisation processes to ensure consistency and comparability, furnishing robust inputs for the analysis. This extensive data integration allows for comprehensive global assessments of health trends and disease burdens.^10^

In line with the Global Initiative for Chronic Obstructive Lung Disease guidelines,^11^ COPD was defined by persistent respiratory symptoms and confirmed spirometric indications of airflow limitation that is not fully reversible. This uniform application of diagnostic criteria ensures that COPD cases are consistently classified across different regions, facilitating reliable comparisons and trend analyses.^12^

### Disease model

Transitioning from GBD 2019,^6^ the 2021 update incorporates refined data sources and advanced statistical modelling techniques, notably improving the precision of disease burden estimates. Key advancements include enhanced coverage of data-sparse populations and integration of the latest epidemiological data, enhancing the overall clarity of the global COPD landscape.^13^ The core of the GBD’s analytical approach, the DisMod-MR 2.1 Bayesian meta-regression tool, has been updated to better model non-linear relationships and demographic and risk factor interactions, allowing for more nuanced disease impact assessments.^14 15^

A novel addition in GBD 2021 is the evaluation of COVID-19’s impact on chronic diseases such as COPD. The present study shows the impact of the pandemic on disease progression and management, incorporating disruptions to healthcare services and changes in environmental risk exposures.^3^

### Risk factor analysis

The present study also shows the results of the risk factor analysis, quantifying the contributions of tobacco smoke, air pollution and occupational exposures to the COPD burden. This analysis employs updated relative risks and exposure data to assess the impact of these risk factors within diverse environmental and socioeconomic contexts, aiding in the design of targeted public health interventions.^7^

### Statistical analysis

Our analysis of the burden of COPD involves several key steps, each carefully designed to ensure robust comparisons across regions and over time. Age-standardised rates (ASRs) for prevalence, deaths and DALYs are calculated by adjusting the crude rates for each age group to a standard global age distribution. This standardisation enables a meaningful comparison of COPD burden across different populations, irrespective of their age structures.

We present estimated annual percentage changes (EAPC) for ASRs in prevalence, deaths and DALYs over specified time periods (ie, 1990–2021, 2019–2021). Additionally, we also present the EAPC for the percentage of the burden attributable to COPD within all-cause prevalence, deaths and DALYs over specified time periods (ie, 1990–2021, 2019–2021). This annualised rate of change provides a clear measure of the year-over-year variation in the proportion of COPD as a contributor to all-cause prevalence, deaths and DALYs. This EAPC for percentage indicates the shifts in COPD burden as a part of total disease burden, enabling researchers to track trends and inform targeted public health strategies based on the evolving impact of the disease. All estimates were reported with 95% uncertainty intervals (UIs), defined as the 2.5th and 97.5th values of the ordered 1000 draws from the posterior distributions generated through the GBD modelling framework. The EAPC, however, was presented with 95% CIs.

We employ a linear regression model and a Locally Weighted Scatterplot Smoothing (LOWESS) model to examine the relationship between the SDI and the age-standardised DALYs per 1000 population. Model performance was assessed using the coefficient of determination (R²), root mean square error (RMSE) and mean absolute error (MAE). All statistical analyses and graph plotting were conducted using Python software (V.3.7.10).

### Patient and public involvement

Given that this study used publicly available aggregate data, patient involvement was not incorporated in the formulation of the research question, selection of outcome measures or in the design and execution of the study.

## Results

Table 1 details the global prevalence, deaths and DALYs for COPD in 2021. In 2021, there were 213.4 million (95%UI: 194.9 to 234.0) prevalent cases of COPD globally, with an ASR of 2512.9 (2293.9 to 2748.5) per 100 000. The EAPC for ASRs in prevalence was −0.044% (–0.076% to –0.012%) from 1990 to 2021, and −0.155% (–0.370% to 0.061%) from 2019 to 2021. The EAPC for percentage in prevalence was 1.224% (1.211% to 1.238%) from 1990 to 2021, with a similar trend (ie, 1.204%, 0.931% to 1.478%) observed from 2019 to 2021. There were 3.7 million (3.3 to 4.1) deaths attributed to COPD, with an ASR of 45.2 (40.6 to 49.7) per 100 000. The EAPC for ASRs in deaths was −1.745% (–1.836% to –1.655%) from 1990 to 2021, while the EAPC for percentage in deaths remained nearly unchanged between 1990 and 2021 (0.272%, 0.174% to 0.370%), indicating a stable proportion of all-cause deaths attributable to COPD over this period. Notably, during 2019–2021, the EAPC for percentage in deaths showed a marked reduction of 6.839% (8.499% to 5.149%). COPD caused an estimated 79.8 million (74.0 to 86.0) DALYs globally, with an ASR of 940.7 (871.5 to 1014.6) per 100 000. The EAPC for ASRs in DALYs was −1.712% (–1.789% to –1.635%), and the EAPC for percentage in DALYs was 0.834% (0.753% to 0.916%) during 1990 to 2021, which was −3.460% (–4.246% to –2.668%) from 2019 to 2021.

**Table 1:** COPD prevalence, deaths and DALYs in 2021, with age-standardised rates (ASRs) per 100 000 in 2021, as well as estimated annual percentage change (EAPC) for ASRs and for the percentage of COPD burden relative to all-cause prevalence, deaths and DALYs from 1990 to 2021 and from 2019 to 2021, by GBD regions

Measure	Location	No, in thousands, in 2021 (95% UI)	ASRs, per 100,000, in 2021 (95% UI)	EAPC for ASRs from 1990 to 2021 (95% CI)	EAPC for ASRs from 2019 to 2021 (95% CI)	EAPC for percentage from 1990 to 2021 (95% CI)	EAPC for percentage from 2019 to 2021 (95% CI)
Prevalence	Global	213 387.4 (194 868.1 to 233 975.9)	2512.9 (2293.9 to 2748.5)	−0.044 (−0.076 to −0.012)	−0.155 (−0.370 to 0.061)	1.224 (1.211 to 1.238)	1.204 (0.931 to 1.478)
	East Asia & Pacific - WB	73 565.0 (65 382.6 to 82 590.8)	2295.6 (2050.1 to 2555.2)	−0.276 (−0.309 to −0.244)	−0.212 (−0.634 to 0.212)	1.842 (1.826 to 1.858)	2.100 (1.724 to 2.479)
	Europe & Central Asia - WB	40 053.0 (36 261.6 to 43 780.7)	2505.5 (2293.8 to 2741.6)	−0.090 (−0.111 to −0.070)	−0.252 (−0.401 to −0.103)	1.099 (1.081 to 1.117)	0.740 (0.531 to 0.949)
	Latin America & Caribbean - WB	15 148.2 (13 647.2 to 16 803.1)	2171.0 (1955.5 to 2399.9)	0.147 (0.092 to 0.202)	−0.024 (−0.273 to 0.225)	1.972 (1.955 to 1.989)	1.555 (1.467 to 1.643)
	Middle East & North Africa - WB	8028.3 (7153.6 to 8972.2)	2322.6 (2066.5 to 2599.4)	0.665 (0.653 to 0.677)	0.506 (0.026 to 0.987)	1.836 (1.789 to 1.882)	1.909 (1.338 to 2.483)
	North America	21 494.7 (20 380.5 to 22 538.3)	3298.6 (3131.8 to 3451.6)	0.266 (0.145 to 0.387)	−0.424 (−0.727 to −0.121)	1.336 (1.271 to 1.402)	0.981 (0.655 to 1.307)
	South Asia - WB	44 564.4 (40 330.9 to 48 614.4)	2996.5 (2709.1 to 3273.6)	0.129 (0.099 to 0.160)	−0.189 (−0.292 to −0.086)	1.485 (1.442 to 1.528)	1.288 (1.081 to 1.496)
	Sub-Saharan Africa - WB	10 279.6 (9174.2 to 11 413.7)	1814.1 (1603.3 to 2023.2)	0.171 (0.148 to 0.193)	0.831 (0.725 to 0.937)	0.175 (0.097 to 0.253)	1.068 (0.898 to 1.238)
	Low SDI	12 666.8 (11 450.8 to 13 814.5)	2337.5 (2119.4 to 2567.8)	0.206 (0.186 to 0.227)	0.399 (0.349 to 0.450)	0.292 (0.232 to 0.353)	0.723 (0.557 to 0.888)
	Low-middle SDI	38 692.6 (35 061.9 to 42 291.5)	2726.8 (2478.2 to 2979.0)	0.136 (0.113 to 0.160)	0.028 (−0.009 to 0.066)	1.281 (1.248 to 1.314)	1.189 (1.006 to 1.372)
	Middle SDI	63 927.6 (57 320.7 to 71 316.4)	2463.2 (2208.8 to 2736.0)	−0.007 (−0.031 to 0.017)	−0.111 (−0.374 to 0.153)	1.863 (1.836 to 1.890)	1.937 (1.658 to 2.218)
	High-middle SDI	45 671.5 (40 973.8 to 50 846.2)	2385.3 (2149.8 to 2648.7)	−0.216 (−0.247 to −0.186)	−0.060 (−0.195 to 0.075)	1.353 (1.344 to 1.362)	1.722 (1.595 to 1.849)
	High SDI	52 256.4 (48 386.8 to 55 979.6)	2527.7 (2364.4 to 2701.8)	−0.041 (−0.097 to 0.015)	−0.546 (−0.984 to −0.106)	1.302 (1.261 to 1.343)	0.851 (0.403 to 1.300)
Deaths	Global	3719.9 (3347.9 to 4084.2)	45.2 (40.6 to 49.7)	−1.745 (−1.836 to −1.655)	−0.952 (−1.223 to −0.680)	0.272 (0.174 to 0.370)	−6.839 (−8.499 to −5.149)
	East Asia & Pacific - WB	1602.6 (1354.3 to 1862.2)	52.3 (44.0 to 60.7)	−3.694 (−3.873 to −3.514)	−0.479 (−0.839 to −0.118)	−1.214 (−1.362 to −1.066)	−1.382 (−2.741 to −0.004)
	Europe & Central Asia - WB	330.6 (294.9 to 351.4)	18.0 (16.2 to 19.0)	−1.794 (−1.922 to −1.666)	−1.679 (−2.149 to −1.208)	−0.139 (−0.320 to 0.042)	−9.011 (−11.678 to −6.263)
	Latin America & Caribbean - WB	165.5 (149.2 to 176.4)	24.4 (22.0 to 26.0)	−1.242 (−1.418 to −1.065)	−1.808 (−1.904 to −1.712)	0.649 (0.264 to 1.036)	−14.036 (−19.003 to −8.764)
	Middle East & North Africa - WB	52.6 (47.0 to 59.3)	19.9 (17.6 to 22.4)	−0.990 (−1.061 to −0.919)	−1.280 (−1.653 to −0.907)	0.842 (0.652 to 1.032)	−11.483 (−13.887 to −9.011)
	North America	213.2 (185.7 to 226.2)	29.9 (26.2 to 31.6)	0.348 (0.150 to 0.546)	−0.209 (−1.410 to 1.007)	1.365 (1.093 to 1.638)	−7.219 (−14.323 to 0.474)
	South Asia - WB	1244.2 (1119.5 to 1395.6)	100.3 (89.4 to 112.8)	−0.220 (−0.359 to −0.080)	−2.485 (−2.711 to −2.258)	2.379 (2.172 to 2.585)	−9.543 (−11.395 to −7.652)
	Sub-Saharan Africa - WB	108.9 (94.6 to 126.4)	28.4 (24.7 to 32.9)	−0.871 (−0.919 to −0.822)	−0.803 (−0.942 to −0.664)	1.308 (1.065 to 1.552)	−7.357 (−7.898 to −6.812)
	Low SDI	266.0 (237.4 to 301.0)	70.7 (63.3 to 79.8)	−0.109 (−0.272 to 0.055)	−2.158 (−2.627 to −1.687)	2.270 (2.022 to 2.518)	−8.158 (−8.504 to −7.810)
	Low-middle SDI	999.8 (898.2 to 1104.5)	84.8 (75.8 to 93.8)	−0.124 (−0.228 to −0.020)	−2.052 (−2.325 to −1.778)	2.192 (1.999 to 2.385)	−9.461 (−11.476 to −7.401)
	Middle SDI	1293.7 (1122.2 to 1476.7)	57.5 (49.6 to 65.4)	−2.849 (−2.976 to −2.722)	−0.552 (−1.047 to −0.055)	−0.682 (−0.807 to −0.558)	−6.586 (−7.491 to −5.672)
	High-middle SDI	689.4 (592.0 to 781.7)	35.9 (30.8 to 40.7)	−3.150 (−3.387 to −2.912)	−0.188 (−0.336 to −0.040)	−1.284 (−1.399 to −1.169)	−5.402 (−6.617 to −4.172)
	High SDI	469.2 (410.4 to 501.1)	19.4 (17.3 to 20.7)	−0.969 (−1.037 to −0.901)	−0.862 (−1.963 to 0.251)	0.650 (0.519 to 0.781)	−4.666 (−8.621 to −0.540)
DALYs	Global	79 779.7 (74 026.4 to 86 011.4)	940.7 (871.5 to 1014.6)	−1.712 (−1.789 to −1.635)	−0.941 (−1.260 to −0.622)	0.834 (0.753 to 0.916)	−3.460 (−4.246 to −2.668)
	East Asia & Pacific - WB	31 045.1 (27 188.7 to 35 401.9)	973.5 (855.8 to 1108.0)	−3.552 (−3.704 to −3.400)	−0.559 (−0.859 to −0.257)	−0.403 (−0.538 to −0.268)	−0.249 (−1.178 to 0.688)
	Europe & Central Asia - WB	6989.1 (6473.3 to 7399.5)	417.7 (390.4 to 442.6)	−1.667 (−1.794 to −1.538)	−1.308 (−1.648 to −0.966)	0.069 (−0.089 to 0.227)	−6.227 (−7.859 to −4.565)
	Latin America & Caribbean - WB	3401.9 (3156.5 to 3613.0)	490.5 (454.8 to 520.9)	−1.217 (−1.381 to −1.052)	−0.967 (−1.081 to −0.853)	1.277 (0.994 to 1.560)	−7.793 (−10.427 to −5.082)
	Middle East & North Africa - WB	1576.2 (1429.4 to 1750.9)	485.2 (441.9 to 538.1)	−0.820 (−0.882 to −0.758)	−0.816 (−1.106 to −0.524)	1.706 (1.567 to 1.846)	−4.869 (−5.669 to −4.063)
	North America	4946.1 (4577.3 to 5221.6)	735.9 (685.7 to 776.4)	0.128 (−0.025 to 0.282)	−0.349 (−1.109 to 0.417)	1.098 (0.903 to 1.293)	−4.499 (−9.329 to 0.589)
	South Asia - WB	28 344.7 (25 714.4 to 31 289.9)	2021.9 (1837.9 to 2237.8)	−0.469 (−0.543 to −0.394)	−2.181 (−2.508 to −1.854)	2.915 (2.799 to 3.031)	−4.856 (−5.644 to −4.060)
	Sub-Saharan Africa - WB	3419.4 (3010.4 to 3866.1)	696.2 (613.5 to 786.5)	−0.713 (−0.749 to −0.676)	−0.345 (−0.351 to −0.339)	1.874 (1.630 to 2.118)	−1.554 (−1.574 to −1.534)
	Low SDI	6693.6 (6035.1 to 7482.9)	1457.9 (1318.8 to 1617.0)	−0.380 (−0.464 to −0.295)	−1.712 (−1.855 to −1.568)	2.317 (2.118 to 2.516)	−2.828 (−3.007 to −2.648)
	Low-middle SDI	22 727.3 (20 885.6 to 24 793.6)	1707.9 (1558.9 to 1865.1)	−0.379 (−0.439 to −0.319)	−1.934 (−2.262 to −1.604)	2.718 (2.601 to 2.834)	−4.726 (−5.602 to −3.841)
	Middle SDI	26 679.2 (23 958.7 to 29 735.6)	1076.7 (963.6 to 1201.2)	−2.827 (−2.940 to −2.715)	−0.637 (−1.027 to −0.245)	0.050 (−0.059 to 0.159)	−3.406 (−3.811 to −3.000)
	High-middle SDI	13 439.4 (12 068.5 to 15 035.5)	691.1 (621.8 to 772.7)	−3.061 (–3.253 to –2.869)	−0.281 (–0.425 to –0.138)	−0.819 (–0.934 to –0.704)	−3.163 (–3.658 to –2.665)
	High SDI	10 197.0 (9399.3 to 10 811.5)	471.2 (437.4 to 498.8)	−0.757 (–0.800 to –0.714)	−0.800 (–1.609 to 0.015)	0.749 (0.660 to 0.838)	−3.016 (–5.832 to –0.116)

COPD, chronic obstructive pulmonary disease; DALYs, disability-adjusted life-years; GBD, Global Burden of Disease; SDI, Sociodemographic Index; WB, regions as defined by the World Bank.

The North America region exhibited the greatest ASRs of prevalence at 3298.6 per 100 000. Europe and Central Asia recorded the lowest ASRs of death per 100 000 at 18.0 and the lowest ASRs of DALYs per 100 000 at 417.7. East Asia and the Pacific reported the greatest number of prevalence cases, totalling 73.6 million and the greatest number of deaths, totalling 1.6 million. South Asia had the greatest ASRs of death per 100 000 at 100.3 and the greatest EAPC for percentage in DALYs at 2.379% from 1990 to 2021. Latin America and the Caribbean experienced the greatest EAPC for percentage in prevalence at 1.972% from 1990 to 2021. Sub-Saharan Africa had the lowest EAPC for percentage in prevalence at 0.175% from 1990 to 2021. Meanwhile, the Middle East and North Africa reported the lowest numbers of COPD prevalence, deaths and DALYs, with totals of 8.0 million, 52.6 thousand and 1.6 million, respectively.

High SDI groups had the lowest ASRs for COPD deaths (19.4) and DALYs (471.2) per 100 000. Middle SDI groups had the greatest increases in the EAPC (1.863%) for percentage in prevalence from 1990 to 2021, and they also recorded the greatest number of deaths, totalling 1.294 million. Low-middle SDI groups display the greatest ASRs for prevalence (2726.8), deaths (84.8) and DALYs (1707.9) per 100 000. These groups also show the largest increases in EAPC (2.718 %) for percentage in DALYs from 1990 to 2021. Meanwhile, Low SDI groups display the lowest ASRs for prevalence (2337.5).

**Figure 1:**
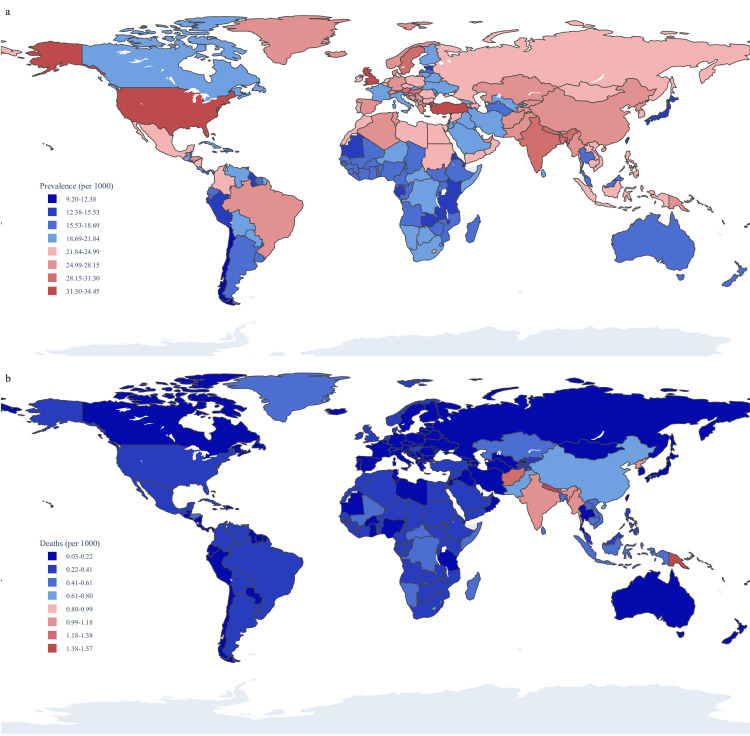
(**a**) Age-standardised prevalence of chronic obstructive pulmonary disease per 1000 population in 2021, by country. (**b**) Age-standardised death of chronic obstructive pulmonary disease per 1000 population in 2021, by country.

In 2021, the national age-standardised point prevalence of COPD varied significantly across countries. The greatest estimated ASRs per 100 000 were in the USA (3445.3), UK (3270.3) and Turkey (2146.7), whereas Singapore (922.6), Cabo Verde (1001.3) and Chile (1213.7) had the lowest (figure 1a and online supplemental table S1). There was also substantial variation in ASRs for COPD deaths in 2021, with the greatest rates per 100 000 in Papua New Guinea (156.8), Nepal (146.1), and India (108.3), and the lowest in Kuwait (2.7), Japan (5.8), and Montenegro (5.9) (figure 1b and online supplemental table S2). Additionally, the age-standardised DALY rates for COPD in 2021 mirrored the top three rankings of death rates per 100 000, with the greatest in Papua New Guinea (3004.4), Nepal (2836.0), and India (2171.2), and the lowest in Singapore (146.5), Japan (155.8), and Kuwait (160.5) (online supplemental table S3). The EAPC for ASRs varied significantly across countries. The greatest EAPC for ASRs in prevalence was observed in Saudi Arabia (1.076%), while the lowest was recorded in Singapore (−1.631%) (online supplemental table S1). For deaths, the greatest EAPC for ASRs was found in Georgia (2.841%), and the lowest in Belarus (−7.327%) (online supplemental table S2). In terms of DALYs, the highest EAPC for ASRs was reported in Saint Vincent and the Georgia (2.002%), with the lowest again observed in Belarus (−5.737%) (online supplemental table S3).

The EAPC for percentage in prevalence, deaths and DALYs from 1990 to 2021 varied significantly among countries. The UUSA (3.751%), Republic of Korea (3.632%) and Albania (3.371%) experienced the greatest EAPC for percentage in prevalence, while Afghanistan (−1.733%), Equatorial Guinea (−0.778%) and Chad (−0.557%) had the lowest (online supplemental table S4). During the same period, Timor-Leste (4.104%), Norway (3.925%) and Bhutan (3.703%) recorded the greatest EAPC for percentage in deaths. Conversely, the lowest EAPC for percentage in deaths was observed in Belarus (−6.553%), Ukraine (−5.751%) and Uzbekistan (−3.620%) (online supplemental table S5). Additionally, Timor-Leste (5.021%), Bhutan (4.225%) and Nepal (4.119%) had the greatest EAPC for percentage in DALYs. The lowest EAPC for percentage in DALYs during this period occurred in Belarus (−4.720%), Ukraine (−4.674%) and Kyrgyzstan (−2.978%) (online supplemental table S6).

### Age and sex disparities

**Figure 2:**
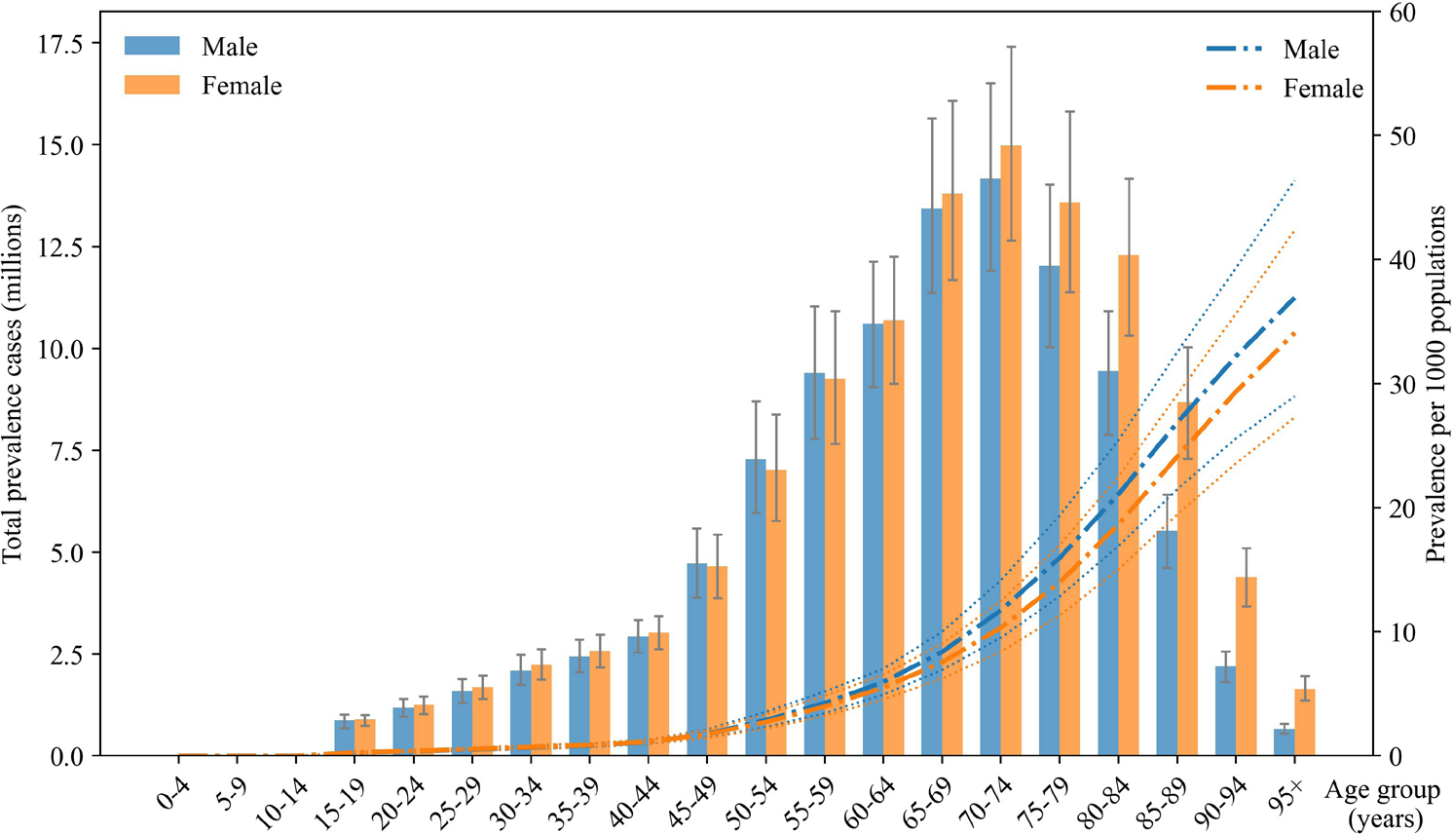
Global prevalent cases (in thousands) and global prevalence per 1000 population of COPD, by age and sex in 2021. The lines on the bar graphs and the dotted lines around the line graphs are 95% uncertainty intervals for prevalent cases and prevalence rates, respectively. COPD, chronic obstructive pulmonary disease.

Figure 2 illustrates the age and sex disparities in both the number of prevalent cases and prevalence rate per 1000 individuals of COPD across diverse age and sex groups. In the younger age groups (0–44 years), the prevalent cases is relatively low, with a slight male predominance. Notably, prevalent cases escalates significantly for both sexes from ages 45 to 74, peaking within the 70–74 age bracket, where males exhibit a marginally fewer prevalent cases than females. The most pronounced disparity occurs in the 85–89 age group, with 3.2 million cases. Beyond the age of 90, prevalent cases declines for both sexes and the disparity between them diminishes.

The prevalence rate consistently increases across both sexes. From ages 0 to 49, the difference in prevalence rates between men and women remains minimal. However, after age 50, the prevalence rate for men not only surpasses that for women but also widens progressively, ranging from 0.16 to 2.8 per 1000. These trends highlight critical periods where COPD is particularly prevalent in older age groups and underscore the variable impact on males and females, with females generally experiencing higher case numbers while males exhibit higher rates.

## Impact of socioeconomic factors

**Figure 3:**
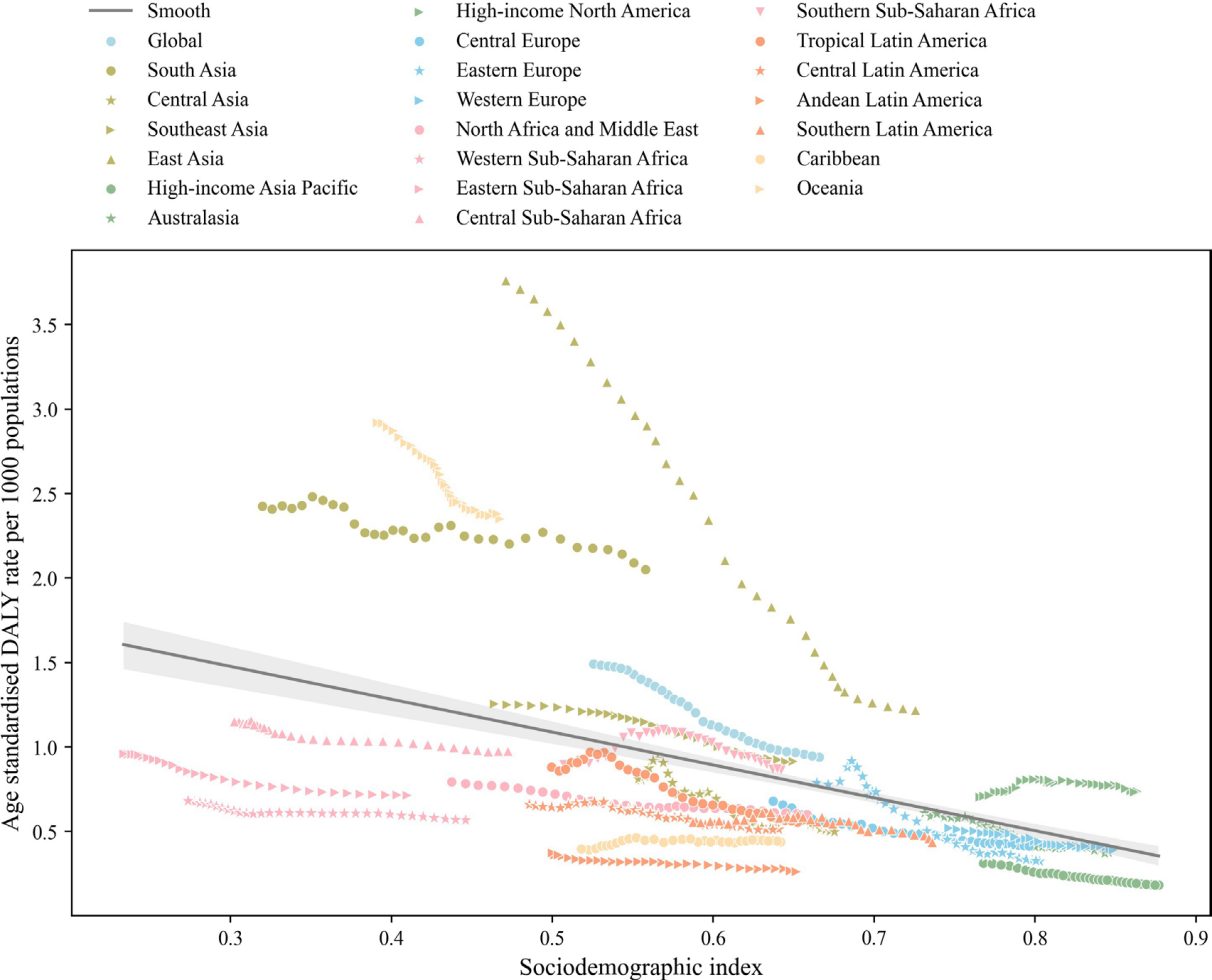
Comparative distribution of age-standardised DALY rates for COPD across 21 global regions by sociodemographic index, with each point representing annual data from 1990 to 2021. The black line illustrates the regression result, whereas the grey shaded area denotes the 95% CI. COPD, chronic obstructive pulmonary disease; DALY, disability-adjusted life-year.

Figure 3 illustrates the inverse relationship between the SDI and the age-standardised DALY rate of COPD from 1990 to 2021. As the SDI increases, the age-standardised DALY rate generally decreases. The linear regression revealed a statistically significant negative association between SDI and age-standardised DALYs (β=2.0603, 95% CI 1.884 to 2.237, p<0.001), with an R² of 0.200, RMSE of 0.605 and MAE of 0.427, indicating a moderate but better-fitting model compared with the LOWESS model. Notably, regions such as South Asia, East Asia, Oceania and High-income North America exhibited higher than expected DALY rates, given their SDI, throughout the period studied. Conversely, regions including North Africa and the Middle East, various sub-Saharan African regions (Western, Eastern, Central), Central Latin America, Andean Latin America, Southern Latin America, the Caribbean and High-income Asia Pacific experienced lower than anticipated burdens. Meanwhile, Global, Southeast Asia, Australasia, Central Europe, Eastern Europe, Western Europe, Southern Sub-Saharan Africa and Tropical Latin America displayed DALY rates consistent with their respective SDI.

### Risk factors

**Figure 4:**
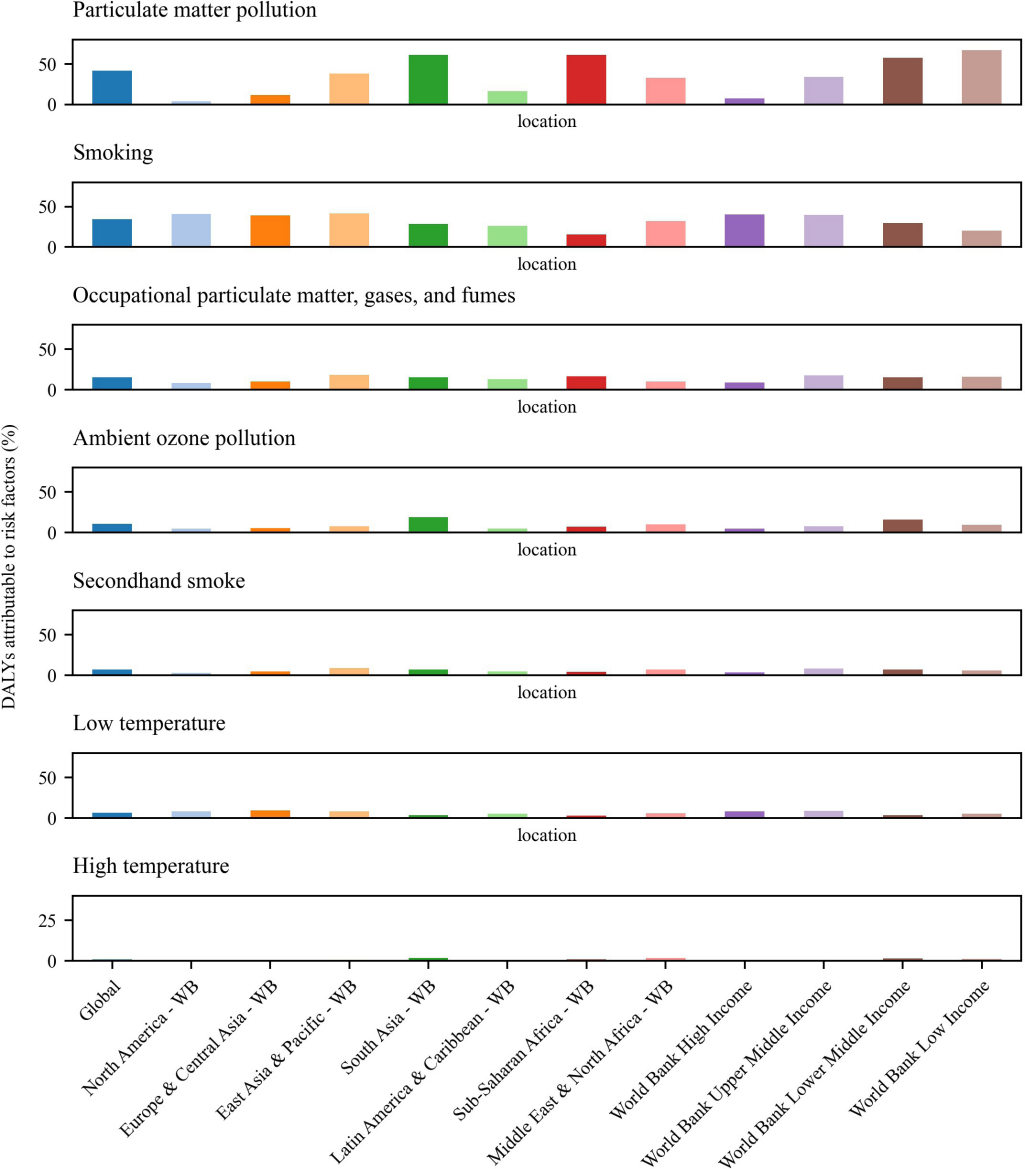
Percentage of DALYs attributable to each risk factor for COPD. COPD, chronic obstructive pulmonary disease; DALY, disability-adjusted life-year. Region names ending with “-WB” indicate that the regions are defined according to the World Bank classification.

Figure 4 highlights the proportion of DALYs attributable to various risk factors for COPD. Particulate matter pollution (41.4%) becomes the leading risk factor globally, followed by smoking (34.6%), occupational exposures (15.7%), ambient ozone pollution (10.9%), secondhand smoke (7.1%), low temperature (6.3%) and high temperature (0.9%), though this varied by region. Sub-Saharan Africa and South Asia reported the greatest DALYs percentages for COPD due to particulate matter pollution, 61.0% and 60.9%, respectively. Smoking as a risk factor had the largest impact in East Asia and Pacific, North America, as well as Europe & Central Asia, with the proportion of DALYs attributable to smoking around 41.2%, 41.1% and 39.1%, respectively. Occupational exposure to particulates, gases and fumes was most notable in East Asia and the Pacific (18.4%), Sub-Saharan Africa (16.9%) and South Asia (15.6%). Ambient ozone pollution had a considerable impact in South Asia (18.8%) as well as Middle East and North Africa (10.3%).

The health impacts of various risk factors associated with COPD differ across World Bank income groups. Low-income regions had the most severe effects, with particulate matter pollution contributing to 66.7% of COPD DALYs. In contrast, the impact of smoking in these low-income regions was less severe across different income groups, accounting for 20.0% of DALYs. Middle-income regions, both lower and upper, show that a substantial proportion of COPD DALY burden was attributable to both smoking (ranging from 29.9% to 39.9%) and particulate matter pollution (33.8% to 57.4%). High-income regions had the greatest proportion of DALYs attributable to smoking (40.0%), as opposed to 7.6% from particulate matter pollution. Occupational exposures and ambient ozone pollution also exhibit varied impacts, with middle-income groups facing greater challenges compared with other groups. Temperature extremes, particularly low temperatures, consistently had moderate effects across all income levels, while high temperatures had a minimal impact. Secondhand smoke generally posed minimal risk, causing an estimated 3.7% of COPD DALYs in high-income groups, as compared with 5.7% in low-income groups and 8.5% in upper-middle-income groups.

## Discussion

This study underscores the persistent global health challenge posed by COPD. First, it illuminates the escalating challenge of COPD, presenting data on over 213.4 million cases, 3.7 million deaths and approximately 79.8 million DALYs globally. Second, it emphasises significant disparities in disease burden across different geographic, demographic and socioeconomic contexts, highlighting the particularly heavy impact on low SDI regions such as Sub-Saharan Africa, South Asia and Oceania. Third, the study offers a detailed analysis of regional risk factors, enhancing the understanding of COPD’s drivers. These insights are crucial for shaping global health initiatives, directing research efforts and informing policy strategies aimed at mitigating the worldwide COPD burden, thereby establishing the study as a critical resource for health policymakers and international health organisations.

Compared with other studies examining trends in COPD burden, our study highlights the complex epidemiological landscape in which COPD has persisted over the last three decades. Saeid Safiri *et al* reported a notable decrease in age-standardised incidence rates, mortality rates and DALY rates from 1990 to 2019,^16^ which did not correspond to changes in the relative burden of COPD compared with other diseases. In contrast, Boers *et al* forecast a 23% increase in the number of global COPD cases among those aged 25 years and older by 2050, suggesting an impending rise in COPD’s public health impact due to demographic shifts.^17^ This study aligns with previous findings, revealing a decline in age-standardised incidence, mortality and DALY rates over the past three decades. However, it also highlights the paradoxical increase in absolute numbers—primarily reflecting population ageing rather than increased age-specific risk—emphasising the growing global challenge posed by COPD. To further explore COPD’s burden relative to other diseases, the EAPC for percentage in prevalence, mortality and DALYs was calculated in this study. We found that the percentage in all-cause prevalence increased at an annual rate of 1.2%, and the percentage in all-cause DALYs at a rate of 0.8%, while the percentage in all-cause deaths remained steady from 1990 to 2021. Interestingly, the last 2 years showed an increase in the percentage in all-cause prevalence, even as the percentage in all-cause deaths and DALYs has decreased. This indicates that while the overall impact of COPD is becoming more prevalent relative to other diseases, its relative mortality and DALY impacts are diminishing, suggesting improvements in clinical management but persistent challenges in prevention.

Regionally, the prevalence of COPD as a proportion of all diseases varied during the overall study period (1990–2021) and in recent short-term (2019–2021) trends. Low SDI countries had a lower EAPC for percentage in prevalence of 0.3%, while middle SDI regions had a higher EAPC for percentage in prevalence of 1.9%. Notably, East Asia and the Pacific have exhibited a positive EAPC for percentage in prevalence above the long-term trend in the last 2 years, pointing to a growing relative impact of COPD in these regions. Additionally, the Sub-Saharan Africa region has seen a substantial short-term increase compared with long-term changes, potentially due to improved monitoring and detection of COPD. This analysis underscores the varying regional dynamics of COPD burden and highlights the critical need for tailored public health strategies to address the unique challenges faced by different regions.

Regional differences in COPD burden are driven by disparities in healthcare systems, air pollution control and tobacco regulation. In high-income countries, patients often benefit from access to both primary and specialist care and comprehensive COPD management guidelines; however, healthcare delivery is frequently fragmented, with poor coordination among providers and misaligned reimbursement mechanisms that undermine continuity and efficiency.^18 19^ In contrast, LMICs face limited primary care capacity, shortages of trained clinicians and delayed diagnosis, further compounded by underinvestment in chronic disease management.^5 20^ Air pollution control efforts also vary widely. Despite global frameworks, many low-income countries lack enforceable regulations and institutional capacity, and air quality remains a low priority on national health agendas.^21^ Tobacco control shows similar disparities: while some high-income settings implement integrated cessation programme such as Canada’s Ottawa Model,^22^ many LMICs, despite high smoking prevalence, lack evidence-based cessation protocols, face weak implementation infrastructures and have limited access to pharmacological or behavioural interventions.^23^ These systemic limitations likely contribute to the divergent epidemiological trajectories of COPD across regions and highlight the need for policy responses tailored to local capacities and constraints.

Sex differences in COPD are influenced by various biological, behavioural and diagnostic factors. Biologically, females have smaller airways relative to their lung size, which may make them more susceptible to the effects of pollutants and cigarette smoke, potentially leading to earlier onset and more severe progression of COPD compared with men.^24 25^ Behaviourally, while historically more male individuals smoked, which aligned with higher COPD rates, recent shifts in smoking habits have seen increases among females, potentially modifying future trends in COPD prevalence between sexes.^26^ Moreover, occupational exposures differ traditionally by sex, with men more likely to work in high-risk industries; however, as these roles evolve, exposure profiles for females are also changing.^27^ Diagnostically, there has been a bias toward viewing COPD as a disease primarily affecting male smokers, possibly leading to underdiagnosis in females who may present with different symptoms or less typical risk profiles.^28^ Females tend to use more healthcare services, which might lead to higher detection rates, yet they often experience more severe symptoms and worse quality of life outcomes.^29 30^ This complex interplay of factors highlights the need for sex-specific research, public health interventions and clinical approaches to more effectively manage and treat COPD across different populations.

Smoking has been widely recognised as the primary risk factor for COPD. Numerous studies have consistently demonstrated that tobacco smoke is a major contributor to the development and progression of COPD, leading to significant global health burdens.^31 32^ Additionally, occupational exposure to dusts and chemicals has also been highlighted as critical risk factors, particularly in industrial settings.^33^ Ambient air pollution, including particulate matter and ozone, has been increasingly recognised for its role in exacerbating respiratory diseases, including COPD.^34^,^36^ Our research presents recent shifts in the landscape of COPD risk factors. While smoking remains a significant risk factor, particulate matter pollution has emerged as the leading contributor to COPD DALYs globally, particularly in low-income regions such as Sub-Saharan Africa and South Asia. This highlights a growing public health challenge linked to environmental quality and underscores the need for targeted environmental interventions in these regions.

An important dimension of COPD burden not directly captured in this analysis is the temporal trend in exacerbation events, which represent acute episodes of worsening symptoms requiring medical attention. These episodes—whether treated in outpatient settings or resulting in hospital admissions—are key contributors to both patient morbidity and healthcare system strain. Emerging evidence suggests that exacerbation rates and management patterns may have changed significantly during the COVID-19 pandemic (2019–2021), due to altered viral transmission dynamics, shifts in healthcare utilisation and behavioural changes among patients. However, the GBD 2021 dataset does not provide disaggregated data on exacerbation episodes over time. Future work that integrates exacerbation-specific data with long-term trends in COPD prevalence, mortality and DALYs would offer important insights for clinical and policy planning.

To enhance COPD management strategies based on emerging risk factors highlighted in our study, we suggest several policy recommendations. First, implement regional action plans to mitigate the impact of particulate matter pollution, particularly in the areas in which it disproportionately causes COPD. This could involve stricter emission controls and incentives for clean technologies.^37 38^ Second, strengthen surveillance systems to monitor air quality and health outcomes related to COPD.^39^ Enhanced data collection is crucial for evaluating the effectiveness of interventions and adapting policies as needed.^40^ Third, develop cross-sectoral health strategies that combine COPD management with broader health initiatives targeting related conditions.^41^ This integrated approach can optimise resource use and improve overall health outcomes.^42^ Fourth, support the creation of a global framework to guide and standardise air quality improvement efforts. This could help align international efforts and provide necessary support to regions lacking infrastructure.^43 44^

Given substantial regional heterogeneity in COPD burden and resource availability, a stratified intervention framework is warranted. Aligned with WHO’s recommended ‘best buys’ for non-communicable diseases,^45^ priority in low-resource, high-burden settings such as South Asia and Sub-Saharan Africa should focus on scalable, cost-effective measures, including clean household energy promotion,^46^ tobacco taxation and integration of COPD diagnosis and treatment into primary care.^47^ Ensuring access to essential inhaled medications, such as bronchodilators and corticosteroids, remains critical.^47^ In contrast, high-income regions, where mortality is lower but prevalence remains elevated, should prioritise long-term disease management and pulmonary rehabilitation. Upper-middle-income countries with rising EAPCs may benefit from enhanced surveillance, public awareness campaigns and workforce training. This tiered approach enables efficient allocation of limited resources while maximising population-level health gains.

### Limitations

Our study provides important insights into COPD trends and risk factors, yet there are several limitations to consider. First, the reliance on a restricted number of high-quality epidemiological databases may limit the representativeness of our findings, particularly for regions with underdeveloped health data infrastructures. The GBD approach underpinning the data used for this study employs complex modelling techniques to provide estimates for area-time combinations where data are sparse or nonexistent. However, the reliability of these estimates, especially in data-poor regions, remains uncertain, as do the UIs provided for these estimates. While this framework ensures consistency across countries and years, we did not perform independent sensitivity analyses to assess how variations in modelling assumptions might influence our results. Second, the survey data used in our study are subject to variability in how COPD was measured and diagnosed over time and across different regions, potentially impacting the consistency and comparability of our findings. Third, while we included various known risk factors for COPD, it is important to note that most of these associations have not been established through randomised controlled trials. As a result, the causal relationship between these risk factors and COPD remains unclear, with the possibility of residual confounding influencing the observed associations (to a greater extent air pollution, and to a lesser extent tobacco smoking). Additionally, genetic predispositions and other less quantifiable factors, which could significantly affect COPD susceptibility and severity, were not accounted for in our analysis. Fifth, the underdiagnosis of COPD due to limited access to diagnostic tools such as spirometry, particularly in resource-constrained settings, could lead to an underestimation of the true prevalence and severity of COPD. This limitation underscores the need for improved diagnostic capabilities and standardised measurement approaches in future research to ensure more accurate assessments of the burden of COPD.

## Conclusions

This study conducted a comprehensive analysis of COPD from 1990 to 2021, providing updated insights into the prevalence, mortality and major risk factors of COPD at global, regional and national levels. A significant finding from this research is the emergence of particulate matter pollution as the leading risk factor for COPD globally, surpassing smoking, particularly in low-income regions where it is responsible for the majority of COPD burden. This shift emphasises the evolving landscape of COPD risk factors and the critical need for integrated public health strategies that address environmental factors alongside traditional factors like smoking. The implications of these findings are profound, highlighting the necessity for policymakers to adapt and innovate public health interventions that are responsive to these changing dynamics. By doing so, it is possible to enhance healthcare delivery, improve disease management and ultimately reduce the healthcare burden of COPD, thereby contributing to the overall improvement of global health systems.

## Supplementary material

10.1136/bmjph-2024-002489 online supplemental file 1

## Data Availability

Data are available in a public, open access repository.
