# Supplementary material for: Burden of chronic obstructive pulmonary disease and its attributable risk factors in 204 countries and territories, 1990–2021: results from the Global Burden of Disease Study 2021
Source: BMJ Public Health. 2026 Jan 13;4(1):e002489. doi: 10.1136/bmjph-2024-002489 (PMC12815125; doi:10.1136/bmjph-2024-002489)
Supplement: online supplemental file 1 [file bmjph-4-1-s001.pdf]

Supplemental Materials

Table S1. Age standardised prevalence rate, EAPC for ASRs of COPD at national level, 1990-2021 and 2019-2021. 2
Table S2. Age standardised death rate, EAPC for ASRs of COPD at national level, 1990-2021 and 2019-2021. 17
Table S3. Age standardised DALY rates, EAPC for ASRs of COPD at national level, 1990-2021 and 2019-2021 30
Table S4. The percent in all-cause prevalence and EAPC for percent of COPD at national level, 1990-2021 and 2019-2021. 44
Table S5. The percent in all-cause deaths and EAPC for percent of COPD at national level, 1990-2021 and 2019-2021. 53
Table S6. The percent in all-cause DALYs and EAPC for percent of COPD at national level, 1990-2021 and 2019-2021. 62

Table S1. Age standardised prevalence rate, EAPC for ASRs of COPD at national level, 1990-2021 and 2019-2021.

location	No, in 2021 (95% UI)	ASRs, per 100,000, in 2021 (95% UI)	EAPC for ASRs from 1990 to 2021 (95% CI)	EAPC for ASRs from 2019 to 2021 (95% CI)
Afghanistan	293963.5 (265452.6 to 325421.0)	2590.1 (2315.1 to 2913.8)	0.168 (0.143 to 0.194)	0.840 (0.438 to 1.243)
Albania	100991.0 (87982.8 to 115685.7)	2389.6 (2092.1 to 2724.8)	0.143 (0.058 to 0.229)	0.016 (-0.136 to 0.168)
Algeria	928242.6 (819663.2 to 1038356.8)	2643.6 (2331.7 to 2954.4)	0.623 (0.570 to 0.676)	0.894 (0.804 to 0.984)
American Samoa	895.9 (795.9 to 1007.4)	1975.3 (1744.8 to 2226.5)	-0.522 (-0.543 to -0.501)	0.209 (-0.192 to 0.611)
Andorra	4031.0 (3596.3 to 4534.6)	2655.2 (2387.7 to 2982.3)	-0.285 (-0.313 to -0.256)	-0.379 (-0.456 to -0.301)
Angola	216940.0 (189977.9 to 243494.3)	1672.1 (1444.0 to 1899.1)	-0.296 (-0.344 to -0.248)	1.227 (0.769 to 1.687)
Antigua and Barbuda	1356.5 (1164.0 to 1556.6)	1325.1 (1134.4 to 1515.8)	0.871 (0.822 to 0.920)	0.471 (0.306 to 0.637)
Argentina	892476.0 (798704.4 to 999970.6)	1601.3 (1436.5 to 1793.4)	0.054 (-0.036 to 0.143)	-0.466 (-0.844 to -0.086)
Armenia	100139.0 (89041.9 to 113173.6)	2370.3 (2110.4 to 2680.1)	-0.007 (-0.037 to 0.022)	-0.619 (-1.933 to 0.713)
Australia	706604.8 (624380.4 to 818768.8)	1562.1 (1386.0 to 1789.9)	-0.922 (-1.009 to -0.835)	-2.603 (-4.981 to -0.165)
Austria	511218.6 (457335.1 to 558680.5)	2891.9 (2593.5 to 3153.6)	0.004 (-0.118 to 0.127)	-0.812 (-1.649 to 0.031)
Azerbaijan	216263.7 (190401.1 to 245357.6)	2246.7 (1967.7 to 2555.5)	0.026 (-0.033 to 0.085)	0.016 (-0.057 to 0.090)
Bahamas	5236.4 (4613.8 to 5896.7)	1320.9 (1167.5 to 1488.5)	0.580 (0.522 to 0.638)	-0.466 (-1.130 to 0.204)
Bahrain	23865.4 (21039.3 to	2547.6 (2251.2 to 2882.7)	-0.005 (-0.050 to 0.041)	0.497 (-0.529 to 1.534)

	27392.5)			
Bangladesh	4020093.9 (3629428.1 to 4477582.4)	2956.2 (2669.1 to 3284.8)	0.110 (0.010 to 0.210)	0.206 (-0.099 to 0.512)
Barbados	6436.9 (5548.6 to 7310.6)	1305.4 (1133.3 to 1483.3)	0.720 (0.679 to 0.760)	0.129 (-0.587 to 0.850)
Belarus	312912.3 (275571.5 to 354243.7)	2046.9 (1814.3 to 2305.1)	-0.708 (-0.771 to -0.645)	0.258 (0.107 to 0.410)
Belgium	560791.9 (504009.1 to 628348.7)	2469.8 (2225.4 to 2749.4)	-0.035 (-0.089 to 0.019)	-0.915 (-1.153 to -0.677)
Belize	5399.0 (4748.1 to 6049.3)	1746.8 (1525.5 to 1967.8)	0.661 (0.639 to 0.683)	-0.385 (-0.558 to -0.211)
Benin	105518.5 (93284.7 to 117104.5)	1705.2 (1495.9 to 1929.7)	0.146 (0.097 to 0.196)	0.890 (0.862 to 0.918)
Bermuda	1999.9 (1710.9 to 2289.6)	1521.8 (1309.7 to 1727.1)	0.465 (0.384 to 0.546)	-0.245 (-0.724 to 0.237)
Bhutan	16900.7 (15258.0 to 18836.4)	2772.6 (2481.1 to 3092.1)	-0.266 (-0.286 to -0.245)	-0.220 (-0.758 to 0.322)
Bolivia (Plurinational State of)	173920.3 (152345.7 to 197808.9)	1967.4 (1724.6 to 2233.8)	0.102 (0.067 to 0.138)	0.327 (0.031 to 0.625)
Bosnia and Herzegovina	154732.5 (137494.0 to 174491.9)	2584.7 (2311.3 to 2897.0)	0.145 (0.122 to 0.169)	0.014 (-0.871 to 0.908)
Botswana	31547.6 (27926.0 to 35450.5)	2016.9 (1774.2 to 2287.5)	0.027 (-0.055 to 0.108)	0.860 (0.816 to 0.905)
Brazil	6314694.4 (5587560.5 to 7077063.9)	2556.0 (2259.5 to 2860.4)	-0.168 (-0.214 to -0.121)	0.540 (-0.676 to 1.771)
Brunei Darussalam	5191.6 (4569.5 to 5810.0)	1662.1 (1457.5 to 1879.3)	-1.153 (-1.200 to -1.106)	-0.441 (-0.534 to -0.349)
Bulgaria	334019.5 (289512.3 to 380136.6)	2453.2 (2164.2 to 2774.1)	-0.027 (-0.058 to 0.005)	0.436 (-0.624 to 1.507)
Burkina Faso	172987.3 (153466.2 to	1608.1 (1425.7 to 1828.6)	0.237 (0.191 to 0.282)	0.899 (0.331 to 1.471)

	193704.5)			
Burundi	109735.9 (97674.0 to 121235.8)	1878.3 (1651.0 to 2110.6)	0.154 (0.090 to 0.218)	0.769 (0.756 to 0.782)
Cabo Verde	4804.9 (4201.1 to 5403.5)	1001.3 (872.5 to 1138.0)	0.047 (-0.055 to 0.150)	0.885 (0.742 to 1.027)
Cambodia	272673.5 (243081.4 to 308825.3)	2230.7 (1969.9 to 2529.5)	0.066 (0.033 to 0.099)	0.569 (0.476 to 0.661)
Cameroon	239814.7 (213803.8 to 269209.0)	1574.0 (1383.4 to 1800.3)	0.077 (0.017 to 0.137)	1.125 (0.636 to 1.617)
Canada	1522991.4 (1358067.6 to 1700360.2)	2098.4 (1880.9 to 2351.5)	-0.199 (-0.491 to 0.093)	-0.490 (-1.778 to 0.816)
Central African Republic	52024.1 (45816.9 to 57955.1)	2145.3 (1877.7 to 2435.8)	0.291 (0.257 to 0.324)	0.988 (0.972 to 1.005)
Chad	129102.4 (114268.6 to 144157.9)	1857.6 (1625.0 to 2113.5)	0.291 (0.228 to 0.353)	1.103 (0.703 to 1.505)
Chile	310100.3 (269434.1 to 353782.8)	1213.7 (1060.3 to 1388.8)	-0.570 (-0.603 to -0.536)	-0.736 (-1.853 to 0.393)
China	50588429.3 (44975892.8 to 57116835.2)	2499.3 (2236.2 to 2793.3)	-0.333 (-0.373 to -0.293)	-0.333 (-0.634 to -0.031)
Colombia	1306282.9 (1176060.5 to 1451413.0)	2340.9 (2106.1 to 2604.2)	-0.091 (-0.166 to -0.017)	0.259 (0.059 to 0.458)
Comoros	7791.5 (6800.1 to 8782.8)	1492.5 (1300.0 to 1696.4)	-0.128 (-0.194 to -0.061)	1.016 (0.508 to 1.527)
Congo	49376.3 (43371.4 to 55552.5)	1717.0 (1501.9 to 1954.9)	0.091 (0.027 to 0.155)	1.147 (0.764 to 1.531)
Cook Islands	447.7 (379.0 to 512.7)	1821.4 (1557.9 to 2077.0)	-0.546 (-0.585 to -0.507)	0.317 (-0.275 to 0.911)
Costa Rica	115582.7 (100152.8 to 129868.6)	2098.8 (1814.8 to 2368.3)	0.218 (0.173 to 0.262)	0.615 (0.183 to 1.049)

Croatia	222941.3 (199359.8 to 246696.4)	2553.3 (2289.7 to 2833.7)	0.497 (0.473 to 0.521)	0.242 (0.135 to 0.350)
Cuba	409598.0 (365089.0 to 455411.1)	2139.4 (1919.7 to 2374.5)	0.761 (0.659 to 0.864)	-0.990 (-1.953 to -0.018)
Cyprus	50059.6 (44353.0 to 56084.2)	2496.4 (2221.8 to 2781.1)	-0.223 (-0.253 to -0.193)	-0.477 (-0.560 to -0.393)
Czechia	516786.5 (462606.7 to 566688.1)	2498.7 (2264.6 to 2734.9)	0.584 (0.564 to 0.603)	-0.089 (-0.112 to -0.067)
C 么 te d'Ivoire	230232.1 (204519.0 to 259208.9)	1691.7 (1489.9 to 1934.8)	0.310 (0.239 to 0.382)	0.956 (0.437 to 1.479)
Democratic People's Republic of Korea	879586.6 (791149.1 to 981320.4)	2757.5 (2487.1 to 3066.8)	-0.485 (-0.534 to -0.435)	-0.448 (-0.701 to -0.193)
Democratic Republic of the Congo	807959.0 (717254.4 to 913626.8)	2029.8 (1800.5 to 2308.6)	0.449 (0.427 to 0.471)	0.933 (0.609 to 1.258)
Denmark	310720.0 (275454.3 to 344044.3)	2733.0 (2448.7 to 3009.8)	-0.367 (-0.423 to -0.310)	-0.192 (-0.469 to 0.085)
Djibouti	10503.9 (9075.5 to 11843.9)	1412.2 (1230.0 to 1602.9)	0.114 (-0.032 to 0.259)	1.294 (1.179 to 1.409)
Dominica	1176.7 (1004.9 to 1352.3)	1451.2 (1241.7 to 1668.9)	0.404 (0.389 to 0.420)	0.404 (0.026 to 0.783)
Dominican Republic	169699.0 (147744.0 to 193588.2)	1680.8 (1452.7 to 1916.9)	0.773 (0.748 to 0.797)	0.072 (-0.347 to 0.494)
Ecuador	281618.7 (246355.0 to 320213.2)	1737.5 (1521.9 to 1977.8)	0.230 (0.191 to 0.269)	0.032 (-0.153 to 0.218)
Egypt	1555374.1 (1364099.2 to 1775225.2)	2472.7 (2161.7 to 2806.8)	0.858 (0.834 to 0.882)	0.787 (0.261 to 1.316)
El Salvador	120349.2 (104289.6 to	1855.2 (1599.7 to 2125.4)	0.274 (0.242 to 0.307)	0.406 (-0.190 to 1.005)

	137641.9)			
Equatorial Guinea	10267.1 (8986.2 to 11530.8)	1732.1 (1522.7 to 1979.5)	-0.200 (-0.346 to -0.054)	1.100 (0.887 to 1.315)
Eritrea	51716.3 (45557.8 to 58726.0)	1543.6 (1344.2 to 1764.9)	-0.056 (-0.129 to 0.016)	1.075 (0.412 to 1.741)
Estonia	39751.1 (35068.2 to 44930.3)	1532.4 (1367.8 to 1728.9)	0.710 (0.623 to 0.797)	-3.046 (-5.240 to -0.801)
Eswatini	11741.7 (10401.9 to 13112.7)	1921.6 (1707.8 to 2173.5)	-0.092 (-0.163 to -0.021)	0.704 (0.270 to 1.139)
Ethiopia	890979.3 (793351.8 to 987246.8)	1715.3 (1509.2 to 1918.4)	-0.296 (-0.363 to -0.230)	0.978 (0.503 to 1.454)
Fiji	12013.7 (10576.5 to 13795.0)	1655.8 (1446.4 to 1890.8)	-0.827 (-0.866 to -0.788)	-0.037 (-0.486 to 0.413)
Finland	256967.7 (224690.9 to 292252.3)	2133.0 (1886.9 to 2393.4)	-0.136 (-0.205 to -0.067)	0.105 (-0.410 to 0.623)
France	2969242.8 (2646069.7 to 3337983.2)	2182.5 (1950.2 to 2438.8)	-0.064 (-0.118 to -0.010)	-0.591 (-0.861 to -0.320)
Gabon	16523.9 (14379.9 to 18762.2)	1530.9 (1330.6 to 1767.8)	0.287 (0.181 to 0.392)	1.372 (0.992 to 1.753)
Gambia	20616.1 (18392.7 to 22929.1)	1778.3 (1574.4 to 2005.2)	0.302 (0.248 to 0.356)	0.906 (0.651 to 1.161)
Georgia	121326.5 (105831.5 to 137326.1)	2091.9 (1845.0 to 2352.7)	0.551 (0.496 to 0.606)	0.167 (-0.252 to 0.587)
Germany	5218529.3 (4717214.5 to 5793017.9)	2759.6 (2508.8 to 3043.4)	-0.107 (-0.213 to -0.002)	-0.542 (-1.357 to 0.280)
Ghana	320571.8 (285498.2 to	1672.3 (1471.4 to 1876.4)	0.544 (0.488 to 0.600)	0.983 (0.778 to 1.189)

	355226.7)			
Greece	609189.7 (546308.3 to 683100.1)	2575.7 (2325.3 to 2864.4)	-0.004 (-0.029 to 0.021)	-0.432 (-1.161 to 0.303)
Greenland	1677.2 (1516.2 to 1867.8)	2542.8 (2306.1 to 2822.9)	-0.237 (-0.259 to -0.215)	-0.457 (-1.104 to 0.194)
Grenada	1630.2 (1424.6 to 1831.3)	1482.2 (1299.2 to 1658.7)	0.529 (0.480 to 0.577)	-0.022 (-0.117 to 0.073)
Guam	3391.8 (2875.4 to 3841.5)	1623.6 (1387.7 to 1836.3)	-0.392 (-0.433 to -0.351)	0.100 (-0.081 to 0.281)
Guatemala	209544.5 (186412.2 to 236785.8)	1906.1 (1694.1 to 2164.4)	0.064 (0.005 to 0.123)	0.619 (0.523 to 0.715)
Guinea	119666.6 (106166.6 to 132601.5)	1842.2 (1625.6 to 2067.6)	0.424 (0.370 to 0.477)	1.066 (0.819 to 1.312)
Guinea-Bissau	16064.6 (14308.0 to 17918.3)	1757.8 (1541.2 to 2001.5)	0.183 (0.096 to 0.271)	0.733 (0.153 to 1.316)
Guyana	8911.7 (7696.9 to 10104.9)	1411.3 (1215.3 to 1606.0)	0.546 (0.510 to 0.581)	0.477 (-0.310 to 1.271)
Haiti	142144.5 (125213.2 to 159936.4)	1909.4 (1687.8 to 2174.3)	0.150 (0.101 to 0.198)	0.152 (0.079 to 0.226)
Honduras	157513.4 (139565.2 to 179516.7)	2521.8 (2240.3 to 2870.9)	0.513 (0.466 to 0.561)	0.216 (-0.042 to 0.474)
Hungary	548104.4 (491546.8 to 598032.2)	2933.7 (2673.6 to 3185.9)	0.487 (0.463 to 0.510)	-0.584 (-0.720 to -0.449)
Iceland	15732.0 (14101.9 to 17342.0)	2769.6 (2492.5 to 3053.5)	-0.476 (-0.614 to -0.338)	-0.214 (-0.262 to -0.165)
India	35819854.9 (32396904.2 to 39076078.8)	3067.9 (2782.5 to 3347.9)	0.130 (0.108 to 0.153)	-0.244 (-0.376 to -0.111)
Indonesia	5080725.2 (4434330.2 to 5713205.4)	2204.8 (1930.5 to 2481.7)	0.319 (0.303 to 0.334)	0.559 (0.330 to 0.788)
Iran (Islamic Republic of)	1586139.3 (1396410.6 to	2054.6 (1798.4 to 2321.9)	0.756 (0.731 to 0.782)	0.096 (-0.315 to 0.509)

	1781229.4)			
Iraq	494470.0 (435909.9 to 559676.3)	2003.4 (1756.2 to 2283.5)	0.599 (0.554 to 0.644)	1.053 (0.124 to 1.991)
Ireland	191319.4 (172627.3 to 213047.6)	2466.8 (2233.2 to 2735.7)	-0.423 (-0.438 to -0.408)	0.326 (0.236 to 0.415)
Israel	283970.3 (250022.6 to 317251.1)	2340.1 (2084.0 to 2602.8)	-0.340 (-0.365 to -0.314)	-0.455 (-0.791 to -0.118)
Italy	3174977.9 (2745407.6 to 3585532.4)	2159.4 (1891.0 to 2433.8)	-0.169 (-0.207 to -0.131)	-0.675 (-1.476 to 0.133)
Jamaica	52551.8 (47134.4 to 58583.5)	1653.9 (1478.4 to 1848.0)	0.333 (0.270 to 0.397)	0.309 (-0.030 to 0.649)
Japan	5114116.6 (4309205.5 to 6010382.1)	1285.5 (1093.0 to 1492.2)	-0.752 (-0.827 to -0.678)	-0.562 (-1.334 to 0.216)
Jordan	165854.3 (145994.2 to 186678.4)	2153.0 (1877.4 to 2442.7)	0.171 (0.152 to 0.191)	0.539 (0.214 to 0.865)
Kazakhstan	474247.1 (429214.2 to 532108.6)	2778.2 (2508.1 to 3109.0)	0.471 (0.350 to 0.591)	-0.094 (-0.208 to 0.020)
Kenya	403347.0 (352452.0 to 452161.1)	1528.5 (1330.3 to 1716.1)	0.131 (0.079 to 0.183)	0.856 (0.383 to 1.331)
Kiribati	1668.5 (1494.0 to 1876.6)	2277.9 (1999.0 to 2574.5)	-0.101 (-0.119 to -0.084)	-0.006 (-0.392 to 0.383)
Kuwait	66467.3 (58682.9 to 74860.7)	2006.9 (1773.1 to 2282.4)	0.639 (0.623 to 0.655)	0.538 (0.387 to 0.688)
Kyrgyzstan	125202.3 (111803.3 to 140538.4)	2653.9 (2332.3 to 2976.5)	-0.502 (-0.552 to -0.452)	-0.073 (-0.415 to 0.271)
Lao People's Democratic Republic	113895.9 (102552.5 to 127023.5)	2401.4 (2142.0 to 2705.2)	-0.197 (-0.244 to -0.150)	0.739 (0.179 to 1.301)

Latvia	67917.2 (59522.1 to 76700.0)	1801.3 (1589.7 to 2021.8)	-0.052 (-0.156 to 0.053)	0.532 (0.529 to 0.534)
Lebanon	153048.2 (132931.7 to 174115.2)	2481.2 (2159.6 to 2823.2)	1.008 (0.952 to 1.064)	-1.136 (-2.796 to 0.553)
Lesotho	26389.8 (23470.8 to 29591.7)	2334.7 (2076.7 to 2646.7)	0.234 (0.175 to 0.294)	0.759 (0.362 to 1.158)
Liberia	43723.9 (38564.6 to 49060.8)	1718.2 (1498.1 to 1946.9)	0.523 (0.481 to 0.565)	0.808 (0.613 to 1.002)
Libya	121288.9 (107865.8 to 136197.3)	2224.6 (1971.3 to 2518.3)	0.764 (0.739 to 0.788)	-0.201 (-1.007 to 0.612)
Lithuania	103040.9 (91249.9 to 116814.2)	1875.0 (1673.2 to 2112.1)	-0.540 (-0.610 to -0.471)	0.607 (0.478 to 0.737)
Luxembourg	26888.0 (24038.9 to 29849.9)	2570.6 (2309.6 to 2854.1)	-0.228 (-0.287 to -0.168)	-0.344 (-0.426 to -0.262)
Madagascar	248160.4 (221119.6 to 277578.2)	1858.0 (1648.4 to 2120.9)	0.349 (0.312 to 0.387)	0.634 (-0.170 to 1.445)
Malawi	136848.3 (120542.7 to 154114.2)	1530.9 (1352.1 to 1728.7)	0.478 (0.434 to 0.522)	1.136 (0.961 to 1.312)
Malaysia	499394.9 (433574.3 to 562409.7)	1784.4 (1540.8 to 2022.6)	0.052 (0.035 to 0.070)	0.692 (0.388 to 0.997)
Maldives	8257.0 (7362.1 to 9221.5)	2268.2 (1999.5 to 2573.5)	-0.417 (-0.466 to -0.367)	0.766 (0.173 to 1.363)
Mali	194654.7 (172830.4 to 214824.2)	1845.7 (1626.5 to 2078.3)	0.337 (0.280 to 0.395)	1.083 (0.980 to 1.186)
Malta	19680.6 (17070.5 to 22217.9)	2133.0 (1879.6 to 2381.1)	-0.429 (-0.482 to -0.376)	-0.215 (-0.230 to -0.200)
Marshall Islands	733.3 (661.4 to 814.4)	2024.1 (1797.4 to 2296.1)	-0.425 (-0.452 to -0.397)	0.264 (-0.072 to 0.601)

Mauritania	35872.1 (31656.0 to 40877.5)	1500.8 (1316.6 to 1725.3)	0.229 (0.145 to 0.312)	1.239 (0.843 to 1.637)
Mauritius	29226.7 (25865.6 to 33113.1)	1687.1 (1499.8 to 1901.1)	-0.059 (-0.126 to 0.009)	0.571 (0.554 to 0.588)
Mexico	2747439.2 (2421692.7 to 3093515.2)	2238.9 (1970.8 to 2525.8)	0.317 (0.233 to 0.401)	-0.981 (-1.911 to -0.042)
Micronesia (Federated States of)	1616.7 (1462.1 to 1822.8)	2211.0 (1956.1 to 2493.2)	-0.398 (-0.422 to -0.374)	0.138 (0.039 to 0.237)
Monaco	2220.1 (1929.1 to 2511.8)	2386.9 (2120.5 to 2666.9)	-0.206 (-0.255 to -0.156)	-0.265 (-0.427 to -0.103)
Mongolia	48865.5 (43338.3 to 54857.4)	2188.0 (1928.7 to 2469.0)	0.004 (-0.026 to 0.034)	-0.247 (-1.071 to 0.584)
Montenegro	18890.0 (16209.1 to 21406.1)	2053.6 (1782.4 to 2305.7)	0.523 (0.481 to 0.564)	-0.152 (-0.803 to 0.504)
Morocco	796614.1 (701585.2 to 903040.6)	2361.4 (2081.7 to 2665.4)	0.979 (0.955 to 1.002)	0.861 (0.273 to 1.453)
Mozambique	210038.3 (184700.1 to 234149.0)	1571.2 (1375.6 to 1771.7)	0.215 (0.161 to 0.269)	1.174 (1.073 to 1.276)
Myanmar	1295568.4 (1176296.9 to 1449782.6)	2719.8 (2465.8 to 3044.7)	-0.136 (-0.154 to -0.118)	0.206 (0.121 to 0.291)
Namibia	29661.2 (26106.5 to 33277.0)	2047.2 (1801.2 to 2328.0)	0.082 (0.018 to 0.146)	0.526 (0.248 to 0.805)
Nauru	130.5 (117.0 to 145.8)	2138.4 (1864.2 to 2421.0)	-0.248 (-0.276 to -0.220)	0.240 (-0.174 to 0.655)
Nepal	710534.5 (659222.2 to 765341.1)	3051.1 (2835.2 to 3271.7)	0.050 (0.002 to 0.099)	-0.271 (-0.415 to -0.127)
Netherlands	759256.7 (682277.2 to 845740.3)	2263.3 (2048.9 to 2513.4)	0.077 (-0.075 to 0.230)	-3.367 (-4.299 to -2.426)

New Zealand	141262.4 (118993.6 to 166031.4)	1678.3 (1429.3 to 1958.6)	-0.522 (-0.621 to -0.423)	-2.071 (-4.131 to 0.033)
Nicaragua	106608.7 (93934.0 to 118461.8)	2223.5 (1965.1 to 2482.7)	0.586 (0.555 to 0.618)	0.377 (-0.699 to 1.466)
Niger	187856.4 (166784.9 to 210573.1)	1946.0 (1710.2 to 2210.0)	0.605 (0.535 to 0.674)	0.894 (0.503 to 1.286)
Nigeria	1897949.3 (1672847.1 to 2120293.1)	1788.6 (1562.9 to 2005.8)	0.455 (0.413 to 0.496)	0.877 (0.786 to 0.967)
Niue	39.4 (34.3 to 45.3)	1920.9 (1678.9 to 2190.6)	-0.520 (-0.545 to -0.494)	0.425 (0.268 to 0.583)
North Macedonia	81263.0 (70963.5 to 94240.9)	2624.1 (2302.9 to 3004.6)	0.259 (0.198 to 0.320)	0.233 (0.219 to 0.247)
Northern Mariana Islands	884.7 (766.6 to 1006.3)	1867.0 (1609.5 to 2151.4)	-0.329 (-0.366 to -0.291)	0.214 (0.168 to 0.259)
Norway	271735.9 (240590.4 to 307387.9)	2768.8 (2464.3 to 3111.1)	0.364 (0.243 to 0.486)	-0.616 (-0.620 to -0.612)
Oman	53844.0 (47442.9 to 61113.0)	2314.6 (2036.6 to 2644.1)	0.287 (0.245 to 0.330)	0.388 (-0.243 to 1.023)
Pakistan	3199227.6 (2839704.8 to 3571679.3)	2606.3 (2311.9 to 2926.0)	-0.028 (-0.062 to 0.006)	-0.252 (-0.557 to 0.054)
Palau	411.6 (361.8 to 464.1)	1988.3 (1735.2 to 2245.2)	-0.418 (-0.444 to -0.392)	0.108 (-0.160 to 0.376)
Palestine	57385.2 (51328.8 to 64186.7)	2147.7 (1912.3 to 2436.2)	0.302 (0.270 to 0.334)	0.949 (0.127 to 1.778)
Panama	83479.6 (75166.9 to 92863.5)	1863.2 (1677.3 to 2075.5)	0.268 (0.206 to 0.330)	1.096 (0.636 to 1.557)
Papua New Guinea	146155.3 (134849.0 to 158586.9)	2711.6 (2480.6 to 2980.5)	-0.165 (-0.178 to -0.152)	0.107 (0.091 to 0.122)
Paraguay	125830.1 (110754.6 to	2153.4 (1889.7 to 2446.9)	0.285 (0.259 to 0.312)	0.661 (-0.144 to 1.473)

	142204.9)			
Peru	523015.7 (451412.9 to 597483.0)	1538.0 (1327.3 to 1755.9)	0.430 (0.296 to 0.564)	-2.899 (-6.129 to 0.441)
Philippines	1835887.9 (1631451.2 to 2046786.6)	2213.4 (1960.9 to 2482.7)	-0.442 (-0.492 to -0.393)	0.328 (0.094 to 0.562)
Poland	1561336.3 (1357982.7 to 1758296.6)	2262.3 (1997.9 to 2539.2)	-0.059 (-0.106 to -0.012)	0.356 (0.207 to 0.505)
Portugal	557534.3 (501098.3 to 625041.9)	2318.0 (2088.2 to 2583.4)	-0.584 (-0.649 to -0.519)	-0.182 (-0.284 to -0.081)
Puerto Rico	128481.4 (111721.5 to 143979.5)	1803.0 (1581.2 to 2026.5)	0.461 (0.377 to 0.546)	-0.030 (-1.139 to 1.091)
Qatar	32092.3 (28315.4 to 36059.0)	2384.5 (2104.2 to 2686.8)	0.345 (0.320 to 0.369)	0.568 (-0.908 to 2.066)
Republic of Korea	2090259.6 (1904590.6 to 2305737.5)	2287.9 (2091.3 to 2510.8)	0.342 (0.087 to 0.599)	-2.137 (-2.557 to -1.715)
Republic of Moldova	117569.8 (102730.1 to 134548.4)	2054.3 (1806.0 to 2331.2)	-0.771 (-0.832 to -0.711)	0.132 (-0.060 to 0.325)
Romania	849850.4 (757217.5 to 951582.6)	2367.7 (2125.9 to 2635.4)	-0.246 (-0.278 to -0.215)	-0.364 (-1.042 to 0.318)
Russian Federation	4982424.3 (4358539.7 to 5631745.8)	2196.0 (1945.5 to 2463.5)	-0.279 (-0.317 to -0.241)	0.961 (0.448 to 1.476)
Rwanda	131916.6 (116357.8 to 146789.6)	1830.3 (1621.7 to 2058.3)	0.082 (0.014 to 0.150)	0.905 (0.847 to 0.963)
Saint Kitts and Nevis	960.6 (824.1 to 1111.0)	1457.7 (1255.8 to 1677.6)	0.780 (0.698 to 0.862)	-0.670 (-1.188 to -0.150)
Saint Lucia	4138.3 (3601.0 to 4647.2)	1761.2 (1536.1 to 1974.4)	0.462 (0.415 to 0.510)	0.220 (-0.364 to 0.808)
Saint Vincent and the	1811.4 (1582.8 to 2061.0)	1314.2 (1156.1 to 1497.4)	0.633 (0.609 to 0.656)	-0.107 (-1.121 to 0.918)

Grenadines				
Samoa	3302.8 (2952.4 to 3714.3)	2293.9 (2022.6 to 2584.2)	-0.402 (-0.426 to -0.378)	0.274 (-0.191 to 0.741)
San Marino	1604.2 (1389.9 to 1828.4)	2154.3 (1897.8 to 2430.9)	-0.369 (-0.388 to -0.351)	-0.324 (-1.063 to 0.421)
Sao Tome and Principe	2647.3 (2401.8 to 2927.2)	2084.5 (1858.3 to 2339.8)	0.501 (0.463 to 0.538)	0.611 (0.407 to 0.816)
Saudi Arabia	448933.4 (391042.3 to 508906.2)	2050.9 (1796.4 to 2325.3)	1.076 (1.045 to 1.107)	1.198 (0.091 to 2.318)
Senegal	146764.8 (129857.5 to 165174.2)	1680.3 (1479.9 to 1901.3)	0.299 (0.234 to 0.364)	0.577 (0.398 to 0.757)
Serbia	429424.6 (384715.4 to 479782.1)	2692.5 (2420.3 to 2989.3)	0.452 (0.415 to 0.489)	-0.059 (-0.194 to 0.076)
Seychelles	2072.1 (1811.7 to 2356.4)	1835.8 (1598.8 to 2078.8)	0.279 (0.231 to 0.327)	0.668 (0.128 to 1.210)
Sierra Leone	79279.2 (70634.8 to 88256.4)	1770.4 (1550.6 to 1987.3)	0.284 (0.227 to 0.340)	1.143 (0.540 to 1.749)
Singapore	77467.3 (67346.4 to 89381.8)	922.6 (805.9 to 1060.5)	-1.631 (-1.716 to -1.545)	-0.597 (-0.811 to -0.384)
Slovakia	192936.6 (169685.0 to 218388.7)	2118.5 (1878.4 to 2394.7)	0.375 (0.357 to 0.392)	-0.319 (-1.203 to 0.573)
Slovenia	94845.7 (83121.9 to 106777.1)	2177.7 (1923.8 to 2437.0)	0.031 (0.015 to 0.047)	-0.390 (-1.383 to 0.613)
Solomon Islands	9027.2 (8290.0 to 9978.0)	2490.6 (2242.1 to 2783.8)	-0.257 (-0.269 to -0.245)	0.100 (-0.189 to 0.390)
Somalia	163129.7 (146092.7 to 181015.9)	2116.8 (1858.6 to 2404.2)	0.393 (0.332 to 0.453)	1.103 (1.003 to 1.203)
South Africa	1004761.5 (886683.6 to 1119026.9)	2170.5 (1917.5 to 2437.2)	-0.060 (-0.110 to -0.009)	0.207 (-0.041 to 0.455)
South Sudan	71608.4 (63234.7 to 80531.1)	1601.0 (1404.9 to 1818.4)	0.165 (0.097 to 0.232)	1.096 (0.919 to 1.272)

Spain	2570070.7 (2308173.7 to 2861069.5)	2658.2 (2409.1 to 2950.5)	-0.193 (-0.264 to -0.123)	-0.593 (-1.233 to 0.052)
Sri Lanka	495569.1 (428210.9 to 568689.2)	1923.1 (1670.3 to 2194.0)	0.137 (0.117 to 0.156)	0.842 (0.752 to 0.931)
Sudan	518223.1 (462333.7 to 578783.9)	2416.1 (2114.4 to 2730.3)	0.015 (-0.027 to 0.058)	0.662 (0.020 to 1.308)
Suriname	9822.2 (8647.0 to 11101.2)	1582.0 (1391.6 to 1787.3)	0.372 (0.324 to 0.421)	0.371 (0.055 to 0.689)
Sweden	656865.6 (580693.5 to 731419.8)	3097.6 (2760.3 to 3448.7)	-0.102 (-0.197 to -0.007)	-0.107 (-0.254 to 0.041)
Switzerland	451983.3 (408103.4 to 501311.8)	2549.0 (2306.3 to 2820.5)	-0.381 (-0.485 to -0.278)	-0.828 (-1.162 to -0.493)
Syrian Arab Republic	307654.0 (271843.5 to 349197.8)	2446.6 (2166.8 to 2749.1)	0.677 (0.645 to 0.708)	0.565 (-0.074 to 1.208)
Taiwan (Province of China)	624948.9 (546926.1 to 716439.7)	1512.2 (1331.3 to 1725.4)	-0.861 (-0.932 to -0.790)	-0.714 (-1.607 to 0.188)
Tajikistan	149563.1 (134319.3 to 167205.8)	2519.4 (2214.1 to 2838.9)	-0.026 (-0.049 to -0.004)	0.207 (-0.050 to 0.463)
Thailand	1781016.6 (1544547.2 to 2028248.1)	1711.0 (1490.5 to 1934.2)	-0.702 (-0.729 to -0.676)	-1.012 (-3.205 to 1.231)
Timor-Leste	19723.1 (17602.4 to 22257.9)	2295.4 (2041.9 to 2596.0)	-0.210 (-0.263 to -0.157)	0.490 (0.063 to 0.918)
Togo	77409.9 (67732.9 to 86985.9)	1725.8 (1505.5 to 1960.3)	0.266 (0.213 to 0.319)	0.900 (0.796 to 1.004)
Tokelau	27.7 (24.1 to 31.5)	1887.8 (1641.6 to 2144.3)	-0.557 (-0.588 to -0.526)	0.198 (0.169 to 0.227)
Tonga	1578.0 (1380.0 to 1791.2)	1951.3 (1690.3 to 2217.2)	-0.644 (-0.668 to -0.620)	0.307 (-0.121 to 0.736)
Trinidad and Tobago	26719.0 (23035.9 to	1434.7 (1240.9 to 1640.3)	0.325 (0.310 to 0.340)	0.749 (0.572 to 0.927)

	30689.7)			
Tunisia	362516.5 (323629.1 to 405804.5)	2808.2 (2494.1 to 3138.1)	0.703 (0.666 to 0.740)	0.258 (-0.351 to 0.870)
Turkmenistan	66967.5 (59373.4 to 75767.2)	1681.0 (1478.8 to 1909.5)	-0.943 (-1.042 to -0.845)	0.480 (-0.371 to 1.338)
Tuvalu	213.5 (189.1 to 242.7)	2094.6 (1836.1 to 2383.5)	-0.519 (-0.547 to -0.491)	0.376 (-0.128 to 0.882)
T 眉 rkiye	2885480.5 (2627311.6 to 3185404.7)	3146.7 (2861.3 to 3477.8)	0.316 (0.242 to 0.389)	0.061 (-0.328 to 0.453)
Uganda	297985.2 (265625.6 to 332702.1)	1663.1 (1467.4 to 1879.9)	-0.101 (-0.157 to -0.046)	0.986 (0.686 to 1.286)
Ukraine	1457113.6 (1254134.5 to 1665203.8)	1978.6 (1720.7 to 2252.7)	-1.060 (-1.100 to -1.021)	0.223 (0.129 to 0.316)
United Arab Emirates	180019.8 (161070.9 to 203394.6)	2686.4 (2402.0 to 3008.3)	0.381 (0.341 to 0.420)	0.514 (-0.651 to 1.693)
United Kingdom	4157373.2 (3745187.2 to 4554835.6)	3270.3 (2957.0 to 3570.4)	0.169 (0.125 to 0.213)	-1.069 (-1.797 to -0.336)
United Republic of Tanzania	456371.5 (401902.8 to 513570.6)	1538.2 (1356.2 to 1744.4)	0.246 (0.174 to 0.318)	1.302 (1.298 to 1.306)
United States Virgin Islands	2557.2 (2201.8 to 2931.8)	1478.4 (1288.3 to 1679.2)	0.691 (0.660 to 0.723)	0.287 (0.222 to 0.352)
United States of America	19969706.5 (18951147.4 to 20892544.0)	3445.3 (3263.5 to 3602.4)	0.319 (0.205 to 0.433)	-0.403 (-0.621 to -0.184)
Uruguay	84284.3 (73376.3 to 95642.8)	1553.8 (1365.2 to 1751.8)	0.091 (0.015 to 0.168)	-1.252 (-2.623 to 0.140)
Uzbekistan	478246.5 (425669.4 to 536721.5)	1880.9 (1663.8 to 2124.8)	-0.544 (-0.571 to -0.516)	-0.115 (-0.138 to -0.092)

Vanuatu	4179.3 (3771.1 to 4619.9)	2322.9 (2051.1 to 2603.2)	-0.365 (-0.375 to -0.354)	0.215 (0.115 to 0.315)
Venezuela (Bolivarian Republic of)	616813.5 (554664.0 to 695928.1)	2120.8 (1898.0 to 2390.9)	0.725 (0.660 to 0.790)	0.445 (-0.810 to 1.716)
Viet Nam	2203220.6 (1961863.9 to 2483948.2)	2291.3 (2035.1 to 2592.2)	0.344 (0.299 to 0.389)	0.927 (0.358 to 1.500)
Yemen	380327.8 (339852.7 to 423976.2)	2478.5 (2169.7 to 2808.7)	-0.023 (-0.089 to 0.043)	0.614 (0.165 to 1.064)
Zambia	132605.1 (116576.4 to 148088.0)	1547.5 (1346.3 to 1774.2)	0.172 (0.105 to 0.239)	1.418 (1.175 to 1.662)
Zimbabwe	135132.3 (118220.3 to 153081.7)	1798.6 (1573.6 to 2076.4)	0.200 (0.102 to 0.298)	1.142 (0.043 to 2.254)

EAPC: Estimated Annual Percentage Change; ASRs: Age-standardized rates; COPD: Chronic Obstructive Pulmonary Disease; EAPCs for ASRs from 1990 to 2019 (%, 95% UI) were calculated using GBD 2021.

Table S2. Age standardised death rate, EAPC for ASRs of COPD at national level, 1990-2021 and 2019-2021.

location	No, in 2021 (95% UI)	ASRs, per 100,000, in 2021 (95% UI)	EAPC for ASRs from 1990 to 2021 (95% CI)	EAPC for ASRs from 2019 to 2021 (95% CI)
Afghanistan	3804.6 (2729.0 to 4947.8)	50.6 (36.1 to 65.6)	-0.796 (-1.010 to -0.581)	-0.882 (-1.958 to 0.207)
Albania	650.3 (503.0 to 880.3)	15.6 (11.9 to 21.0)	-3.105 (-3.524 to -2.684)	-1.184 (-4.836 to 2.608)
Algeria	5316.0 (4148.1 to 6533.8)	22.1 (17.4 to 27.3)	-0.027 (-0.176 to 0.122)	-1.221 (-1.515 to -0.926)
American Samoa	17.9 (15.3 to 21.0)	48.7 (41.6 to 57.1)	-1.702 (-1.803 to -1.601)	-0.232 (-5.432 to 5.254)
Andorra	30.1 (21.6 to 39.0)	17.7 (12.5 to 23.1)	-1.783 (-2.063 to -1.502)	-8.053 (-16.300 to 1.007)
Angola	2333.4 (1765.7 to 3063.8)	29.3 (22.6 to 38.2)	-2.030 (-2.128 to -1.931)	0.118 (-0.696 to 0.938)
Antigua and Barbuda	7.2 (6.6 to 7.8)	7.7 (7.1 to 8.5)	0.986 (0.778 to 1.194)	-2.236 (-6.317 to 2.024)
Argentina	14312.4 (13094.5 to 15431.0)	24.5 (22.4 to 26.4)	-0.244 (-0.564 to 0.076)	-5.741 (-7.716 to -3.724)
Armenia	658.7 (568.1 to 752.3)	15.2 (13.1 to 17.3)	-3.027 (-3.661 to -2.389)	-0.981 (-1.362 to -0.598)
Australia	9482.6 (8304.2 to 10312.1)	18.3 (16.2 to 19.9)	-1.539 (-1.803 to -1.275)	-1.851 (-7.262 to 3.875)
Austria	3268.6 (2870.2 to 3515.7)	15.7 (13.9 to 16.8)	0.197 (-0.024 to 0.419)	-3.297 (-5.979 to -0.539)
Azerbaijan	1217.7 (957.0 to 1659.4)	15.3 (12.1 to 19.8)	-2.355 (-2.679 to -2.030)	-3.531 (-4.386 to -2.669)
Bahamas	36.3 (29.6 to 43.8)	10.3 (8.5 to 12.4)	0.310 (0.172 to 0.449)	-0.995 (-1.076 to -0.913)
Bahrain	144.7 (121.1 to 168.9)	35.4 (30.1 to 40.5)	-2.446 (-2.891 to -1.998)	1.179 (0.966 to 1.392)
Bangladesh	71034.0 (54260.1 to 92451.1)	59.6 (46.2 to 77.2)	-1.971 (-2.314 to -1.626)	-1.173 (-1.453 to -0.891)
Barbados	39.5 (31.3 to 47.6)	7.5 (6.0 to 9.1)	0.164 (-0.064 to 0.393)	-3.490 (-6.251 to -0.648)

Belarus	1260.9 (1045.5 to 1471.3)	7.7 (6.4 to 9.0)	-7.327 (-7.986 to -6.664)	-0.350 (-0.782 to 0.084)
Belgium	5897.8 (5006.4 to 6383.7)	21.4 (18.5 to 23.0)	-1.680 (-1.830 to -1.529)	-2.916 (-5.027 to -0.759)
Belize	53.2 (45.8 to 60.5)	20.6 (17.7 to 23.5)	1.092 (0.474 to 1.713)	-3.259 (-7.102 to 0.744)
Benin	981.5 (741.9 to 1308.0)	24.1 (18.3 to 31.8)	-1.117 (-1.340 to -0.894)	-0.506 (-1.046 to 0.037)
Bermuda	13.4 (11.4 to 16.0)	8.7 (7.4 to 10.4)	-1.220 (-1.455 to -0.985)	-0.244 (-5.478 to 5.279)
Bhutan	464.7 (354.1 to 614.4)	87.6 (67.1 to 116.4)	-0.977 (-1.091 to -0.863)	-0.968 (-1.098 to -0.837)
Bolivia (Plurinational State of)	1807.3 (1381.9 to 2333.5)	26.3 (20.4 to 33.4)	-0.781 (-0.838 to -0.724)	-2.628 (-2.840 to -2.415)
Bosnia and Herzegovina	1057.2 (837.8 to 1279.7)	16.3 (12.9 to 19.8)	-2.419 (-2.669 to -2.168)	-2.878 (-6.439 to 0.819)
Botswana	387.7 (309.0 to 509.3)	35.2 (28.0 to 47.7)	-2.025 (-2.173 to -1.877)	-1.555 (-2.877 to -0.216)
Brazil	63674.0 (56870.9 to 67660.4)	26.5 (23.6 to 28.2)	-2.148 (-2.399 to -1.897)	-2.061 (-3.354 to -0.750)
Brunei Darussalam	68.8 (59.1 to 81.0)	33.6 (28.5 to 39.8)	-1.588 (-1.836 to -1.341)	-7.093 (-13.743 to 0.069)
Bulgaria	2240.7 (1907.5 to 2605.2)	15.2 (13.0 to 17.7)	-2.441 (-2.623 to -2.259)	-0.871 (-1.671 to -0.065)
Burkina Faso	1340.7 (1056.7 to 1678.1)	18.5 (15.0 to 23.7)	-0.596 (-0.667 to -0.524)	-1.132 (-1.569 to -0.692)
Burundi	1343.2 (921.0 to 1739.9)	37.7 (26.3 to 49.0)	-1.875 (-2.087 to -1.663)	0.237 (-0.122 to 0.597)
Cabo Verde	66.2 (49.8 to 81.1)	16.2 (12.3 to 19.9)	-2.623 (-3.376 to -1.864)	-1.911 (-1.936 to -1.885)
Cambodia	4071.4 (3184.3 to 5023.1)	46.1 (36.7 to 55.7)	-0.467 (-0.512 to -0.421)	-0.459 (-0.723 to -0.194)
Cameroon	2384.8 (1842.3 to 3129.1)	24.8 (19.6 to 32.2)	-1.255 (-1.391 to -1.118)	-1.722 (-1.919 to -1.524)
Canada	15147.2 (13099.2 to	18.5 (16.1 to 19.9)	-1.075 (-1.209 to -0.941)	-3.550 (-5.610 to -1.444)

	16369.3)			
Central African Republic	844.8 (525.3 to 1222.7)	55.9 (32.8 to 82.3)	-0.621 (-0.666 to -0.576)	-1.518 (-2.010 to -1.024)
Chad	1389.0 (976.8 to 1793.7)	30.8 (21.9 to 39.4)	-0.116 (-0.217 to -0.015)	-0.129 (-0.386 to 0.129)
Chile	4234.9 (3683.1 to 4567.1)	16.0 (13.9 to 17.2)	-0.832 (-1.215 to -0.447)	-4.629 (-8.487 to -0.609)
China	1285433.2 (1044727.8 to 1539819.9)	73.2 (59.7 to 86.9)	-4.250 (-4.473 to -4.026)	-0.942 (-1.050 to -0.835)
Colombia	17399.1 (14352.8 to 20325.2)	30.4 (25.2 to 35.4)	-1.140 (-1.327 to -0.954)	1.372 (0.643 to 2.107)
Comoros	103.1 (70.0 to 136.7)	26.3 (18.2 to 34.8)	-1.773 (-1.997 to -1.548)	0.213 (-0.000 to 0.427)
Congo	674.3 (510.7 to 886.1)	36.2 (28.3 to 47.3)	-1.835 (-1.939 to -1.730)	-1.038 (-1.175 to -0.901)
Cook Islands	6.1 (4.9 to 7.5)	24.8 (20.1 to 30.7)	-2.761 (-2.935 to -2.586)	-1.121 (-1.254 to -0.988)
Costa Rica	1120.8 (936.1 to 1259.0)	19.7 (16.6 to 22.1)	-1.191 (-1.712 to -0.667)	-3.267 (-7.601 to 1.270)
Croatia	1947.1 (1708.2 to 2177.5)	19.1 (16.7 to 21.2)	0.158 (0.027 to 0.288)	-0.393 (-0.597 to -0.188)
Cuba	4669.7 (4087.5 to 5257.0)	22.6 (19.8 to 25.4)	1.168 (0.990 to 1.347)	-0.913 (-1.133 to -0.692)
Cyprus	460.2 (382.8 to 539.9)	27.0 (22.7 to 31.6)	-2.693 (-2.977 to -2.407)	-4.139 (-6.354 to -1.873)
Czechia	3903.4 (3355.4 to 4406.6)	16.6 (14.2 to 18.8)	1.040 (0.408 to 1.676)	-1.230 (-2.698 to 0.260)
C 么 te d'Ivoire	2003.8 (1563.3 to 2568.0)	24.4 (19.6 to 30.8)	-1.160 (-1.301 to -1.018)	-1.240 (-1.329 to -1.152)
Democratic People's Republic of Korea	30211.2 (22332.5 to 42248.3)	107.7 (80.7 to 156.7)	-1.227 (-1.337 to -1.118)	-1.704 (-1.721 to -1.687)
Democratic Republic of the Congo	12256.8 (7545.3 to 19734.6)	47.5 (29.6 to 79.6)	-0.303 (-0.348 to -0.259)	0.230 (-0.200 to 0.662)

Denmark	4822.0 (4240.6 to 5197.0)	35.4 (31.4 to 38.1)	-0.205 (-0.460 to 0.051)	-1.298 (-3.045 to 0.481)
Djibouti	87.7 (55.2 to 127.2)	20.5 (13.0 to 29.2)	-1.519 (-1.674 to -1.363)	-0.965 (-1.108 to -0.821)
Dominica	13.9 (11.9 to 16.5)	17.9 (15.3 to 21.0)	-0.022 (-0.054 to 0.009)	-0.153 (-0.325 to 0.018)
Dominican Republic	1253.5 (911.1 to 1920.5)	13.1 (9.5 to 20.0)	0.163 (-0.099 to 0.426)	1.078 (-4.997 to 7.542)
Ecuador	2425.0 (2005.5 to 2893.9)	17.4 (14.6 to 20.6)	-0.679 (-0.943 to -0.415)	-4.557 (-8.620 to -0.313)
Egypt	10701.8 (8682.5 to 13028.3)	25.9 (21.5 to 31.1)	-2.122 (-2.314 to -1.930)	-0.865 (-1.010 to -0.720)
El Salvador	1228.4 (962.1 to 1565.5)	17.7 (13.7 to 22.6)	-0.983 (-1.138 to -0.828)	-0.614 (-2.363 to 1.167)
Equatorial Guinea	91.1 (61.3 to 133.5)	25.4 (18.0 to 36.8)	-2.937 (-3.203 to -2.670)	-0.871 (-0.973 to -0.769)
Eritrea	707.0 (535.2 to 913.9)	36.5 (28.0 to 46.8)	-1.100 (-1.180 to -1.020)	-0.444 (-0.891 to 0.005)
Estonia	192.6 (168.1 to 216.8)	6.1 (5.3 to 6.9)	-1.494 (-1.688 to -1.300)	-0.189 (-0.565 to 0.188)
Eswatini	237.5 (173.4 to 314.4)	52.4 (38.5 to 68.7)	-0.401 (-0.828 to 0.028)	-6.532 (-8.854 to -4.150)
Ethiopia	8817.4 (6883.1 to 10509.5)	25.4 (20.3 to 30.1)	-2.182 (-2.309 to -2.055)	-0.264 (-0.553 to 0.025)
Fiji	200.7 (149.2 to 250.7)	38.5 (29.3 to 47.6)	-2.104 (-2.405 to -1.802)	-1.264 (-1.553 to -0.974)
Finland	1503.7 (1311.3 to 1641.0)	9.9 (8.7 to 10.7)	-0.731 (-0.895 to -0.565)	-1.559 (-2.574 to -0.534)
France	16593.4 (14087.9 to 18170.9)	8.9 (7.7 to 9.7)	-2.757 (-3.194 to -2.318)	-2.544 (-3.568 to -1.509)
Gabon	204.2 (146.8 to 292.3)	26.2 (19.1 to 37.9)	-1.847 (-1.919 to -1.776)	-1.335 (-1.818 to -0.850)
Gambia	244.0 (168.5 to 330.8)	30.7 (21.1 to 41.5)	-0.309 (-0.565 to -0.052)	-0.583 (-0.954 to -0.210)
Georgia	700.6 (597.2 to 812.8)	11.0 (9.4 to 12.7)	2.841 (2.129 to 3.557)	-0.114 (-5.158 to 5.199)
Germany	40495.9 (35692.3 to 43610.1)	18.3 (16.5 to 19.5)	-0.230 (-0.518 to 0.059)	-1.920 (-2.140 to -1.699)

Ghana	1781.8 (1402.3 to 2230.5)	13.8 (11.0 to 17.1)	0.196 (-0.096 to 0.490)	-1.458 (-2.000 to -0.912)
Greece	4996.1 (4291.5 to 5422.8)	15.0 (13.1 to 16.1)	0.704 (-0.058 to 1.471)	-0.062 (-0.420 to 0.297)
Greenland	23.8 (19.7 to 28.6)	43.0 (34.5 to 52.8)	-2.144 (-2.269 to -2.019)	-3.586 (-10.036 to 3.327)
Grenada	13.9 (12.1 to 15.5)	13.6 (11.9 to 15.1)	0.214 (-0.358 to 0.790)	-0.555 (-1.717 to 0.620)
Guam	29.8 (24.9 to 34.2)	13.6 (11.6 to 15.6)	-3.443 (-3.779 to -3.105)	-10.089 (-22.881 to 4.826)
Guatemala	1601.1 (1383.4 to 1813.3)	17.8 (15.6 to 20.0)	-1.416 (-1.682 to -1.151)	-8.310 (-16.600 to 0.805)
Guinea	1335.9 (985.4 to 1759.2)	28.7 (21.2 to 37.2)	-0.258 (-0.429 to -0.087)	-0.972 (-0.989 to -0.956)
Guinea-Bissau	195.0 (133.6 to 249.5)	36.1 (24.4 to 45.9)	-0.776 (-1.005 to -0.546)	-1.832 (-2.180 to -1.483)
Guyana	78.2 (61.0 to 99.3)	14.2 (11.2 to 17.7)	0.745 (0.471 to 1.020)	-1.607 (-2.298 to -0.911)
Haiti	1889.3 (835.0 to 2864.0)	35.2 (16.1 to 53.1)	-0.197 (-0.267 to -0.126)	-1.120 (-1.478 to -0.761)
Honduras	2767.1 (2171.5 to 3486.7)	56.6 (44.8 to 70.3)	0.963 (0.734 to 1.192)	-1.914 (-1.956 to -1.871)
Hungary	5990.3 (5208.4 to 6792.9)	29.2 (25.6 to 33.3)	-0.151 (-0.617 to 0.316)	-2.157 (-2.988 to -1.320)
Iceland	113.9 (93.6 to 127.4)	16.9 (14.1 to 18.8)	-0.292 (-0.460 to -0.124)	-3.877 (-8.277 to 0.735)
India	1066181.2 (939670.3 to 1202912.4)	108.4 (94.7 to 122.4)	-0.096 (-0.287 to 0.096)	-2.651 (-2.753 to -2.548)
Indonesia	85259.5 (69822.7 to 101680.4)	49.5 (40.9 to 58.2)	-0.186 (-0.310 to -0.063)	-0.232 (-0.367 to -0.096)
Iran (Islamic Republic of)	11440.4 (9949.5 to 12568.6)	17.4 (15.1 to 19.2)	-0.317 (-0.436 to -0.197)	-3.644 (-6.137 to -1.084)
Iraq	2006.3 (1504.8 to 2512.3)	11.4 (8.7 to 14.4)	-0.744 (-0.952 to -0.536)	5.189 (-1.978 to 12.881)

Ireland	2003.6 (1720.2 to 2215.5)	23.3 (20.0 to 25.7)	-2.679 (-2.967 to -2.391)	-7.059 (-7.691 to -6.423)
Israel	1665.7 (1408.0 to 1821.5)	12.1 (10.3 to 13.2)	-1.842 (-2.066 to -1.617)	-3.309 (-4.149 to -2.461)
Italy	28602.4 (23503.7 to 31322.3)	14.2 (11.9 to 15.4)	-1.311 (-1.486 to -1.136)	-0.874 (-3.237 to 1.547)
Jamaica	473.5 (369.7 to 592.8)	14.7 (11.5 to 18.5)	0.361 (-0.198 to 0.924)	-0.582 (-4.094 to 3.059)
Japan	32777.0 (26625.9 to 35933.5)	5.8 (4.9 to 6.3)	-2.464 (-2.751 to -2.176)	-0.508 (-3.186 to 2.244)
Jordan	562.2 (443.1 to 696.2)	10.7 (8.6 to 13.2)	-2.743 (-3.042 to -2.443)	1.218 (0.402 to 2.040)
Kazakhstan	7144.9 (6148.2 to 8309.0)	46.7 (40.3 to 54.1)	-0.258 (-0.910 to 0.399)	-1.364 (-1.804 to -0.922)
Kenya	5823.5 (3524.1 to 10425.9)	34.6 (20.7 to 64.8)	0.397 (0.273 to 0.522)	-0.971 (-1.261 to -0.680)
Kiribati	39.6 (28.7 to 62.4)	83.3 (62.5 to 133.8)	-0.884 (-0.959 to -0.808)	-0.633 (-0.849 to -0.417)
Kuwait	56.6 (46.4 to 67.7)	2.7 (2.2 to 3.3)	-2.487 (-3.010 to -1.961)	0.412 (-7.073 to 8.501)
Kyrgyzstan	1066.5 (896.5 to 1237.6)	28.2 (23.9 to 32.7)	-4.523 (-5.056 to -3.987)	-1.767 (-2.245 to -1.287)
Lao People's Democratic Republic	1945.7 (1402.4 to 2564.4)	56.4 (41.3 to 72.8)	-1.636 (-1.731 to -1.540)	-0.793 (-0.901 to -0.685)
Latvia	299.4 (254.7 to 348.5)	6.9 (5.9 to 8.1)	-1.826 (-2.215 to -1.435)	-0.478 (-2.827 to 1.928)
Lebanon	1309.4 (1074.8 to 1565.0)	19.6 (16.2 to 23.5)	-1.187 (-1.353 to -1.020)	-0.585 (-0.649 to -0.521)
Lesotho	618.2 (431.5 to 818.2)	68.0 (48.5 to 90.0)	1.012 (0.668 to 1.357)	-4.372 (-7.323 to -1.328)
Liberia	420.7 (311.0 to 576.8)	26.6 (19.7 to 35.9)	-0.785 (-0.975 to -0.595)	0.140 (0.061 to 0.219)
Libya	851.0 (649.4 to 1144.3)	20.0 (15.4 to 26.6)	0.609 (0.388 to 0.830)	-0.795 (-1.155 to -0.435)
Lithuania	569.9 (505.9 to 629.4)	8.9 (7.9 to 9.8)	-3.944 (-4.114 to -3.773)	-0.807 (-1.549 to -0.059)

Luxembourg	222.0 (194.7 to 248.8)	18.6 (16.4 to 20.8)	-1.010 (-1.144 to -0.876)	-4.577 (-5.801 to -3.337)
Madagascar	3940.5 (2948.3 to 5196.8)	54.0 (40.8 to 70.8)	-0.538 (-0.616 to -0.459)	0.021 (0.009 to 0.034)
Malawi	1684.0 (1366.7 to 2085.8)	29.2 (23.9 to 35.6)	-0.604 (-0.776 to -0.432)	-1.080 (-2.091 to -0.059)
Malaysia	6779.6 (5913.1 to 7635.4)	28.8 (24.9 to 32.9)	-1.838 (-2.156 to -1.519)	0.512 (-15.940 to 20.183)
Maldives	89.6 (73.6 to 108.5)	35.0 (29.1 to 42.5)	-3.527 (-3.653 to -3.400)	-1.992 (-6.026 to 2.214)
Mali	3091.5 (2369.8 to 4071.2)	44.4 (35.4 to 57.1)	-0.184 (-0.304 to -0.064)	-1.558 (-2.082 to -1.032)
Malta	110.3 (94.9 to 123.0)	9.7 (8.5 to 10.9)	-2.672 (-2.902 to -2.443)	-3.630 (-10.224 to 3.449)
Marshall Islands	15.7 (11.7 to 20.7)	71.3 (53.5 to 90.5)	-1.095 (-1.172 to -1.019)	-1.245 (-1.299 to -1.191)
Mauritania	337.4 (258.2 to 430.6)	19.3 (14.8 to 24.4)	-1.629 (-1.999 to -1.257)	0.781 (-0.145 to 1.716)
Mauritius	276.6 (252.7 to 293.9)	16.9 (15.3 to 18.0)	-2.352 (-2.621 to -2.081)	0.395 (-5.066 to 6.170)
Mexico	30707.0 (27403.7 to 33861.9)	27.9 (24.9 to 30.7)	-1.462 (-1.588 to -1.335)	-0.935 (-3.371 to 1.562)
Micronesia (Federated States of)	33.9 (26.3 to 44.4)	67.1 (53.3 to 86.2)	-1.889 (-1.992 to -1.785)	-0.746 (-0.919 to -0.572)
Monaco	14.7 (11.3 to 18.2)	12.1 (9.5 to 14.9)	-0.292 (-0.377 to -0.206)	-0.870 (-1.438 to -0.300)
Mongolia	336.9 (268.1 to 406.9)	20.6 (16.4 to 24.9)	-2.954 (-3.214 to -2.693)	-1.646 (-4.642 to 1.445)
Montenegro	52.9 (41.7 to 64.2)	5.9 (4.6 to 7.1)	0.165 (-0.120 to 0.452)	-0.920 (-7.647 to 6.297)
Morocco	6708.8 (5244.7 to 8061.9)	23.5 (18.5 to 28.3)	0.496 (0.382 to 0.611)	-0.588 (-0.970 to -0.204)
Mozambique	2337.7 (1776.2 to 2918.6)	27.8 (21.0 to 35.0)	0.248 (0.105 to 0.391)	-2.493 (-3.427 to -1.551)
Myanmar	40096.1 (31826.5 to	104.5 (82.5 to 126.4)	-1.110 (-1.217 to -1.003)	-0.057 (-0.336 to 0.224)

	48951.3)			
Namibia	559.9 (439.9 to 731.4)	51.4 (40.8 to 68.2)	-0.888 (-1.093 to -0.683)	-2.280 (-3.388 to -1.159)
Nauru	3.1 (2.2 to 4.9)	79.3 (53.8 to 140.8)	-1.144 (-1.271 to -1.017)	-0.804 (-0.922 to -0.685)
Nepal	27536.4 (21511.8 to 34735.8)	146.1 (116.7 to 182.5)	-0.469 (-0.693 to -0.244)	-0.343 (-0.729 to 0.044)
Netherlands	10731.2 (9299.3 to 11612.4)	26.9 (23.5 to 29.1)	-0.816 (-0.965 to -0.666)	-0.341 (-0.438 to -0.243)
New Zealand	2032.3 (1765.7 to 2202.4)	22.0 (19.2 to 23.8)	-1.653 (-1.846 to -1.460)	-1.998 (-6.470 to 2.688)
Nicaragua	749.0 (618.5 to 889.1)	18.7 (15.2 to 22.1)	0.771 (0.387 to 1.157)	-5.743 (-8.988 to -2.382)
Niger	1700.6 (1163.4 to 2280.2)	28.5 (19.0 to 37.5)	-0.527 (-0.754 to -0.299)	-0.508 (-0.547 to -0.468)
Nigeria	12570.5 (10544.6 to 15344.3)	18.6 (15.8 to 22.1)	-0.795 (-0.866 to -0.724)	-1.264 (-1.753 to -0.771)
Niue	1.0 (0.8 to 1.2)	48.5 (39.2 to 59.6)	-1.634 (-1.718 to -1.551)	-0.172 (-0.859 to 0.518)
North Macedonia	559.1 (415.9 to 785.3)	21.0 (16.0 to 28.3)	-1.146 (-1.388 to -0.904)	-4.373 (-6.026 to -2.691)
Northern Mariana Islands	16.3 (14.3 to 18.7)	47.0 (40.8 to 53.5)	-1.288 (-1.414 to -1.162)	0.662 (-17.517 to 22.847)
Norway	2843.9 (2475.0 to 3042.4)	24.7 (21.7 to 26.3)	2.222 (1.766 to 2.681)	-1.410 (-4.250 to 1.513)
Oman	182.8 (147.5 to 236.7)	14.2 (11.6 to 17.6)	-0.257 (-0.613 to 0.100)	-3.714 (-12.857 to 6.389)
Pakistan	64393.1 (51864.2 to 81021.5)	73.8 (60.0 to 94.6)	-0.664 (-0.912 to -0.415)	-1.370 (-1.516 to -1.225)
Palau	9.9 (8.0 to 12.1)	70.5 (58.0 to 84.9)	-0.373 (-0.462 to -0.285)	-0.089 (-0.600 to 0.424)
Palestine	287.5 (243.1 to 348.2)	16.3 (13.9 to 19.9)	-1.947 (-2.249 to -1.645)	0.763 (-1.798 to 3.391)
Panama	717.1 (557.1 to 856.3)	15.5 (12.1 to 18.6)	-0.824 (-1.183 to -0.463)	-2.320 (-3.089 to -1.545)
Papua New Guinea	5186.4 (4049.0 to	156.8 (123.6 to 197.4)	-0.579 (-0.618 to -0.540)	-0.699 (-1.407 to 0.014)

	6549.7)			
Paraguay	988.1 (787.5 to 1243.0)	18.7 (14.9 to 23.5)	0.652 (0.494 to 0.810)	-5.847 (-9.194 to -2.375)
Peru	3158.1 (2190.0 to 4277.5)	9.4 (6.5 to 12.9)	-0.860 (-1.032 to -0.687)	-5.994 (-7.828 to -4.124)
Philippines	21865.7 (18313.7 to 25798.6)	32.8 (27.7 to 38.2)	-0.924 (-1.007 to -0.842)	-1.348 (-2.006 to -0.685)
Poland	9126.2 (8251.3 to 9904.6)	11.8 (10.7 to 12.8)	-2.194 (-2.344 to -2.043)	-0.086 (-0.165 to -0.006)
Portugal	5702.9 (4769.4 to 6270.6)	17.6 (14.9 to 19.2)	-1.732 (-2.037 to -1.427)	-1.953 (-2.015 to -1.890)
Puerto Rico	1553.6 (1258.1 to 1821.6)	17.0 (14.0 to 19.8)	-1.756 (-2.111 to -1.400)	-0.880 (-1.951 to 0.202)
Qatar	50.9 (39.5 to 65.1)	12.8 (10.0 to 15.9)	-3.263 (-3.914 to -2.607)	7.839 (-0.808 to 17.240)
Republic of Korea	10916.7 (8858.9 to 13478.6)	12.0 (9.7 to 14.8)	-3.076 (-3.337 to -2.815)	0.675 (-1.436 to 2.831)
Republic of Moldova	695.0 (615.5 to 780.1)	11.5 (10.3 to 13.0)	-4.994 (-5.511 to -4.475)	-0.685 (-1.913 to 0.558)
Romania	6865.4 (6056.7 to 7638.6)	17.4 (15.3 to 19.4)	-3.689 (-3.999 to -3.379)	-0.282 (-1.332 to 0.779)
Russian Federation	31664.2 (29115.3 to 34367.9)	13.0 (12.0 to 14.1)	-3.232 (-3.585 to -2.878)	-1.248 (-2.124 to -0.365)
Rwanda	1602.7 (1091.7 to 2165.8)	34.5 (24.0 to 46.3)	-2.897 (-3.238 to -2.555)	0.703 (0.418 to 0.989)
Saint Kitts and Nevis	9.0 (7.6 to 10.3)	16.6 (14.2 to 18.6)	0.524 (0.326 to 0.722)	-0.243 (-0.646 to 0.162)
Saint Lucia	54.3 (44.2 to 64.2)	23.5 (19.2 to 27.8)	-0.897 (-1.217 to -0.577)	0.181 (0.119 to 0.243)
Saint Vincent and the Grenadines	14.5 (12.8 to 16.3)	11.2 (9.9 to 12.5)	1.408 (1.080 to 1.737)	0.316 (-0.277 to 0.912)

Samoa	80.7 (63.6 to 99.5)	69.6 (54.9 to 86.0)	-1.264 (-1.346 to -1.181)	-0.987 (-1.282 to -0.692)
San Marino	6.2 (4.3 to 8.4)	6.0 (4.3 to 8.3)	-1.354 (-1.734 to -0.973)	-21.922 (-37.871 to -1.878)
Sao Tome and Principe	50.7 (38.3 to 63.9)	58.4 (44.2 to 72.2)	-0.149 (-0.277 to -0.021)	-1.798 (-1.894 to -1.703)
Saudi Arabia	2803.9 (2266.2 to 3415.3)	22.7 (18.6 to 27.3)	-1.448 (-1.522 to -1.375)	-1.197 (-1.280 to -1.114)
Senegal	1612.8 (1235.9 to 2068.2)	25.9 (20.1 to 33.0)	-0.538 (-0.933 to -0.142)	-0.601 (-0.654 to -0.548)
Serbia	3456.4 (2903.9 to 4065.4)	19.8 (16.6 to 23.3)	-1.838 (-1.954 to -1.722)	-0.828 (-4.058 to 2.510)
Seychelles	23.1 (19.9 to 26.7)	23.7 (20.4 to 27.5)	-1.311 (-1.454 to -1.168)	-5.673 (-18.986 to 9.828)
Sierra Leone	774.6 (549.9 to 1057.0)	25.6 (18.4 to 34.6)	-0.593 (-0.767 to -0.418)	-0.661 (-0.863 to -0.458)
Singapore	488.4 (427.8 to 526.3)	5.9 (5.2 to 6.4)	-6.292 (-6.409 to -6.175)	-2.411 (-4.026 to -0.768)
Slovakia	934.2 (784.4 to 1119.9)	9.6 (8.1 to 11.5)	-0.690 (-0.838 to -0.541)	-0.319 (-1.876 to 1.263)
Slovenia	608.8 (522.4 to 671.6)	11.5 (9.9 to 12.7)	-3.339 (-3.669 to -3.007)	-2.042 (-4.539 to 0.520)
Solomon Islands	156.0 (125.9 to 194.0)	66.5 (54.6 to 80.6)	-0.931 (-0.988 to -0.874)	-0.733 (-1.400 to -0.061)
Somalia	1848.7 (1097.1 to 2603.0)	41.4 (23.9 to 57.9)	-1.078 (-1.142 to -1.014)	-2.745 (-3.731 to -1.749)
South Africa	13322.0 (12247.3 to 14499.7)	34.0 (31.2 to 36.9)	-0.479 (-0.880 to -0.076)	-0.745 (-1.284 to -0.204)
South Sudan	1069.3 (712.8 to 1493.4)	37.7 (25.3 to 52.0)	-0.933 (-1.146 to -0.718)	-0.514 (-1.443 to 0.424)
Spain	29647.5 (24925.8 to 32607.9)	22.0 (19.0 to 24.0)	-1.642 (-1.813 to -1.471)	-3.099 (-5.710 to -0.415)
Sri Lanka	10679.1 (7286.3 to 13972.1)	46.2 (31.8 to 60.7)	-1.158 (-1.412 to -0.903)	-2.941 (-13.393 to 8.772)
Sudan	4013.2 (2540.9 to 5759.7)	25.5 (16.3 to 36.4)	-1.084 (-1.128 to -1.041)	-0.001 (-0.371 to 0.371)

Suriname	90.7 (69.9 to 115.6)	15.3 (11.9 to 19.7)	-0.572 (-0.775 to -0.369)	-6.730 (-10.538 to -2.760)
Sweden	3601.1 (3104.8 to 3987.8)	13.5 (11.7 to 14.9)	0.954 (0.635 to 1.273)	-3.586 (-4.340 to -2.827)
Switzerland	2496.4 (2092.4 to 2760.7)	11.3 (9.7 to 12.4)	-1.487 (-1.582 to -1.392)	-2.129 (-2.979 to -1.273)
Syrian Arab Republic	2485.5 (1897.5 to 3161.2)	25.8 (20.4 to 31.7)	-0.211 (-0.343 to -0.079)	-0.464 (-0.706 to -0.221)
Taiwan (Province of China)	7796.6 (6743.7 to 8582.3)	17.1 (14.9 to 18.7)	-2.051 (-2.293 to -1.808)	-2.055 (-5.704 to 1.735)
Tajikistan	1225.5 (889.1 to 1656.8)	29.1 (20.8 to 38.9)	-1.996 (-2.283 to -1.708)	0.316 (-0.417 to 1.055)
Thailand	21391.9 (16484.0 to 27003.5)	19.8 (15.2 to 25.0)	-4.052 (-4.344 to -3.760)	1.394 (0.977 to 1.813)
Timor-Leste	353.9 (262.4 to 451.6)	51.4 (38.5 to 65.4)	-0.506 (-0.611 to -0.400)	-0.849 (-1.287 to -0.410)
Togo	793.4 (584.2 to 1074.3)	28.2 (20.9 to 38.1)	-0.492 (-0.657 to -0.327)	-0.087 (-0.409 to 0.235)
Tokelau	0.7 (0.5 to 1.0)	45.4 (33.1 to 66.2)	-1.517 (-1.580 to -1.454)	-0.335 (-0.721 to 0.053)
Tonga	36.6 (29.8 to 44.1)	49.0 (39.8 to 58.7)	-0.869 (-1.002 to -0.736)	-0.915 (-1.229 to -0.600)
Trinidad and Tobago	202.9 (156.9 to 251.1)	10.8 (8.3 to 13.3)	-1.099 (-1.266 to -0.933)	-0.024 (-1.260 to 1.227)
Tunisia	2021.5 (1407.8 to 2900.0)	17.6 (12.2 to 26.0)	-0.342 (-0.435 to -0.250)	-0.634 (-1.085 to -0.182)
Turkmenistan	385.5 (301.2 to 486.5)	11.0 (8.8 to 13.7)	-4.915 (-5.633 to -4.190)	-0.189 (-0.353 to -0.025)
Tuvalu	4.5 (3.4 to 5.8)	54.9 (41.5 to 70.2)	-1.974 (-2.052 to -1.895)	-1.289 (-1.330 to -1.247)
T 眉 rkiye	34044.5 (28218.9 to 40618.5)	40.8 (33.9 to 48.5)	-0.720 (-1.094 to -0.345)	-1.431 (-3.804 to 1.000)
Uganda	3277.8 (2197.5 to 4445.5)	28.9 (19.6 to 39.0)	-1.866 (-2.020 to -1.711)	-0.664 (-1.284 to -0.040)
Ukraine	7471.1 (5654.2 to	9.4 (7.1 to 11.8)	-6.783 (-7.290 to -6.273)	-2.948 (-6.822 to 1.088)

	9388.6)			
United Arab Emirates	428.2 (338.6 to 532.8)	26.3 (18.3 to 33.5)	0.603 (-0.057 to 1.267)	-17.694 (-30.015 to -3.203)
United Kingdom	39383.3 (34801.3 to 41582.5)	26.2 (23.4 to 27.6)	-0.606 (-0.733 to -0.478)	-2.901 (-4.844 to -0.918)
United Republic of Tanzania	4389.7 (3347.6 to 5593.3)	21.6 (16.8 to 27.4)	-1.239 (-1.316 to -1.162)	-0.143 (-0.426 to 0.142)
United States Virgin Islands	13.7 (10.5 to 18.2)	7.7 (5.9 to 10.2)	-1.533 (-1.746 to -1.319)	-8.191 (-15.689 to -0.027)
United States of America	198036.5 (172625.1 to 210192.2)	31.3 (27.5 to 33.1)	0.479 (0.268 to 0.689)	0.076 (-1.055 to 1.221)
Uruguay	2070.6 (1891.0 to 2208.2)	32.4 (29.7 to 34.5)	0.099 (-0.071 to 0.269)	-0.644 (-7.576 to 6.807)
Uzbekistan	1735.9 (1475.7 to 2052.9)	8.8 (7.5 to 10.3)	-4.444 (-5.266 to -3.614)	-0.445 (-0.517 to -0.372)
Vanuatu	100.9 (63.2 to 133.2)	83.4 (54.1 to 112.1)	-1.205 (-1.264 to -1.146)	-0.861 (-1.054 to -0.667)
Venezuela (Bolivarian Republic of)	6091.3 (4713.7 to 7724.7)	22.1 (17.2 to 27.8)	0.354 (0.022 to 0.686)	1.584 (1.556 to 1.612)
Viet Nam	35336.5 (27905.0 to 43547.7)	44.0 (35.1 to 54.2)	-0.657 (-0.759 to -0.554)	-1.245 (-1.356 to -1.134)
Yemen	3373.7 (2433.7 to 4462.0)	31.3 (22.7 to 41.3)	-0.654 (-0.739 to -0.570)	0.905 (0.754 to 1.057)
Zambia	1535.1 (1185.1 to 1939.6)	29.4 (23.1 to 36.8)	-0.688 (-0.831 to -0.544)	-2.905 (-4.718 to -1.057)
Zimbabwe	1453.8 (1125.6 to 1826.9)	29.0 (22.4 to 35.8)	0.591 (0.306 to 0.876)	-3.616 (-6.079 to -1.088)

EAPC: Estimated Annual Percentage Change; ASRs: Age-standardized rates; COPD: Chronic Obstructive Pulmonary Disease; EAPCs for ASRs from 1990 to 2019 (%, 95% UI) were calculated using GBD 2021.

Table S3. Age standardised DALY rates, EAPC for ASRs of COPD at national level, 1990-2021 and 2019-2021

location	No, in 2021 (95% UI)	ASRs, per 100,000, in 2021 (95% UI)	EAPC for ASRs from 1990 to 2021 (95% CI)	EAPC for ASRs from 2019 to 2021 (95% CI)
Afghanistan	113688.7 (86374.1 to 144932.2)	1155.2 (875.1 to 1480.1)	-0.842 (-1.051 to -0.632)	-0.401 (-1.159 to 0.364)
Albania	14700.4 (11935.5 to 18668.1)	342.4 (280.7 to 430.4)	-2.553 (-2.876 to -2.229)	-1.079 (-4.533 to 2.500)
Algeria	154501.2 (131187.8 to 180952.8)	488.6 (415.4 to 573.2)	-0.089 (-0.161 to -0.017)	-0.123 (-0.213 to -0.033)
American Samoa	430.5 (371.0 to 500.5)	994.0 (860.8 to 1150.4)	-1.599 (-1.696 to -1.502)	-0.238 (-5.044 to 4.812)
Andorra	618.2 (481.4 to 766.2)	391.0 (303.1 to 487.0)	-1.538 (-1.760 to -1.315)	-6.611 (-13.051 to 0.307)
Angola	75876.1 (60578.6 to 93582.2)	692.5 (547.4 to 863.0)	-1.867 (-1.948 to -1.786)	0.382 (-0.287 to 1.056)
Antigua and Barbuda	181.8 (167.5 to 196.9)	179.3 (165.5 to 194.0)	0.841 (0.700 to 0.981)	-0.750 (-5.801 to 4.570)
Argentina	276792.7 (257234.1 to 294660.1)	487.7 (454.2 to 519.7)	-0.426 (-0.669 to -0.184)	-4.704 (-6.852 to -2.506)
Armenia	14601.2 (13080.2 to 16353.3)	339.9 (305.4 to 380.0)	-3.158 (-3.592 to -2.721)	-0.838 (-1.596 to -0.075)
Australia	174320.9 (157434.8 to 186600.8)	365.8 (332.3 to 390.2)	-1.695 (-1.916 to -1.474)	-2.026 (-5.690 to 1.779)
Austria	70997.9 (64998.0 to 76794.7)	381.1 (350.5 to 411.1)	0.236 (0.096 to 0.377)	-2.929 (-3.733 to -2.118)
Azerbaijan	35021.7 (28521.0 to 47582.8)	374.5 (309.2 to 479.6)	-2.288 (-2.557 to -2.018)	-2.147 (-3.491 to -0.785)
Bahamas	931.9 (776.0 to 1094.2)	239.3 (201.0 to 280.3)	0.249 (0.142 to 0.357)	-0.796 (-1.026 to -0.565)
Bahrain	4624.6 (3987.6 to 5251.8)	681.5 (591.2 to 765.3)	-2.413 (-2.761 to -2.064)	0.771 (0.348 to 1.196)

Bangladesh	1713712.8 (1376728.8 to 2133832.8)	1301.7 (1050.5 to 1612.2)	-1.912 (-2.134 to -1.689)	-0.980 (-1.200 to -0.760)
Barbados	885.3 (731.5 to 1039.5)	171.8 (142.6 to 201.2)	0.248 (0.100 to 0.396)	-2.786 (-4.024 to -1.533)
Belarus	38503.4 (33454.5 to 44553.7)	245.2 (212.9 to 283.0)	-5.737 (-6.264 to -5.206)	-0.004 (-0.202 to 0.194)
Belgium	115161.7 (103934.0 to 123893.7)	477.6 (437.2 to 512.9)	-1.354 (-1.460 to -1.247)	-2.165 (-6.441 to 2.306)
Belize	1286.1 (1136.0 to 1457.5)	444.6 (391.6 to 501.5)	1.007 (0.479 to 1.538)	-2.716 (-5.965 to 0.646)
Benin	31985.5 (26021.1 to 39965.1)	604.1 (487.7 to 760.4)	-0.892 (-1.092 to -0.692)	-0.146 (-0.545 to 0.255)
Bermuda	278.3 (240.6 to 322.8)	198.7 (171.3 to 230.3)	-0.946 (-1.107 to -0.786)	-0.223 (-4.906 to 4.690)
Bhutan	9571.4 (7479.2 to 12335.7)	1678.9 (1317.3 to 2166.0)	-1.296 (-1.414 to -1.178)	-0.922 (-0.973 to -0.872)
Bolivia (Plurinational State of)	37740.3 (30140.7 to 47632.1)	470.8 (377.0 to 590.9)	-1.008 (-1.044 to -0.972)	-1.499 (-1.534 to -1.464)
Bosnia and Herzegovina	25257.6 (21127.2 to 29835.2)	403.7 (338.7 to 474.4)	-2.008 (-2.197 to -1.819)	-2.193 (-4.460 to 0.129)
Botswana	11491.8 (9596.7 to 14337.5)	840.7 (701.0 to 1049.9)	-1.780 (-1.912 to -1.648)	-0.597 (-0.999 to -0.194)
Brazil	1380989.0 (1280462.4 to 1456315.1)	558.7 (517.0 to 589.0)	-1.961 (-2.203 to -1.720)	-0.907 (-1.528 to -0.283)
Brunei Darussalam	1588.9 (1395.7 to 1833.7)	595.8 (519.5 to 686.7)	-1.855 (-2.016 to -1.693)	-4.552 (-8.517 to -0.415)
Bulgaria	54940.8 (47895.7 to 62497.5)	394.5 (344.8 to 447.9)	-1.818 (-1.955 to -1.682)	-0.387 (-0.523 to -0.250)
Burkina Faso	46286.2 (39032.7 to 55062.0)	491.7 (416.0 to 586.8)	-0.397 (-0.465 to -0.329)	-0.720 (-1.050 to -0.389)
Burundi	41563.2 (30723.1 to	877.5 (646.0 to 1091.7)	-1.712 (-1.914 to -1.510)	0.330 (0.115 to 0.546)

	51254.7)			
Cabo Verde	1713.8 (1397.5 to 2049.2)	388.2 (317.3 to 462.5)	-2.214 (-2.807 to -1.617)	-0.938 (-0.949 to -0.927)
Cambodia	102748.7 (83618.2 to 124865.0)	935.2 (771.7 to 1124.8)	-0.635 (-0.674 to -0.595)	-0.397 (-0.644 to -0.150)
Cameroon	80328.0 (65856.8 to 99654.8)	620.1 (506.5 to 770.6)	-0.943 (-1.055 to -0.832)	-1.037 (-1.425 to -0.648)
Canada	299697.4 (274497.0 to 323214.3)	393.2 (360.9 to 423.7)	-0.975 (-1.087 to -0.862)	-3.046 (-5.325 to -0.713)
Central African Republic	27118.9 (18600.1 to 37062.6)	1302.9 (863.9 to 1797.3)	-0.546 (-0.590 to -0.503)	-0.936 (-0.991 to -0.881)
Chad	45369.1 (34999.8 to 55823.3)	763.6 (583.6 to 942.4)	-0.016 (-0.097 to 0.066)	0.162 (-0.002 to 0.326)
Chile	73317.5 (66461.9 to 78458.2)	282.0 (256.1 to 301.7)	-1.290 (-1.574 to -1.006)	-3.688 (-6.561 to -0.726)
China	23640321.0 (19998658.4 to 27921931.2)	1227.7 (1048.4 to 1442.5)	-4.186 (-4.374 to -3.998)	-0.970 (-1.078 to -0.862)
Colombia	312593.0 (266418.6 to 357444.8)	559.5 (477.8 to 640.2)	-1.517 (-1.709 to -1.325)	1.971 (0.731 to 3.225)
Comoros	2883.7 (2133.0 to 3608.7)	615.5 (455.4 to 774.8)	-1.588 (-1.805 to -1.371)	0.469 (0.050 to 0.889)
Congo	20598.9 (16342.1 to 26280.8)	825.2 (673.4 to 1037.2)	-1.701 (-1.808 to -1.593)	-0.557 (-0.797 to -0.317)
Cook Islands	133.5 (112.4 to 157.9)	534.6 (449.7 to 630.7)	-2.441 (-2.586 to -2.296)	-0.933 (-1.050 to -0.815)
Costa Rica	20986.0 (18547.6 to 23194.0)	378.8 (336.4 to 418.4)	-1.210 (-1.628 to -0.790)	-1.428 (-4.585 to 1.833)
Croatia	38588.4 (34659.0 to 42884.6)	410.2 (367.2 to 456.3)	-0.108 (-0.196 to -0.019)	-0.563 (-1.085 to -0.039)

Cuba	98637.5 (87754.1 to 109681.9)	501.1 (445.8 to 556.5)	1.075 (0.891 to 1.258)	-1.350 (-2.016 to -0.680)
Cyprus	8663.6 (7456.2 to 10028.5)	455.1 (393.6 to 521.7)	-2.366 (-2.561 to -2.171)	-3.716 (-5.776 to -1.611)
Czechia	90888.7 (81256.9 to 101422.9)	414.7 (371.2 to 462.5)	0.654 (0.212 to 1.097)	-0.911 (-1.557 to -0.260)
C 么 te d'Ivoire	70462.5 (57967.1 to 86542.6)	617.3 (510.7 to 756.4)	-0.890 (-1.010 to -0.771)	-0.733 (-0.875 to -0.590)
Democratic People's Republic of Korea	602745.7 (459884.8 to 787221.0)	1968.2 (1500.5 to 2612.2)	-1.134 (-1.239 to -1.029)	-1.589 (-1.622 to -1.555)
Democratic Republic of the Congo	363380.8 (246025.2 to 522318.1)	1073.4 (720.3 to 1615.4)	-0.169 (-0.209 to -0.129)	0.284 (0.077 to 0.492)
Denmark	85639.3 (78029.0 to 91949.7)	682.7 (625.9 to 730.0)	-0.844 (-1.073 to -0.615)	-1.626 (-2.876 to -0.360)
Djibouti	3067.5 (2235.4 to 4106.6)	506.4 (362.2 to 680.9)	-1.213 (-1.352 to -1.074)	-0.438 (-0.569 to -0.308)
Dominica	305.7 (265.3 to 359.8)	373.2 (326.9 to 438.9)	0.144 (0.102 to 0.185)	-0.113 (-0.270 to 0.045)
Dominican Republic	29188.0 (22808.2 to 40487.7)	294.9 (229.7 to 409.1)	0.474 (0.323 to 0.625)	0.250 (-4.512 to 5.251)
Ecuador	45176.7 (38805.0 to 52748.1)	296.9 (256.2 to 345.1)	-0.895 (-1.109 to -0.682)	-3.065 (-6.644 to 0.651)
Egypt	340087.3 (288662.4 to 397678.5)	602.2 (511.8 to 700.3)	-1.915 (-2.116 to -1.714)	-0.351 (-0.454 to -0.247)
El Salvador	23560.2 (19181.6 to 28573.6)	361.1 (294.3 to 437.1)	-0.932 (-1.069 to -0.795)	-0.563 (-2.132 to 1.032)
Equatorial Guinea	3021.2 (2260.6 to 4048.4)	614.0 (458.7 to 833.3)	-2.716 (-3.005 to -2.426)	-0.244 (-0.300 to -0.188)
Eritrea	22655.8 (18134.3 to 28099.0)	844.5 (676.2 to 1047.8)	-1.177 (-1.258 to -1.096)	-0.190 (-0.554 to 0.176)

Estonia	4676.3 (4161.7 to 5224.8)	170.1 (151.1 to 190.8)	-1.261 (-1.415 to -1.107)	-1.426 (-1.862 to -0.987)
Eswatini	7013.9 (5354.0 to 9104.7)	1264.8 (979.9 to 1626.1)	-0.211 (-0.649 to 0.230)	-3.896 (-4.481 to -3.307)
Ethiopia	270087.4 (220925.2 to 310041.6)	619.2 (510.0 to 708.8)	-2.176 (-2.306 to -2.046)	0.188 (0.181 to 0.196)
Fiji	5203.4 (4084.0 to 6385.2)	778.6 (613.6 to 940.8)	-2.008 (-2.223 to -1.793)	-0.826 (-0.876 to -0.776)
Finland	34286.2 (31242.6 to 37492.8)	259.8 (237.7 to 283.4)	-0.771 (-0.892 to -0.650)	-0.909 (-1.904 to 0.095)
France	316491.4 (282301.1 to 347195.2)	208.6 (188.8 to 228.6)	-1.871 (-2.207 to -1.535)	-2.010 (-2.512 to -1.506)
Gabon	6009.9 (4669.7 to 7929.7)	616.7 (481.4 to 822.1)	-1.543 (-1.616 to -1.470)	-0.796 (-1.057 to -0.534)
Gambia	7467.0 (5740.2 to 9533.4)	744.5 (564.1 to 962.6)	-0.258 (-0.482 to -0.034)	-0.385 (-0.806 to 0.039)
Georgia	17653.9 (15566.5 to 19938.2)	295.5 (261.4 to 333.9)	2.002 (1.558 to 2.448)	-0.509 (-2.442 to 1.463)
Germany	866758.3 (790703.6 to 929152.3)	442.9 (409.2 to 475.5)	-0.113 (-0.342 to 0.117)	-1.681 (-1.841 to -1.521)
Ghana	71052.4 (59466.2 to 83837.7)	411.8 (344.9 to 486.0)	0.405 (0.158 to 0.652)	-0.891 (-1.410 to -0.370)
Greece	101175.2 (90603.6 to 112074.9)	380.4 (340.3 to 424.2)	0.608 (0.126 to 1.092)	-0.059 (-0.174 to 0.056)
Greenland	590.0 (509.3 to 685.3)	907.6 (779.9 to 1067.9)	-2.020 (-2.127 to -1.914)	-2.956 (-8.363 to 2.771)
Grenada	343.6 (303.6 to 383.3)	310.3 (274.6 to 343.6)	0.284 (-0.105 to 0.674)	-0.459 (-1.137 to 0.223)
Guam	847.2 (749.8 to 943.4)	405.0 (359.5 to 450.3)	-2.251 (-2.482 to -2.020)	-6.141 (-13.225 to 1.522)
Guatemala	35918.2 (31597.9 to 40508.7)	348.6 (307.5 to 392.1)	-1.328 (-1.608 to -1.048)	-4.514 (-9.606 to 0.865)
Guinea	40917.5 (32166.0 to 51029.8)	713.5 (559.0 to 891.5)	-0.075 (-0.217 to 0.067)	-0.467 (-0.541 to -0.394)

Guinea-Bissau	6749.1 (5046.3 to 8375.6)	887.6 (660.4 to 1092.1)	-0.714 (-0.921 to -0.507)	-1.159 (-1.277 to -1.040)
Guyana	2116.0 (1707.0 to 2641.7)	336.0 (271.5 to 415.6)	0.996 (0.788 to 1.204)	-0.950 (-1.232 to -0.667)
Haiti	48350.7 (23390.0 to 72580.2)	728.1 (356.2 to 1071.2)	-0.254 (-0.310 to -0.198)	-0.736 (-0.785 to -0.687)
Honduras	60342.9 (48626.4 to 74461.2)	1049.6 (846.1 to 1295.3)	0.767 (0.590 to 0.944)	-1.464 (-1.655 to -1.272)
Hungary	140484.5 (124620.1 to 157381.1)	744.2 (658.5 to 834.3)	0.168 (-0.193 to 0.530)	-1.371 (-1.723 to -1.018)
Iceland	2409.6 (2132.0 to 2661.8)	397.5 (353.6 to 437.7)	-0.571 (-0.738 to -0.403)	-2.019 (-4.417 to 0.439)
India	24018380.3 (21633268.2 to 26798624.0)	2171.2 (1953.7 to 2422.4)	-0.360 (-0.463 to -0.257)	-2.301 (-2.526 to -2.076)
Indonesia	2235813.9 (1869853.3 to 2637091.6)	1040.4 (874.7 to 1216.0)	-0.252 (-0.343 to -0.161)	-0.201 (-0.383 to -0.019)
Iran (Islamic Republic of)	314576.6 (285133.0 to 342208.9)	424.1 (383.2 to 462.2)	-0.171 (-0.265 to -0.076)	-2.766 (-4.953 to -0.528)
Iraq	73090.6 (60324.2 to 86600.3)	320.7 (262.9 to 381.1)	-0.526 (-0.638 to -0.414)	2.653 (-1.140 to 6.592)
Ireland	37059.9 (33220.9 to 40422.1)	451.7 (406.9 to 492.3)	-2.621 (-2.865 to -2.376)	-5.513 (-5.616 to -5.411)
Israel	37085.7 (33148.8 to 40842.3)	292.4 (263.8 to 320.7)	-1.293 (-1.453 to -1.132)	-2.682 (-3.448 to -1.911)
Italy	481343.5 (420946.8 to 524958.3)	285.4 (256.1 to 309.7)	-1.334 (-1.476 to -1.191)	-0.849 (-1.558 to -0.135)
Jamaica	10489.2 (8496.0 to 12940.7)	335.4 (271.3 to 413.5)	0.309 (-0.194 to 0.813)	-0.378 (-3.190 to 2.516)
Japan	678591.7 (590707.7 to	155.8 (137.6 to 174.3)	-1.784 (-1.929 to -1.639)	-0.609 (-1.446 to 0.235)

	756041.4)			
Jordan	22173.9 (19008.8 to 25637.8)	311.0 (266.4 to 360.8)	-2.030 (-2.246 to -1.813)	0.793 (0.214 to 1.375)
Kazakhstan	176122.3 (153267.8 to 202154.3)	1023.1 (889.0 to 1171.6)	-0.430 (-0.993 to 0.136)	-1.393 (-3.345 to 0.599)
Kenya	166807.8 (115815.9 to 257071.4)	766.9 (514.1 to 1250.5)	0.419 (0.274 to 0.564)	-0.723 (-0.921 to -0.525)
Kiribati	1102.8 (817.3 to 1676.6)	1684.3 (1265.1 to 2595.5)	-0.965 (-1.028 to -0.902)	-0.697 (-0.907 to -0.486)
Kuwait	5039.7 (4286.8 to 5886.4)	160.5 (138.5 to 185.7)	-0.765 (-0.961 to -0.569)	0.557 (-1.406 to 2.559)
Kyrgyzstan	28170.7 (24378.1 to 32270.7)	627.2 (542.7 to 714.8)	-4.480 (-5.021 to -3.936)	-1.106 (-1.223 to -0.989)
Lao People's Democratic Republic	49655.1 (37453.6 to 63797.7)	1176.8 (892.8 to 1502.1)	-1.713 (-1.798 to -1.629)	-0.672 (-0.752 to -0.592)
Latvia	8287.6 (7252.2 to 9468.7)	214.7 (185.9 to 243.6)	-1.235 (-1.539 to -0.930)	0.135 (-0.925 to 1.206)
Lebanon	30023.0 (26252.9 to 34370.4)	477.8 (416.9 to 545.9)	-0.944 (-1.076 to -0.811)	-0.405 (-0.677 to -0.132)
Lesotho	17137.4 (12499.1 to 22262.1)	1616.3 (1182.3 to 2071.6)	1.103 (0.774 to 1.433)	-2.353 (-3.399 to -1.295)
Liberia	14129.4 (11201.6 to 18062.4)	652.1 (513.7 to 830.6)	-0.485 (-0.647 to -0.322)	0.513 (0.480 to 0.546)
Libya	26120.5 (21181.6 to 32666.4)	504.6 (410.1 to 628.2)	0.527 (0.384 to 0.670)	-0.677 (-1.188 to -0.164)
Lithuania	14095.1 (12524.6 to 15466.2)	249.9 (222.8 to 275.5)	-3.257 (-3.423 to -3.091)	-0.276 (-1.198 to 0.655)
Luxembourg	4397.5 (3974.3 to 4849.9)	399.6 (360.4 to 442.1)	-1.082 (-1.170 to -0.993)	-3.399 (-5.543 to -1.206)
Madagascar	120101.6 (92583.3 to	1147.8 (892.9 to 1467.0)	-0.468 (-0.541 to -0.395)	0.103 (0.028 to 0.178)

	153445.6)			
Malawi	52175.4 (43677.2 to 62489.7)	704.6 (592.4 to 839.1)	-0.408 (-0.582 to -0.234)	-0.489 (-0.822 to -0.156)
Malaysia	167980.4 (151924.5 to 184807.5)	628.1 (565.4 to 692.1)	-1.668 (-1.900 to -1.435)	0.403 (-10.569 to 12.720)
Maldives	2125.9 (1828.1 to 2479.2)	696.3 (597.4 to 814.6)	-3.659 (-3.805 to -3.512)	-1.441 (-4.898 to 2.142)
Mali	94056.5 (75273.8 to 120846.3)	1042.2 (837.2 to 1325.0)	-0.174 (-0.287 to -0.060)	-0.845 (-0.880 to -0.810)
Malta	2474.9 (2214.2 to 2771.4)	244.6 (218.6 to 274.1)	-2.136 (-2.311 to -1.961)	-2.359 (-7.124 to 2.651)
Marshall Islands	465.7 (359.1 to 605.3)	1467.5 (1126.8 to 1862.4)	-1.135 (-1.208 to -1.063)	-1.296 (-1.415 to -1.177)
Mauritania	10337.0 (8481.7 to 12262.5)	482.9 (395.8 to 574.9)	-1.347 (-1.663 to -1.030)	0.945 (-0.090 to 1.991)
Mauritius	6730.2 (6244.8 to 7258.3)	388.6 (361.0 to 420.1)	-1.956 (-2.145 to -1.766)	0.299 (-3.219 to 3.945)
Mexico	615402.8 (555650.1 to 675742.3)	519.7 (469.8 to 570.4)	-1.178 (-1.283 to -1.074)	-0.344 (-1.179 to 0.498)
Micronesia (Federated States of)	935.2 (743.8 to 1205.6)	1396.6 (1121.9 to 1761.2)	-1.799 (-1.906 to -1.691)	-0.727 (-0.828 to -0.625)
Monaco	299.3 (249.3 to 351.2)	292.1 (246.1 to 339.5)	-0.379 (-0.458 to -0.300)	-0.747 (-1.215 to -0.278)
Mongolia	9333.9 (7792.6 to 10938.0)	453.7 (376.6 to 532.2)	-2.712 (-2.936 to -2.487)	-2.061 (-4.254 to 0.182)
Montenegro	1760.1 (1508.5 to 2053.3)	187.5 (161.3 to 217.7)	0.104 (0.029 to 0.178)	-1.181 (-4.150 to 1.879)
Morocco	181710.7 (149072.0 to 210580.3)	557.9 (461.2 to 645.7)	0.450 (0.370 to 0.529)	-0.217 (-0.649 to 0.217)
Mozambique	76327.7 (61074.4 to 91790.4)	683.4 (550.0 to 823.5)	0.400 (0.272 to 0.529)	-1.648 (-1.985 to -1.309)
Myanmar	860214.5 (707432.7 to 1037228.7)	1958.9 (1604.1 to 2354.0)	-1.322 (-1.433 to -1.212)	-0.229 (-0.560 to 0.103)

Namibia	15260.2 (12270.4 to 18867.6)	1171.8 (951.3 to 1457.2)	-0.771 (-0.968 to -0.573)	-1.331 (-1.709 to -0.953)
Nauru	86.8 (64.1 to 116.6)	1614.6 (1176.7 to 2426.5)	-1.183 (-1.351 to -1.016)	-1.088 (-1.124 to -1.052)
Nepal	610592.9 (484943.1 to 752714.4)	2836.0 (2275.3 to 3485.0)	-0.648 (-0.858 to -0.438)	-0.490 (-0.832 to -0.147)
Netherlands	197388.8 (179645.5 to 212040.9)	537.8 (493.2 to 576.9)	-0.724 (-0.850 to -0.598)	-2.305 (-2.894 to -1.712)
New Zealand	37935.6 (34308.5 to 40625.6)	431.8 (393.2 to 462.1)	-1.726 (-1.884 to -1.567)	-1.883 (-4.349 to 0.647)
Nicaragua	17250.7 (14907.4 to 19802.5)	381.7 (327.6 to 437.4)	0.442 (0.158 to 0.727)	-3.651 (-5.642 to -1.618)
Niger	57550.2 (43963.2 to 71564.1)	702.1 (524.5 to 871.3)	-0.413 (-0.625 to -0.202)	-0.232 (-0.274 to -0.191)
Nigeria	444788.1 (382136.5 to 518374.1)	490.5 (424.1 to 569.1)	-0.595 (-0.639 to -0.550)	-0.632 (-1.096 to -0.166)
Niue	20.4 (16.7 to 24.7)	985.7 (811.6 to 1191.9)	-1.633 (-1.725 to -1.542)	0.331 (-0.746 to 1.418)
North Macedonia	13878.3 (10924.7 to 18496.8)	454.4 (362.4 to 595.0)	-1.082 (-1.216 to -0.947)	-1.868 (-2.302 to -1.433)
Northern Mariana Islands	419.6 (369.7 to 479.8)	940.6 (840.4 to 1055.8)	-1.168 (-1.232 to -1.104)	0.666 (-5.667 to 7.425)
Norway	52691.3 (48028.5 to 56308.1)	497.3 (456.3 to 532.2)	1.459 (1.076 to 1.844)	-1.691 (-3.643 to 0.300)
Oman	7342.7 (6306.7 to 8827.2)	380.9 (326.2 to 458.2)	-0.481 (-0.653 to -0.308)	-3.101 (-5.217 to -0.938)
Pakistan	1657341.7 (1374667.5 to 1999431.0)	1541.7 (1287.5 to 1855.9)	-0.682 (-0.902 to -0.460)	-1.634 (-2.224 to -1.040)
Palau	247.7 (202.8 to 295.8)	1338.1 (1120.3 to 1589.8)	-0.563 (-0.623 to -0.504)	-0.148 (-0.424 to 0.129)
Palestine	9335.2 (8315.0 to 10596.7)	397.2 (353.2 to 454.7)	-1.429 (-1.621 to -1.237)	0.398 (-0.815 to 1.626)

Panama	14074.4 (11594.6 to 16339.8)	313.2 (258.1 to 363.2)	-0.864 (-1.130 to -0.597)	-0.895 (-2.195 to 0.422)
Papua New Guinea	131564.0 (102978.8 to 164213.6)	3004.4 (2404.3 to 3732.8)	-0.689 (-0.734 to -0.644)	-0.764 (-1.372 to -0.152)
Paraguay	21601.9 (17738.5 to 26397.4)	385.2 (315.6 to 470.0)	0.538 (0.431 to 0.645)	-3.655 (-6.146 to -1.098)
Peru	67067.9 (51968.3 to 85453.2)	200.0 (154.3 to 255.7)	-0.859 (-0.998 to -0.720)	-4.442 (-4.746 to -4.137)
Philippines	632847.5 (553583.6 to 728840.2)	807.0 (707.1 to 923.8)	-0.453 (-0.526 to -0.380)	-1.238 (-2.455 to -0.005)
Poland	237264.8 (216345.7 to 259512.3)	327.8 (298.6 to 358.5)	-1.752 (-1.874 to -1.629)	-0.165 (-0.712 to 0.384)
Portugal	95187.1 (85034.2 to 103420.9)	344.5 (312.3 to 373.1)	-1.827 (-2.041 to -1.612)	-1.488 (-2.109 to -0.862)
Puerto Rico	27123.0 (23309.6 to 31270.7)	354.6 (305.6 to 406.9)	-1.289 (-1.606 to -0.970)	-0.630 (-1.216 to -0.040)
Qatar	3225.9 (2703.1 to 3773.3)	336.2 (287.5 to 395.6)	-2.192 (-2.609 to -1.773)	2.462 (-1.128 to 6.184)
Republic of Korea	266056.1 (230704.1 to 309043.0)	289.1 (251.7 to 335.7)	-2.088 (-2.272 to -1.905)	-1.004 (-1.971 to -0.028)
Republic of Moldova	18449.7 (16631.3 to 20505.4)	314.8 (283.8 to 349.0)	-4.328 (-4.714 to -3.940)	-0.411 (-0.844 to 0.024)
Romania	165985.4 (150232.7 to 181410.8)	459.2 (415.1 to 503.4)	-2.705 (-2.989 to -2.419)	-0.232 (-1.087 to 0.631)
Russian Federation	828949.9 (770974.5 to 896492.6)	352.0 (328.3 to 379.9)	-2.742 (-3.053 to -2.429)	-0.715 (-0.850 to -0.580)
Rwanda	48502.9 (36946.1 to	800.0 (611.7 to 1028.7)	-2.743 (-3.079 to -2.406)	0.726 (0.577 to 0.875)

	61939.8)			
Saint Kitts and Nevis	220.2 (188.1 to 252.0)	345.5 (297.2 to 389.9)	0.505 (0.361 to 0.650)	-0.200 (-0.747 to 0.351)
Saint Lucia	1151.4 (956.2 to 1360.8)	487.5 (404.9 to 574.5)	-0.420 (-0.640 to -0.200)	0.068 (-0.117 to 0.254)
Saint Vincent and the Grenadines	331.8 (298.8 to 370.4)	240.1 (216.9 to 267.4)	1.240 (0.953 to 1.528)	0.124 (-0.297 to 0.548)
Samoa	1884.7 (1526.1 to 2351.1)	1396.4 (1123.6 to 1732.6)	-1.186 (-1.277 to -1.096)	-0.904 (-1.127 to -0.682)
San Marino	148.4 (119.0 to 181.1)	183.8 (148.1 to 222.9)	-0.997 (-1.195 to -0.798)	-12.303 (-22.246 to -1.087)
Sao Tome and Principe	1366.8 (1090.0 to 1689.5)	1269.7 (1005.6 to 1563.8)	-0.183 (-0.327 to -0.040)	-1.251 (-1.254 to -1.249)
Saudi Arabia	105295.2 (87334.0 to 125544.8)	533.1 (452.6 to 618.8)	-0.922 (-0.966 to -0.878)	-0.705 (-0.917 to -0.492)
Senegal	48849.4 (39869.6 to 58522.4)	632.1 (513.7 to 758.4)	-0.400 (-0.731 to -0.069)	-0.424 (-0.571 to -0.278)
Serbia	79311.0 (67838.9 to 91575.6)	475.2 (406.9 to 548.7)	-1.285 (-1.378 to -1.191)	-0.538 (-3.015 to 2.003)
Seychelles	586.3 (520.2 to 655.9)	538.6 (475.6 to 600.3)	-1.209 (-1.323 to -1.095)	-5.029 (-13.229 to 3.946)
Sierra Leone	25126.5 (19799.9 to 32414.9)	646.9 (505.8 to 831.9)	-0.337 (-0.494 to -0.179)	-0.159 (-0.435 to 0.119)
Singapore	12295.8 (11019.3 to 13602.4)	146.5 (131.4 to 161.7)	-5.471 (-5.586 to -5.356)	-2.194 (-3.547 to -0.823)
Slovakia	25679.3 (22461.2 to 29827.1)	272.0 (238.6 to 314.6)	-0.516 (-0.631 to -0.402)	-0.222 (-0.687 to 0.246)
Slovenia	12947.4 (11556.9 to 14366.3)	277.6 (245.4 to 308.4)	-2.848 (-3.083 to -2.613)	-1.910 (-2.724 to -1.090)
Solomon Islands	4272.6 (3482.7 to 5280.7)	1368.2 (1142.8 to 1653.2)	-0.893 (-0.972 to -0.815)	-0.791 (-1.653 to 0.078)
Somalia	64554.2 (44391.0 to 85201.8)	1040.2 (683.9 to 1374.6)	-0.782 (-0.838 to -0.725)	-1.248 (-1.768 to -0.726)

South Africa	382073.4 (354297.2 to 418560.8)	853.4 (789.3 to 936.0)	-0.325 (-0.653 to 0.004)	0.003 (-0.463 to 0.472)
South Sudan	31764.7 (22898.6 to 42332.0)	863.7 (616.8 to 1149.4)	-0.798 (-1.026 to -0.569)	0.194 (-0.611 to 1.005)
Spain	464294.8 (411417.5 to 504946.9)	410.2 (370.3 to 444.1)	-1.678 (-1.814 to -1.541)	-2.368 (-2.588 to -2.148)
Sri Lanka	219257.5 (158404.2 to 280074.7)	864.2 (631.5 to 1103.1)	-1.396 (-1.606 to -1.186)	-2.359 (-10.726 to 6.793)
Sudan	124034.1 (87671.1 to 168666.3)	623.2 (442.2 to 841.6)	-1.011 (-1.053 to -0.969)	-0.033 (-0.201 to 0.135)
Suriname	2212.6 (1777.9 to 2703.1)	355.3 (284.8 to 434.6)	-0.396 (-0.579 to -0.212)	-5.130 (-7.936 to -2.238)
Sweden	78587.4 (69696.6 to 86779.5)	339.4 (304.6 to 374.6)	0.273 (0.016 to 0.530)	-2.996 (-3.026 to -2.966)
Switzerland	54985.0 (48849.9 to 60797.1)	289.3 (259.2 to 319.9)	-1.301 (-1.364 to -1.237)	-1.809 (-3.218 to -0.378)
Syrian Arab Republic	73016.0 (58644.5 to 89317.5)	605.9 (498.5 to 740.4)	-0.209 (-0.314 to -0.105)	-0.176 (-0.505 to 0.154)
Taiwan (Province of China)	148677.5 (134659.0 to 163583.0)	343.4 (311.5 to 377.5)	-1.941 (-2.110 to -1.771)	-1.651 (-3.690 to 0.432)
Tajikistan	34175.5 (26488.7 to 44558.6)	637.1 (492.3 to 826.7)	-2.045 (-2.266 to -1.823)	0.249 (-0.431 to 0.935)
Thailand	487665.2 (401670.7 to 595684.9)	461.1 (381.2 to 559.9)	-3.695 (-3.952 to -3.438)	0.482 (0.096 to 0.869)
Timor-Leste	8459.5 (6572.2 to 10395.1)	1047.9 (814.4 to 1288.8)	-0.584 (-0.696 to -0.471)	-0.478 (-0.781 to -0.174)
Togo	27088.3 (21352.5 to 34667.7)	705.1 (558.7 to 907.9)	-0.280 (-0.419 to -0.140)	-0.037 (-0.264 to 0.191)

Tokelau	13.6 (10.4 to 18.3)	921.2 (704.9 to 1227.6)	-1.525 (-1.590 to -1.459)	0.402 (-0.870 to 1.690)
Tonga	776.1 (640.0 to 914.8)	988.9 (814.2 to 1161.4)	-0.914 (-1.021 to -0.806)	-0.812 (-1.047 to -0.576)
Trinidad and Tobago	4914.1 (4008.5 to 5932.0)	256.2 (210.3 to 308.6)	-0.618 (-0.765 to -0.471)	0.092 (-0.683 to 0.873)
Tunisia	58175.8 (46751.6 to 74001.0)	460.9 (370.7 to 585.5)	-0.096 (-0.150 to -0.041)	-0.398 (-0.614 to -0.182)
Turkmenistan	12562.4 (10325.2 to 15261.6)	307.8 (254.5 to 369.5)	-3.969 (-4.538 to -3.396)	-0.074 (-0.110 to -0.037)
Tuvalu	110.3 (85.6 to 139.3)	1138.3 (890.7 to 1437.5)	-2.008 (-2.100 to -1.916)	-1.142 (-1.168 to -1.115)
T 眉 rkiye	761035.4 (651582.7 to 881753.2)	850.2 (728.0 to 983.5)	-0.898 (-1.151 to -0.643)	-1.037 (-2.446 to 0.393)
Uganda	101881.7 (75092.7 to 128956.8)	693.4 (506.3 to 880.8)	-1.589 (-1.750 to -1.429)	-0.263 (-0.605 to 0.081)
Ukraine	207495.3 (169403.8 to 253607.9)	274.4 (225.1 to 334.2)	-5.678 (-6.130 to -5.225)	-2.010 (-4.760 to 0.819)
United Arab Emirates	24096.1 (20567.6 to 27878.8)	594.6 (488.7 to 713.9)	-0.238 (-0.679 to 0.206)	-13.478 (-22.991 to -2.789)
United Kingdom	774612.0 (716224.9 to 816863.8)	571.5 (532.6 to 602.0)	-0.650 (-0.732 to -0.568)	-2.518 (-4.499 to -0.496)
United Republic of Tanzania	138364.4 (113030.6 to 167939.8)	543.5 (445.2 to 659.6)	-0.946 (-1.012 to -0.880)	0.211 (0.099 to 0.324)
United States Virgin Islands	324.4 (264.2 to 398.9)	182.9 (150.0 to 224.1)	-1.143 (-1.297 to -0.988)	-6.017 (-10.833 to -0.941)
United States of America	4646118.1 (4302650.4 to 4904223.8)	777.9 (725.2 to 819.9)	0.221 (0.059 to 0.383)	-0.153 (-0.818 to 0.516)
Uruguay	36505.2 (33925.3 to 38640.4)	642.3 (600.5 to 679.0)	-0.071 (-0.195 to 0.053)	-0.565 (-6.478 to 5.722)

Uzbekistan	57699.9 (49662.9 to 65971.2)	238.4 (206.5 to 271.8)	-3.860 (-4.502 to -3.214)	-0.338 (-0.373 to -0.302)
Vanuatu	2767.2 (1800.0 to 3606.6)	1709.1 (1132.6 to 2218.1)	-1.196 (-1.272 to -1.120)	-0.622 (-0.721 to -0.524)
Venezuela (Bolivarian Republic of)	133543.3 (106608.7 to 166502.8)	462.8 (370.7 to 574.2)	0.232 (-0.056 to 0.521)	1.641 (1.407 to 1.875)
Viet Nam	776752.4 (626580.1 to 929720.0)	863.3 (702.8 to 1024.5)	-0.598 (-0.672 to -0.523)	-0.993 (-1.044 to -0.943)
Yemen	101184.2 (79276.7 to 127402.3)	730.2 (567.1 to 920.1)	-0.734 (-0.809 to -0.659)	0.800 (0.605 to 0.996)
Zambia	48749.1 (39118.6 to 59593.6)	705.5 (572.0 to 851.1)	-0.595 (-0.730 to -0.459)	-1.482 (-2.136 to -0.824)
Zimbabwe	47044.4 (38407.6 to 56970.4)	721.1 (594.0 to 856.2)	0.632 (0.376 to 0.889)	-1.281 (-1.619 to -0.941)

EAPC: Estimated Annual Percentage Change; ASRs: Age-standardized rates; COPD: Chronic Obstructive Pulmonary Disease; EAPCs for ASRs from 1990 to 2019 (%, 95% UI) were calculated using GBD 2021.

Table S4. The percent in all-cause prevalence and EAPC for percent of COPD at national level, 1990-2021 and 2019-2021.

location	Percent, in 2021 (95% UI)	EAPC for percent from 1990 to 2021 (95% CI)	EAPC for percent from 2019 to 2021 (95% CI)
Afghanistan	0.980 (0.885 to 1.084)	-1.733 (-1.970 to -1.495)	-0.026 (-0.702 to 0.655)
Albania	3.910 (3.402 to 4.475)	3.371 (3.256 to 3.487)	2.064 (1.735 to 2.394)
Algeria	2.219 (1.960 to 2.496)	2.185 (2.125 to 2.245)	2.009 (1.960 to 2.058)
American Samoa	1.872 (1.658 to 2.116)	1.433 (1.335 to 1.531)	2.915 (2.452 to 3.380)
Andorra	4.881 (4.364 to 5.492)	1.303 (1.155 to 1.452)	1.656 (1.642 to 1.669)
Angola	0.680 (0.595 to 0.762)	-0.494 (-0.592 to -0.396)	1.556 (0.939 to 2.176)
Antigua and Barbuda	1.556 (1.336 to 1.786)	1.671 (1.458 to 1.885)	2.791 (2.752 to 2.831)
Argentina	2.046 (1.829 to 2.300)	0.833 (0.735 to 0.931)	0.571 (0.211 to 0.933)
Armenia	3.475 (3.089 to 3.925)	2.105 (2.038 to 2.172)	0.460 (-0.844 to 1.780)
Australia	2.877 (2.550 to 3.333)	0.367 (0.262 to 0.473)	-1.749 (-3.367 to -0.105)
Austria	5.956 (5.332 to 6.500)	1.018 (0.908 to 1.129)	-0.228 (-1.093 to 0.644)
Azerbaijan	2.144 (1.889 to 2.429)	0.912 (0.779 to 1.045)	1.614 (1.538 to 1.690)
Bahamas	1.388 (1.222 to 1.563)	2.096 (2.048 to 2.143)	1.183 (0.746 to 1.621)
Bahrain	1.607 (1.415 to 1.843)	1.108 (0.987 to 1.229)	3.987 (3.290 to 4.689)
Bangladesh	2.519 (2.276 to 2.806)	2.346 (2.202 to 2.489)	2.704 (2.604 to 2.804)
Barbados	2.203 (1.899 to 2.505)	1.794 (1.729 to 1.859)	2.108 (1.308 to 2.914)
Belarus	3.498 (3.087 to 3.953)	0.340 (0.270 to 0.409)	0.699 (0.220 to 1.179)
Belgium	5.153 (4.632 to 5.771)	0.902 (0.851 to 0.954)	-0.262 (-0.408 to -0.115)
Belize	1.306 (1.148 to 1.464)	1.577 (1.448 to 1.707)	1.448 (1.305 to 1.591)
Benin	0.797 (0.704 to 0.884)	0.110 (0.016 to 0.203)	1.054 (0.933 to 1.175)
Bermuda	3.224 (2.761 to 3.695)	2.765 (2.724 to 2.806)	2.407 (1.580 to 3.240)
Bhutan	2.320 (2.093 to 2.595)	1.980 (1.892 to 2.069)	2.323 (1.858 to 2.791)
Bolivia (Plurinational State of)	1.524 (1.335 to 1.736)	1.525 (1.460 to 1.589)	0.163 (-0.029 to 0.356)

Bosnia and Herzegovina	4.817 (4.274 to 5.434)	2.435 (2.317 to 2.554)	1.153 (0.398 to 1.914)
Botswana	1.365 (1.207 to 1.532)	1.213 (1.091 to 1.336)	2.210 (1.730 to 2.691)
Brazil	2.943 (2.607 to 3.296)	1.904 (1.883 to 1.925)	2.401 (1.223 to 3.593)
Brunei Darussalam	1.233 (1.086 to 1.385)	0.528 (0.379 to 0.677)	3.123 (3.060 to 3.186)
Bulgaria	5.052 (4.378 to 5.745)	1.303 (1.259 to 1.347)	0.848 (-0.723 to 2.444)
Burkina Faso	0.773 (0.686 to 0.865)	0.125 (0.033 to 0.218)	0.565 (0.379 to 0.752)
Burundi	0.851 (0.757 to 0.941)	-0.075 (-0.215 to 0.065)	0.962 (0.847 to 1.077)
Cabo Verde	0.894 (0.780 to 1.004)	1.057 (0.830 to 1.285)	2.157 (2.147 to 2.167)
Cambodia	1.645 (1.468 to 1.863)	1.733 (1.623 to 1.844)	2.144 (1.961 to 2.328)
Cameroon	0.777 (0.692 to 0.873)	0.043 (-0.055 to 0.142)	1.410 (1.202 to 1.618)
Canada	4.304 (3.842 to 4.811)	1.358 (1.081 to 1.635)	1.007 (-0.352 to 2.384)
Central African Republic	0.956 (0.841 to 1.065)	0.222 (0.072 to 0.371)	0.965 (0.543 to 1.389)
Chad	0.738 (0.653 to 0.825)	-0.557 (-0.650 to -0.465)	0.011 (-0.116 to 0.138)
Chile	1.723 (1.496 to 1.969)	1.376 (1.363 to 1.389)	1.035 (0.039 to 2.042)
China	3.711 (3.293 to 4.189)	1.983 (1.964 to 2.003)	2.443 (2.239 to 2.648)
Colombia	2.771 (2.494 to 3.076)	2.435 (2.406 to 2.464)	2.100 (1.828 to 2.373)
Comoros	1.079 (0.942 to 1.216)	1.179 (1.085 to 1.273)	2.223 (1.591 to 2.858)
Congo	0.928 (0.815 to 1.044)	0.393 (0.238 to 0.549)	2.423 (2.323 to 2.523)
Cook Islands	2.612 (2.213 to 2.994)	1.898 (1.806 to 1.990)	2.700 (1.884 to 3.522)
Costa Rica	2.532 (2.196 to 2.844)	2.509 (2.447 to 2.572)	3.161 (2.780 to 3.544)
Croatia	5.470 (4.883 to 6.035)	2.346 (2.287 to 2.405)	1.403 (1.200 to 1.606)
Cuba	3.715 (3.313 to 4.133)	2.764 (2.693 to 2.836)	0.599 (0.068 to 1.132)
Cyprus	3.850 (3.415 to 4.304)	0.970 (0.934 to 1.005)	1.530 (1.505 to 1.555)
Czechia	5.036 (4.509 to 5.519)	2.007 (1.967 to 2.048)	1.045 (1.043 to 1.046)
C 么 te d'Ivoire	0.848 (0.752 to 0.952)	0.797 (0.733 to 0.862)	1.409 (0.968 to 1.852)
Democratic People's Republic of	3.479 (3.137 to 3.879)	1.075 (1.058 to 1.091)	1.467 (1.345 to 1.588)

Korea			
Democratic Republic of the Congo	0.907 (0.806 to 1.026)	0.369 (0.322 to 0.417)	1.456 (1.294 to 1.617)
Denmark	5.610 (4.974 to 6.216)	0.410 (0.320 to 0.501)	1.118 (0.667 to 1.570)
Djibouti	0.862 (0.746 to 0.973)	1.238 (1.075 to 1.400)	2.476 (2.195 to 2.757)
Dominica	1.805 (1.541 to 2.072)	1.620 (1.559 to 1.680)	2.072 (1.518 to 2.629)
Dominican Republic	1.596 (1.387 to 1.821)	2.442 (2.396 to 2.488)	1.208 (0.843 to 1.574)
Ecuador	1.615 (1.412 to 1.838)	1.964 (1.932 to 1.997)	0.738 (0.382 to 1.095)
Egypt	1.555 (1.363 to 1.774)	1.279 (1.237 to 1.321)	1.570 (1.011 to 2.132)
El Salvador	1.951 (1.686 to 2.228)	2.265 (2.244 to 2.286)	1.208 (0.318 to 2.105)
Equatorial Guinea	0.702 (0.616 to 0.788)	-0.778 (-1.071 to -0.483)	1.544 (1.407 to 1.680)
Eritrea	0.806 (0.710 to 0.916)	0.598 (0.487 to 0.708)	1.964 (1.325 to 2.608)
Estonia	3.171 (2.788 to 3.605)	2.264 (2.165 to 2.364)	-2.181 (-4.367 to 0.054)
Eswatini	1.058 (0.934 to 1.180)	0.931 (0.838 to 1.025)	1.161 (0.813 to 1.510)
Ethiopia	0.837 (0.744 to 0.926)	0.036 (-0.128 to 0.200)	1.333 (1.330 to 1.336)
Fiji	1.341 (1.183 to 1.537)	0.768 (0.718 to 0.819)	1.066 (0.423 to 1.714)
Finland	4.864 (4.251 to 5.530)	1.362 (1.313 to 1.410)	1.856 (0.989 to 2.730)
France	4.713 (4.202 to 5.299)	1.191 (1.140 to 1.242)	0.542 (0.369 to 0.715)
Gabon	0.930 (0.810 to 1.053)	0.289 (0.085 to 0.494)	2.063 (1.691 to 2.436)
Gambia	0.886 (0.791 to 0.986)	0.704 (0.682 to 0.726)	0.689 (0.631 to 0.747)
Georgia	3.486 (3.049 to 3.944)	1.899 (1.833 to 1.964)	0.316 (0.245 to 0.388)
Germany	6.396 (5.768 to 7.094)	1.223 (1.103 to 1.343)	0.389 (-0.381 to 1.165)
Ghana	0.958 (0.852 to 1.059)	0.959 (0.891 to 1.027)	1.308 (1.227 to 1.389)
Greece	6.230 (5.590 to 6.986)	1.757 (1.673 to 1.840)	0.858 (0.077 to 1.646)
Greenland	3.187 (2.874 to 3.548)	2.123 (2.077 to 2.168)	1.805 (1.393 to 2.219)
Grenada	1.634 (1.427 to 1.839)	1.420 (1.209 to 1.631)	1.654 (1.491 to 1.817)
Guam	2.211 (1.871 to 2.507)	2.332 (2.263 to 2.401)	2.728 (2.720 to 2.737)

Guatemala	1.388 (1.233 to 1.570)	1.972 (1.881 to 2.064)	2.181 (2.048 to 2.314)
Guinea	0.905 (0.804 to 1.004)	-0.175 (-0.211 to -0.140)	-0.028 (-0.149 to 0.093)
Guinea-Bissau	0.796 (0.709 to 0.889)	0.112 (0.002 to 0.222)	0.584 (0.329 to 0.839)
Guyana	1.200 (1.035 to 1.364)	2.261 (2.199 to 2.324)	1.612 (0.978 to 2.250)
Haiti	1.124 (0.989 to 1.267)	0.497 (0.461 to 0.534)	0.524 (0.487 to 0.562)
Honduras	1.623 (1.436 to 1.840)	1.646 (1.628 to 1.664)	0.890 (0.808 to 0.971)
Hungary	5.902 (5.291 to 6.451)	1.756 (1.719 to 1.794)	0.459 (0.348 to 0.570)
Iceland	4.747 (4.253 to 5.244)	0.680 (0.579 to 0.781)	1.588 (1.487 to 1.690)
India	2.583 (2.337 to 2.822)	1.585 (1.549 to 1.621)	1.334 (1.124 to 1.545)
Indonesia	1.883 (1.647 to 2.117)	1.572 (1.530 to 1.613)	2.235 (1.993 to 2.477)
Iran (Islamic Republic of)	1.924 (1.693 to 2.161)	2.896 (2.871 to 2.921)	1.807 (1.304 to 2.312)
Iraq	1.259 (1.110 to 1.423)	1.325 (1.175 to 1.475)	1.934 (0.881 to 2.998)
Ireland	4.092 (3.686 to 4.553)	0.529 (0.409 to 0.650)	2.625 (2.433 to 2.818)
Israel	3.161 (2.785 to 3.527)	0.569 (0.559 to 0.580)	0.677 (0.312 to 1.043)
Italy	5.485 (4.741 to 6.191)	1.361 (1.300 to 1.423)	0.544 (-0.377 to 1.474)
Jamaica	1.926 (1.728 to 2.146)	1.599 (1.563 to 1.634)	1.587 (1.067 to 2.109)
Japan	4.204 (3.539 to 4.946)	2.130 (2.020 to 2.241)	1.386 (0.370 to 2.413)
Jordan	1.421 (1.247 to 1.605)	1.414 (1.313 to 1.516)	3.136 (2.731 to 3.542)
Kazakhstan	2.623 (2.378 to 2.943)	0.965 (0.876 to 1.054)	0.083 (-0.101 to 0.268)
Kenya	0.826 (0.722 to 0.926)	0.856 (0.745 to 0.968)	2.044 (1.205 to 2.889)
Kiribati	1.408 (1.262 to 1.585)	0.465 (0.399 to 0.532)	1.069 (0.659 to 1.481)
Kuwait	1.495 (1.320 to 1.687)	1.590 (1.487 to 1.694)	2.726 (2.297 to 3.157)
Kyrgyzstan	1.926 (1.723 to 2.163)	-0.410 (-0.490 to -0.330)	0.785 (0.302 to 1.270)
Lao People's Democratic Republic	1.600 (1.443 to 1.788)	0.687 (0.509 to 0.866)	2.234 (1.516 to 2.958)
Latvia	3.787 (3.314 to 4.281)	1.482 (1.330 to 1.634)	1.574 (1.307 to 1.842)
Lebanon	2.885 (2.505 to 3.286)	2.393 (2.227 to 2.559)	0.596 (-1.456 to 2.691)

Lesotho	1.462 (1.299 to 1.644)	0.429 (0.373 to 0.486)	0.159 (-0.136 to 0.455)
Liberia	0.812 (0.718 to 0.912)	-0.360 (-0.534 to -0.186)	1.498 (1.224 to 1.773)
Libya	1.819 (1.618 to 2.048)	2.090 (1.972 to 2.207)	2.122 (1.302 to 2.949)
Lithuania	3.910 (3.463 to 4.430)	1.285 (1.174 to 1.396)	2.029 (1.775 to 2.283)
Luxembourg	4.383 (3.920 to 4.881)	0.305 (0.267 to 0.343)	0.412 (0.286 to 0.539)
Madagascar	0.893 (0.797 to 1.000)	0.273 (0.138 to 0.408)	1.622 (1.037 to 2.211)
Malawi	0.723 (0.636 to 0.813)	0.414 (0.310 to 0.518)	1.575 (1.239 to 1.912)
Malaysia	1.645 (1.426 to 1.855)	1.716 (1.581 to 1.852)	2.812 (2.319 to 3.307)
Maldives	1.649 (1.469 to 1.843)	1.457 (1.381 to 1.532)	2.553 (2.225 to 2.883)
Mali	0.819 (0.726 to 0.905)	-0.211 (-0.281 to -0.141)	-0.014 (-0.291 to 0.264)
Malta	4.652 (4.029 to 5.255)	1.575 (1.536 to 1.613)	1.990 (1.948 to 2.032)
Marshall Islands	1.336 (1.207 to 1.484)	1.292 (1.145 to 1.438)	2.451 (2.263 to 2.639)
Mauritania	0.846 (0.746 to 0.963)	0.307 (0.189 to 0.425)	1.431 (1.050 to 1.813)
Mauritius	2.352 (2.079 to 2.663)	2.247 (2.126 to 2.368)	3.047 (3.016 to 3.078)
Mexico	2.229 (1.965 to 2.519)	2.459 (2.412 to 2.506)	0.689 (-0.351 to 1.741)
Micronesia (Federated States of)	1.609 (1.454 to 1.811)	0.934 (0.797 to 1.072)	1.836 (1.749 to 1.922)
Monaco	6.108 (5.316 to 6.917)	0.114 (0.054 to 0.174)	0.201 (-0.014 to 0.416)
Mongolia	1.543 (1.368 to 1.732)	1.016 (0.888 to 1.144)	1.523 (0.865 to 2.184)
Montenegro	3.175 (2.719 to 3.602)	1.975 (1.880 to 2.069)	0.150 (-0.134 to 0.436)
Morocco	2.238 (1.969 to 2.548)	2.397 (2.318 to 2.475)	2.219 (1.596 to 2.846)
Mozambique	0.690 (0.606 to 0.772)	-0.296 (-0.378 to -0.213)	1.036 (0.822 to 1.251)
Myanmar	2.359 (2.140 to 2.639)	1.178 (1.145 to 1.212)	1.397 (1.387 to 1.406)
Namibia	1.271 (1.119 to 1.429)	0.788 (0.636 to 0.942)	1.357 (0.641 to 2.078)
Nauru	1.229 (1.102 to 1.374)	0.187 (0.142 to 0.231)	0.999 (0.673 to 1.325)
Nepal	2.363 (2.192 to 2.552)	1.680 (1.591 to 1.769)	0.923 (0.501 to 1.347)
Netherlands	4.643 (4.159 to 5.167)	1.402 (1.291 to 1.514)	-2.019 (-2.735 to -1.297)

New Zealand	2.894 (2.437 to 3.398)	0.657 (0.570 to 0.743)	-1.334 (-3.064 to 0.427)
Nicaragua	1.663 (1.465 to 1.851)	2.505 (2.475 to 2.534)	1.642 (0.237 to 3.066)
Niger	0.758 (0.673 to 0.849)	0.300 (0.253 to 0.347)	0.306 (0.219 to 0.392)
Nigeria	0.838 (0.738 to 0.936)	-0.138 (-0.219 to -0.057)	0.883 (0.741 to 1.024)
Niue	2.436 (2.121 to 2.797)	0.335 (0.203 to 0.467)	0.615 (0.518 to 0.713)
North Macedonia	3.846 (3.357 to 4.454)	1.761 (1.698 to 1.823)	1.217 (0.630 to 1.807)
Northern Mariana Islands	1.886 (1.636 to 2.146)	2.383 (2.138 to 2.629)	3.287 (3.021 to 3.552)
Norway	5.285 (4.673 to 5.969)	0.762 (0.672 to 0.852)	0.756 (0.709 to 0.804)
Oman	1.186 (1.044 to 1.347)	0.904 (0.860 to 0.949)	1.797 (1.288 to 2.309)
Pakistan	1.416 (1.254 to 1.581)	0.071 (0.035 to 0.106)	0.122 (-0.303 to 0.548)
Palau	2.334 (2.049 to 2.629)	1.285 (1.166 to 1.404)	2.830 (2.115 to 3.549)
Palestine	1.191 (1.063 to 1.332)	0.748 (0.564 to 0.931)	2.928 (1.628 to 4.245)
Panama	2.036 (1.832 to 2.266)	1.975 (1.927 to 2.023)	2.460 (1.894 to 3.030)
Papua New Guinea	1.429 (1.321 to 1.552)	0.250 (0.186 to 0.315)	0.757 (0.530 to 0.985)
Paraguay	1.812 (1.592 to 2.050)	1.489 (1.433 to 1.546)	1.843 (1.271 to 2.419)
Peru	1.478 (1.276 to 1.687)	2.220 (2.075 to 2.365)	-3.297 (-6.836 to 0.376)
Philippines	1.686 (1.494 to 1.880)	0.992 (0.941 to 1.043)	1.431 (0.606 to 2.262)
Poland	4.216 (3.674 to 4.746)	1.596 (1.561 to 1.631)	1.648 (1.530 to 1.766)
Portugal	5.463 (4.908 to 6.122)	1.207 (1.184 to 1.230)	1.466 (1.164 to 1.769)
Puerto Rico	3.994 (3.471 to 4.477)	2.943 (2.908 to 2.977)	2.974 (1.753 to 4.210)
Qatar	1.120 (0.988 to 1.258)	0.344 (0.174 to 0.515)	1.760 (0.768 to 2.762)
Republic of Korea	4.215 (3.836 to 4.650)	3.632 (3.379 to 3.885)	1.141 (0.494 to 1.792)
Republic of Moldova	3.390 (2.957 to 3.880)	0.898 (0.833 to 0.963)	1.595 (1.472 to 1.717)
Romania	4.617 (4.113 to 5.171)	1.547 (1.485 to 1.609)	0.611 (-0.586 to 1.822)
Russian Federation	3.584 (3.135 to 4.056)	0.740 (0.699 to 0.782)	1.899 (1.550 to 2.249)
Rwanda	1.026 (0.904 to 1.145)	0.796 (0.559 to 1.034)	2.137 (1.959 to 2.316)

Saint Kitts and Nevis	1.682 (1.442 to 1.945)	1.420 (1.279 to 1.562)	1.820 (0.895 to 2.754)
Saint Lucia	2.387 (2.082 to 2.685)	2.891 (2.827 to 2.955)	2.406 (1.535 to 3.284)
Saint Vincent and the Grenadines	1.633 (1.428 to 1.858)	2.682 (2.605 to 2.760)	1.864 (0.856 to 2.881)
Samoa	1.608 (1.434 to 1.805)	0.512 (0.445 to 0.579)	0.783 (0.305 to 1.263)
San Marino	5.098 (4.414 to 5.818)	1.027 (0.975 to 1.080)	0.977 (0.103 to 1.858)
Sao Tome and Principe	1.260 (1.139 to 1.392)	0.647 (0.541 to 0.754)	2.257 (2.024 to 2.490)
Saudi Arabia	1.231 (1.073 to 1.395)	1.903 (1.831 to 1.974)	3.618 (2.581 to 4.665)
Senegal	0.947 (0.839 to 1.066)	0.789 (0.730 to 0.848)	0.955 (0.815 to 1.095)
Serbia	4.976 (4.447 to 5.569)	2.162 (2.067 to 2.258)	0.947 (0.437 to 1.460)
Seychelles	2.044 (1.786 to 2.321)	1.241 (1.171 to 1.312)	2.390 (1.994 to 2.789)
Sierra Leone	0.909 (0.812 to 1.010)	-0.186 (-0.335 to -0.037)	1.362 (1.046 to 1.680)
Singapore	1.434 (1.245 to 1.651)	0.607 (0.345 to 0.870)	3.594 (3.330 to 3.859)
Slovakia	3.678 (3.239 to 4.162)	1.782 (1.720 to 1.844)	1.069 (-0.130 to 2.282)
Slovenia	4.745 (4.155 to 5.343)	1.868 (1.832 to 1.904)	0.951 (-0.085 to 1.998)
Solomon Islands	1.346 (1.235 to 1.487)	0.530 (0.512 to 0.548)	0.994 (0.733 to 1.255)
Somalia	0.757 (0.678 to 0.840)	0.067 (0.005 to 0.130)	0.041 (-0.273 to 0.356)
South Africa	1.813 (1.603 to 2.022)	1.049 (1.027 to 1.071)	0.991 (0.860 to 1.123)
South Sudan	0.753 (0.666 to 0.847)	-0.127 (-0.391 to 0.136)	0.270 (0.170 to 0.371)
Spain	5.907 (5.313 to 6.573)	1.093 (0.999 to 1.188)	0.830 (-0.052 to 1.719)
Sri Lanka	2.302 (1.986 to 2.643)	2.048 (2.006 to 2.090)	3.205 (2.911 to 3.501)
Sudan	1.248 (1.113 to 1.396)	0.044 (-0.047 to 0.135)	1.356 (0.714 to 2.002)
Suriname	1.744 (1.531 to 1.970)	1.826 (1.735 to 1.918)	2.256 (2.045 to 2.468)
Sweden	6.654 (5.875 to 7.426)	0.498 (0.400 to 0.596)	0.562 (0.495 to 0.630)
Switzerland	5.312 (4.789 to 5.895)	0.571 (0.480 to 0.662)	0.050 (-0.142 to 0.242)
Syrian Arab Republic	2.270 (2.010 to 2.575)	2.787 (2.352 to 3.224)	2.857 (2.048 to 3.673)
Taiwan (Province of China)	2.745 (2.402 to 3.146)	1.917 (1.864 to 1.970)	2.159 (1.231 to 3.096)

Tajikistan	1.546 (1.384 to 1.726)	0.318 (0.148 to 0.487)	1.188 (1.181 to 1.194)
Thailand	2.742 (2.380 to 3.123)	2.108 (2.041 to 2.175)	1.884 (-0.562 to 4.390)
Timor-Leste	1.455 (1.302 to 1.646)	1.399 (1.351 to 1.447)	1.395 (0.197 to 2.607)
Togo	0.943 (0.827 to 1.060)	1.048 (0.964 to 1.131)	2.075 (2.064 to 2.087)
Tokelau	2.090 (1.817 to 2.376)	0.503 (0.313 to 0.692)	0.468 (0.142 to 0.794)
Tonga	1.552 (1.354 to 1.760)	0.302 (0.256 to 0.347)	1.090 (0.653 to 1.527)
Trinidad and Tobago	1.976 (1.703 to 2.268)	2.387 (2.329 to 2.446)	2.564 (2.223 to 2.907)
Tunisia	3.189 (2.838 to 3.569)	2.610 (2.552 to 2.668)	1.941 (1.048 to 2.841)
Turkmenistan	1.366 (1.207 to 1.546)	0.350 (0.198 to 0.502)	1.446 (0.894 to 2.001)
Tuvalu	1.779 (1.579 to 2.021)	0.026 (-0.044 to 0.096)	0.948 (0.442 to 1.457)
T 眉 rkiye	3.589 (3.261 to 3.953)	2.300 (2.207 to 2.393)	1.899 (1.468 to 2.331)
Uganda	0.706 (0.628 to 0.790)	-0.152 (-0.354 to 0.050)	1.650 (1.168 to 2.135)
Ukraine	3.496 (3.004 to 4.003)	-0.171 (-0.228 to -0.115)	1.292 (1.152 to 1.433)
United Arab Emirates	1.915 (1.713 to 2.161)	1.506 (1.134 to 1.880)	6.210 (5.568 to 6.856)
United Kingdom	6.448 (5.803 to 7.055)	0.833 (0.806 to 0.860)	-0.367 (-1.248 to 0.521)
United Republic of Tanzania	0.803 (0.707 to 0.903)	0.491 (0.371 to 0.611)	1.688 (1.410 to 1.967)
United States Virgin Islands	3.051 (2.623 to 3.499)	3.751 (3.684 to 3.818)	3.383 (3.185 to 3.581)
United States of America	6.311 (5.968 to 6.606)	1.339 (1.279 to 1.399)	0.990 (0.750 to 1.230)
Uruguay	2.582 (2.241 to 2.932)	0.975 (0.920 to 1.030)	-0.420 (-2.083 to 1.272)
Uzbekistan	1.460 (1.301 to 1.644)	0.329 (0.231 to 0.427)	1.191 (0.958 to 1.424)
Vanuatu	1.364 (1.230 to 1.507)	0.557 (0.518 to 0.596)	1.406 (1.261 to 1.551)
Venezuela (Bolivarian Republic of)	2.399 (2.154 to 2.712)	2.957 (2.866 to 3.047)	4.576 (3.942 to 5.213)
Viet Nam	2.279 (2.028 to 2.572)	2.028 (1.962 to 2.095)	2.885 (2.463 to 3.309)
Yemen	1.166 (1.042 to 1.300)	0.570 (0.475 to 0.666)	1.410 (0.798 to 2.025)
Zambia	0.694 (0.610 to 0.773)	0.244 (0.088 to 0.400)	1.899 (1.366 to 2.436)
Zimbabwe	0.903 (0.789 to 1.024)	0.476 (0.374 to 0.578)	1.138 (0.868 to 1.408)

EAPC: Estimated Annual Percentage Change; COPD: Chronic Obstructive Pulmonary Disease; EAPCs for ASRs from 1990 to 2019 (%, 95% UI) were calculated using GBD 2021.

Table S5. The percent in all-cause deaths and EAPC for percent of COPD at national level, 1990-2021 and 2019-2021.

location	Percent, in 2021 (95% UI)	EAPC for percent from 1990 to 2021 (95% CI)	EAPC for percent from 2019 to 2021 (95% CI)
Afghanistan	1.398 (1.051 to 1.784)	-0.422 (-0.712 to -0.130)	-10.923 (-14.103 to -7.625)
Albania	2.165 (1.663 to 2.884)	-1.328 (-1.513 to -1.143)	-11.226 (-18.144 to -3.723)
Algeria	1.946 (1.607 to 2.332)	1.949 (1.634 to 2.265)	-14.944 (-20.962 to -8.467)
American Samoa	4.449 (4.003 to 4.903)	-0.787 (-0.908 to -0.665)	0.709 (-0.430 to 1.860)
Andorra	4.762 (3.802 to 5.642)	-0.443 (-0.601 to -0.285)	-6.994 (-15.117 to 1.907)
Angola	0.936 (0.743 to 1.119)	1.098 (0.905 to 1.291)	-7.149 (-8.854 to -5.412)
Antigua and Barbuda	0.994 (0.923 to 1.084)	1.364 (1.215 to 1.514)	-4.758 (-11.052 to 1.981)
Argentina	3.781 (3.463 to 4.052)	0.780 (0.381 to 1.181)	-9.151 (-9.893 to -8.403)
Armenia	2.102 (1.853 to 2.329)	-1.521 (-2.188 to -0.849)	-8.302 (-30.825 to 21.554)
Australia	5.412 (4.736 to 5.883)	0.420 (0.226 to 0.614)	-0.851 (-1.335 to -0.365)
Austria	3.675 (3.232 to 3.939)	1.808 (1.516 to 2.101)	-5.396 (-8.362 to -2.335)
Azerbaijan	1.362 (1.076 to 1.826)	-1.251 (-1.532 to -0.970)	-14.462 (-26.562 to -0.369)
Bahamas	0.943 (0.848 to 1.054)	1.106 (0.855 to 1.358)	-10.430 (-17.381 to -2.893)
Bahrain	2.299 (2.016 to 2.599)	-0.625 (-0.918 to -0.330)	-14.013 (-16.623 to -11.322)
Bangladesh	6.482 (5.414 to 8.016)	2.462 (2.216 to 2.709)	-7.155 (-13.741 to -0.067)
Barbados	1.197 (1.064 to 1.292)	0.771 (0.594 to 0.949)	-3.499 (-7.168 to 0.315)
Belarus	0.779 (0.689 to 0.879)	-6.553 (-6.994 to -6.109)	-13.425 (-15.273 to -11.537)
Belgium	5.305 (4.511 to 5.732)	-0.122 (-0.325 to 0.081)	-2.650 (-17.373 to 14.695)
Belize	2.271 (2.08 to 2.503)	1.861 (1.501 to 2.224)	-8.222 (-11.883 to -4.410)
Benin	0.938 (0.716 to 1.211)	0.473 (0.124 to 0.824)	-4.676 (-8.017 to -1.214)
Bermuda	1.816 (1.61 to 2.032)	1.372 (1.073 to 1.671)	-10.104 (-18.529 to -0.806)
Bhutan	10.509 (8.142 to 13.58)	3.703 (3.421 to 3.985)	-0.827 (-2.386 to 0.756)
Bolivia (Plurinational State of)	1.493 (1.227 to 1.782)	1.806 (1.209 to 2.407)	-26.328 (-38.483 to -11.772)

Bosnia and Herzegovina	2.278 (1.957 to 2.633)	-0.857 (-1.149 to -0.563)	-11.211 (-12.118 to -10.294)
Botswana	1.382 (1.128 to 1.735)	0.021 (-0.724 to 0.772)	-18.647 (-32.785 to -1.535)
Brazil	3.544 (3.157 to 3.765)	-0.012 (-0.359 to 0.337)	-11.608 (-11.767 to -11.448)
Brunei Darussalam	3.75 (3.24 to 4.323)	-0.534 (-0.634 to -0.435)	-2.310 (-3.740 to -0.858)
Bulgaria	1.328 (1.142 to 1.528)	-1.490 (-1.716 to -1.263)	-15.648 (-17.253 to -14.012)
Burkina Faso	0.614 (0.503 to 0.761)	1.292 (1.084 to 1.500)	-5.056 (-8.804 to -1.153)
Burundi	1.379 (0.983 to 1.71)	1.129 (0.615 to 1.645)	-4.496 (-4.522 to -4.470)
Cabo Verde	1.812 (1.477 to 2.125)	-1.489 (-2.103 to -0.871)	-4.241 (-6.621 to -1.801)
Cambodia	3.162 (2.644 to 3.805)	3.020 (2.838 to 3.203)	-4.200 (-9.789 to 1.735)
Cameroon	0.913 (0.776 to 1.093)	-0.013 (-0.461 to 0.437)	-7.408 (-8.150 to -6.660)
Canada	4.874 (4.222 to 5.278)	0.595 (0.440 to 0.750)	-5.422 (-12.140 to 1.809)
Central African Republic	1.149 (0.695 to 1.63)	0.418 (0.068 to 0.770)	-4.595 (-10.376 to 1.558)
Chad	0.763 (0.538 to 0.92)	0.193 (-0.110 to 0.497)	-3.472 (-7.973 to 1.248)
Chile	3.161 (2.764 to 3.4)	1.148 (0.695 to 1.603)	-10.872 (-17.151 to -4.117)
China	10.992 (9.681 to 12.223)	-1.469 (-1.650 to -1.287)	0.684 (-0.021 to 1.394)
Colombia	4.898 (4.211 to 5.401)	2.164 (1.774 to 2.556)	-14.256 (-16.742 to -11.697)
Comoros	1.757 (1.288 to 2.208)	1.512 (1.323 to 1.701)	-5.952 (-9.786 to -1.956)
Congo	1.457 (1.159 to 1.85)	0.381 (0.065 to 0.698)	-6.761 (-10.118 to -3.279)
Cook Islands	3.73 (3.155 to 4.441)	-0.466 (-0.627 to -0.305)	0.378 (0.110 to 0.647)
Costa Rica	3.645 (3.056 to 4.042)	0.457 (-0.074 to 0.991)	-11.035 (-12.554 to -9.490)
Croatia	3.121 (2.735 to 3.491)	2.345 (2.125 to 2.565)	-8.057 (-8.148 to -7.965)
Cuba	2.832 (2.554 to 3.117)	2.063 (1.610 to 2.518)	-18.078 (-33.739 to 1.286)
Cyprus	4.989 (4.238 to 5.811)	0.400 (0.292 to 0.507)	-2.131 (-2.868 to -1.388)
Czechia	2.816 (2.438 to 3.187)	3.064 (2.414 to 3.718)	-9.378 (-11.688 to -7.008)
C 么 te d'Ivoire	0.96 (0.793 to 1.159)	1.615 (1.181 to 2.051)	-3.215 (-7.383 to 1.141)
Democratic People's Republic of	12.481 (9.653 to 16.726)	0.947 (0.399 to 1.498)	0.095 (-0.492 to 0.685)

Korea			
Democratic Republic of the Congo	1.755 (1.14 to 2.829)	2.018 (1.754 to 2.282)	-4.652 (-4.764 to -4.540)
Denmark	8.494 (7.496 to 9.13)	1.748 (1.542 to 1.953)	-1.199 (-1.758 to -0.637)
Djibouti	0.942 (0.632 to 1.251)	0.767 (0.435 to 1.100)	-8.072 (-13.964 to -1.777)
Dominica	1.818 (1.464 to 2.173)	0.310 (0.109 to 0.511)	-9.838 (-20.394 to 2.119)
Dominican Republic	1.718 (1.257 to 2.578)	1.832 (1.512 to 2.154)	-3.291 (-5.540 to -0.989)
Ecuador	1.955 (1.743 to 2.201)	0.875 (0.431 to 1.322)	-17.122 (-36.732 to 8.567)
Egypt	1.504 (1.326 to 1.722)	-1.155 (-1.490 to -0.819)	-9.935 (-12.532 to -7.261)
El Salvador	2.36 (1.965 to 2.812)	1.039 (0.730 to 1.348)	-11.192 (-11.326 to -11.057)
Equatorial Guinea	0.869 (0.655 to 1.203)	-0.565 (-0.787 to -0.342)	-8.920 (-15.251 to -2.116)
Eritrea	1.393 (1.126 to 1.668)	2.096 (1.863 to 2.330)	-4.761 (-9.546 to 0.276)
Estonia	1.036 (0.9 to 1.169)	1.133 (0.889 to 1.377)	-8.904 (-15.407 to -1.900)
Eswatini	1.348 (0.983 to 1.716)	-0.643 (-1.268 to -0.014)	-18.922 (-23.171 to -14.439)
Ethiopia	1.197 (0.95 to 1.403)	2.317 (2.027 to 2.607)	-10.879 (-11.452 to -10.302)
Fiji	2.142 (1.644 to 2.646)	-1.193 (-1.377 to -1.009)	-9.064 (-17.545 to 0.289)
Finland	2.631 (2.286 to 2.861)	1.251 (1.069 to 1.434)	-1.285 (-1.615 to -0.953)
France	2.582 (2.196 to 2.822)	-0.900 (-1.323 to -0.476)	-4.409 (-9.663 to 1.150)
Gabon	1.315 (1.039 to 1.832)	-0.964 (-1.241 to -0.686)	-9.010 (-13.374 to -4.426)
Gambia	1.382 (1.02 to 1.779)	1.810 (1.544 to 2.077)	-7.403 (-14.416 to 0.184)
Georgia	1.174 (1 to 1.353)	3.670 (2.869 to 4.477)	-14.374 (-17.985 to -10.604)
Germany	4.017 (3.536 to 4.317)	1.260 (1.083 to 1.438)	-3.934 (-4.107 to -3.761)
Ghana	0.713 (0.606 to 0.843)	2.184 (1.904 to 2.464)	-6.709 (-6.827 to -6.591)
Greece	3.477 (2.989 to 3.757)	2.619 (1.818 to 3.426)	-5.165 (-7.052 to -3.239)
Greenland	5.388 (4.554 to 6.225)	0.866 (0.772 to 0.959)	-2.821 (-7.586 to 2.190)
Grenada	1.255 (0.992 to 1.427)	0.557 (0.186 to 0.930)	-13.296 (-27.462 to 3.638)
Guam	2.555 (2.168 to 2.952)	-0.836 (-1.125 to -0.546)	-10.844 (-17.264 to -3.926)

Guatemala	1.419 (1.283 to 1.555)	2.473 (2.089 to 2.859)	-15.323 (-17.775 to -12.797)
Guinea	1.053 (0.798 to 1.294)	1.021 (0.842 to 1.201)	-7.131 (-11.884 to -2.122)
Guinea-Bissau	1.065 (0.747 to 1.331)	1.076 (0.678 to 1.476)	-6.952 (-16.155 to 3.262)
Guyana	0.916 (0.649 to 1.122)	2.314 (1.993 to 2.636)	-13.433 (-16.851 to -9.874)
Haiti	1.443 (0.639 to 2.174)	1.416 (0.593 to 2.246)	-10.701 (-11.408 to -9.988)
Honduras	3.798 (3.047 to 4.715)	1.972 (1.541 to 2.405)	-14.049 (-18.156 to -9.736)
Hungary	3.879 (3.382 to 4.386)	1.581 (1.123 to 2.042)	-10.066 (-11.623 to -8.481)
Iceland	4.98 (4.185 to 5.443)	1.340 (1.184 to 1.495)	-1.572 (-4.107 to 1.031)
India	9.076 (8.063 to 9.974)	2.540 (2.325 to 2.755)	-9.769 (-10.530 to -9.002)
Indonesia	3.897 (3.223 to 4.635)	1.303 (1.090 to 1.516)	-7.303 (-8.953 to -5.623)
Iran (Islamic Republic of)	2.01 (1.744 to 2.205)	2.742 (2.320 to 3.166)	-19.694 (-25.868 to -13.005)
Iraq	0.862 (0.699 to 1.042)	0.511 (0.190 to 0.832)	-7.447 (-19.482 to 6.387)
Ireland	6.212 (5.317 to 6.857)	-0.188 (-0.400 to 0.024)	-5.256 (-5.894 to -4.613)
Israel	3.324 (2.842 to 3.614)	0.383 (0.141 to 0.627)	-4.257 (-5.833 to -2.654)
Italy	4.092 (3.369 to 4.484)	0.672 (0.519 to 0.826)	-4.052 (-11.955 to 4.560)
Jamaica	1.955 (1.716 to 2.229)	0.695 (0.109 to 1.284)	-9.998 (-17.630 to -1.658)
Japan	2.28 (1.849 to 2.498)	-0.066 (-0.263 to 0.133)	0.509 (0.170 to 0.848)
Jordan	1.233 (1.045 to 1.461)	0.132 (-0.169 to 0.434)	-17.116 (-22.850 to -10.956)
Kazakhstan	3.938 (3.408 to 4.533)	0.924 (0.057 to 1.798)	-14.288 (-18.447 to -9.917)
Kenya	1.631 (1.024 to 2.86)	2.658 (2.151 to 3.167)	-10.178 (-14.461 to -5.679)
Kiribati	4.044 (2.961 to 6.422)	0.044 (-0.053 to 0.142)	0.972 (0.661 to 1.284)
Kuwait	0.469 (0.401 to 0.525)	-0.343 (-0.984 to 0.302)	-8.749 (-20.700 to 5.002)
Kyrgyzstan	2.743 (2.472 to 3.025)	-3.392 (-3.830 to -2.952)	-8.389 (-19.134 to 3.784)
Lao People's Democratic Republic	3.822 (2.888 to 4.782)	2.028 (1.930 to 2.125)	-3.453 (-9.140 to 2.590)
Latvia	0.875 (0.743 to 1.022)	-0.204 (-0.578 to 0.171)	-11.313 (-19.342 to -2.485)
Lebanon	2.638 (2.291 to 3.027)	1.107 (0.666 to 1.550)	-19.578 (-19.621 to -19.536)

Lesotho	1.635 (1.144 to 2.072)	-0.819 (-1.252 to -0.384)	-16.641 (-23.796 to -8.814)
Liberia	1.066 (0.842 to 1.388)	1.156 (0.845 to 1.469)	-3.451 (-9.407 to 2.897)
Libya	1.836 (1.494 to 2.266)	0.482 (0.173 to 0.792)	-8.196 (-11.981 to -4.248)
Lithuania	1.206 (1.072 to 1.332)	-2.652 (-2.811 to -2.493)	-10.256 (-11.232 to -9.269)
Luxembourg	4.876 (4.346 to 5.295)	1.052 (0.918 to 1.185)	-4.528 (-10.610 to 1.967)
Madagascar	1.906 (1.533 to 2.376)	0.685 (0.540 to 0.831)	-5.440 (-9.568 to -1.124)
Malawi	0.973 (0.819 to 1.171)	2.948 (2.645 to 3.251)	-12.082 (-20.117 to -3.238)
Malaysia	3.023 (2.644 to 3.423)	-0.950 (-1.174 to -0.724)	-7.100 (-12.748 to -1.087)
Maldives	5.657 (4.847 to 6.534)	1.233 (0.812 to 1.657)	-7.726 (-11.053 to -4.275)
Mali	1.322 (1.042 to 1.706)	1.640 (1.389 to 1.891)	-7.527 (-10.140 to -4.837)
Malta	2.732 (2.382 to 3.001)	-0.485 (-0.712 to -0.258)	-5.547 (-8.702 to -2.282)
Marshall Islands	3.549 (2.701 to 4.271)	-0.584 (-0.865 to -0.303)	0.857 (0.840 to 0.875)
Mauritania	1.348 (1.135 to 1.616)	0.605 (0.213 to 0.999)	-4.172 (-12.938 to 5.476)
Mauritius	2.095 (1.875 to 2.302)	-0.504 (-0.718 to -0.290)	-4.178 (-9.367 to 1.309)
Mexico	2.755 (2.458 to 3.041)	0.428 (-0.071 to 0.930)	-16.832 (-32.775 to 2.893)
Micronesia (Federated States of)	4.169 (3.498 to 4.967)	-1.046 (-1.155 to -0.938)	0.161 (0.013 to 0.309)
Monaco	2.485 (2.017 to 3.03)	0.296 (0.174 to 0.418)	-7.293 (-11.573 to -2.805)
Mongolia	1.568 (1.265 to 1.875)	-1.171 (-1.491 to -0.849)	-3.036 (-7.867 to 2.048)
Montenegro	0.536 (0.432 to 0.642)	-0.367 (-0.595 to -0.138)	-14.926 (-20.275 to -9.219)
Morocco	2.347 (1.987 to 2.794)	2.086 (1.887 to 2.286)	-7.552 (-18.021 to 4.253)
Mozambique	0.762 (0.629 to 0.913)	1.442 (1.276 to 1.609)	-8.914 (-14.206 to -3.295)
Myanmar	7.875 (6.298 to 9.16)	1.931 (1.711 to 2.151)	-5.834 (-9.193 to -2.350)
Namibia	2.092 (1.761 to 2.594)	0.207 (-0.279 to 0.695)	-16.690 (-25.951 to -6.271)
Nauru	3.342 (2.585 to 5.255)	-0.613 (-0.752 to -0.474)	0.347 (0.338 to 0.357)
Nepal	10.894 (9.024 to 12.45)	3.137 (2.842 to 3.432)	-12.203 (-12.839 to -11.562)
Netherlands	6.321 (5.488 to 6.818)	0.745 (0.640 to 0.851)	-3.447 (-7.944 to 1.271)

New Zealand	5.892 (5.12 to 6.367)	0.259 (0.049 to 0.470)	0.443 (0.049 to 0.838)
Nicaragua	1.951 (1.612 to 2.228)	2.920 (2.128 to 3.720)	-23.998 (-33.378 to -13.298)
Niger	0.824 (0.56 to 1.018)	2.837 (2.425 to 3.251)	-2.967 (-5.067 to -0.819)
Nigeria	0.691 (0.586 to 0.813)	0.434 (0.196 to 0.673)	-3.086 (-4.363 to -1.792)
Niue	4.499 (3.791 to 5.363)	-1.319 (-1.379 to -1.259)	-3.541 (-6.664 to -0.313)
North Macedonia	1.708 (1.347 to 2.411)	-0.564 (-0.799 to -0.329)	-16.098 (-18.203 to -13.939)
Northern Mariana Islands	4.145 (3.517 to 4.894)	0.301 (0.192 to 0.411)	-3.639 (-6.854 to -0.312)
Norway	6.785 (5.907 to 7.255)	3.925 (3.515 to 4.337)	-0.368 (-0.771 to 0.036)
Oman	1.075 (0.915 to 1.325)	0.854 (0.549 to 1.159)	-16.808 (-20.518 to -12.924)
Pakistan	3.745 (3.142 to 4.562)	0.115 (-0.106 to 0.337)	-8.947 (-15.382 to -2.022)
Palau	5.081 (4.553 to 5.782)	0.002 (-0.052 to 0.056)	0.863 (0.671 to 1.056)
Palestine	1.477 (1.295 to 1.732)	0.063 (-0.315 to 0.442)	-9.184 (-12.213 to -6.050)
Panama	2.994 (2.593 to 3.279)	0.613 (0.196 to 1.032)	-8.304 (-21.105 to 6.573)
Papua New Guinea	6.455 (4.981 to 7.956)	0.279 (0.151 to 0.407)	-8.630 (-16.709 to 0.234)
Paraguay	1.944 (1.712 to 2.201)	1.024 (0.739 to 1.309)	-17.416 (-27.349 to -6.125)
Peru	0.982 (0.737 to 1.287)	2.014 (1.256 to 2.777)	-29.765 (-36.965 to -21.743)
Philippines	2.481 (2.257 to 2.741)	0.489 (0.243 to 0.736)	-13.052 (-26.942 to 3.480)
Poland	1.764 (1.596 to 1.915)	-0.520 (-0.780 to -0.259)	-9.848 (-12.960 to -6.626)
Portugal	4.631 (3.878 to 5.111)	1.091 (0.772 to 1.411)	-4.034 (-7.567 to -0.367)
Puerto Rico	4.557 (3.889 to 5.008)	0.996 (0.645 to 1.349)	-3.861 (-6.598 to -1.042)
Qatar	1.006 (0.879 to 1.16)	-0.557 (-0.763 to -0.350)	-4.967 (-7.135 to -2.748)
Republic of Korea	3.298 (2.689 to 4.072)	1.406 (1.178 to 1.636)	-0.025 (-2.153 to 2.149)
Republic of Moldova	1.385 (1.27 to 1.511)	-3.190 (-3.653 to -2.724)	-10.831 (-10.935 to -10.727)
Romania	2.054 (1.805 to 2.286)	-2.063 (-2.393 to -1.732)	-11.418 (-12.141 to -10.689)
Russian Federation	1.312 (1.206 to 1.422)	-1.995 (-2.431 to -1.557)	-14.837 (-15.562 to -14.105)
Rwanda	1.737 (1.293 to 2.219)	2.598 (1.473 to 3.735)	-8.712 (-18.061 to 1.702)

Saint Kitts and Nevis	1.658 (1.5 to 1.836)	1.118 (0.897 to 1.339)	-6.647 (-13.359 to 0.586)
Saint Lucia	2.811 (2.265 to 3.229)	1.423 (1.123 to 1.724)	-13.376 (-22.130 to -3.638)
Saint Vincent and the Grenadines	1.262 (1.154 to 1.388)	2.502 (2.103 to 2.902)	-4.048 (-8.484 to 0.602)
Samoa	5.986 (4.688 to 7.216)	-0.495 (-0.598 to -0.392)	2.917 (0.977 to 4.894)
San Marino	2.414 (1.856 to 2.924)	0.449 (0.173 to 0.727)	-11.997 (-28.979 to 9.045)
Sao Tome and Principe	4.548 (3.48 to 5.646)	1.707 (1.542 to 1.872)	-5.848 (-8.503 to -3.115)
Saudi Arabia	1.802 (1.562 to 2.08)	-0.147 (-0.288 to -0.004)	-3.545 (-10.031 to 3.408)
Senegal	1.451 (1.21 to 1.738)	2.256 (1.734 to 2.781)	-8.880 (-10.335 to -7.401)
Serbia	2.317 (1.942 to 2.706)	0.036 (-0.158 to 0.230)	-9.886 (-11.453 to -8.291)
Seychelles	2.688 (2.373 to 3.042)	-0.856 (-0.962 to -0.749)	-6.131 (-7.506 to -4.735)
Sierra Leone	0.976 (0.75 to 1.283)	0.990 (0.648 to 1.333)	-2.565 (-9.067 to 4.402)
Singapore	2.057 (1.814 to 2.215)	-3.012 (-3.146 to -2.877)	-1.294 (-6.474 to 4.173)
Slovakia	1.287 (1.085 to 1.544)	0.673 (0.437 to 0.909)	-12.598 (-18.612 to -6.140)
Slovenia	2.644 (2.317 to 2.897)	-1.111 (-1.334 to -0.888)	-6.036 (-12.317 to 0.696)
Solomon Islands	3.43 (3 to 3.889)	0.479 (0.383 to 0.575)	-0.169 (-0.327 to -0.010)
Somalia	0.779 (0.46 to 1.035)	0.294 (-0.251 to 0.842)	-9.021 (-10.151 to -7.877)
South Africa	1.816 (1.681 to 1.97)	-0.162 (-0.721 to 0.400)	-15.317 (-20.475 to -9.824)
South Sudan	0.929 (0.645 to 1.182)	-0.814 (-1.098 to -0.528)	-5.871 (-8.879 to -2.764)
Spain	6.655 (5.607 to 7.324)	0.428 (0.242 to 0.614)	-4.394 (-13.418 to 5.571)
Sri Lanka	6.766 (5.553 to 8.119)	1.007 (0.769 to 1.245)	-6.270 (-10.410 to -1.938)
Sudan	1.635 (1.102 to 2.159)	1.612 (1.443 to 1.782)	-8.504 (-14.935 to -1.586)
Suriname	1.691 (1.357 to 1.981)	0.786 (0.568 to 1.005)	-12.907 (-23.461 to -0.898)
Sweden	3.915 (3.389 to 4.234)	2.521 (2.209 to 2.834)	-2.692 (-9.947 to 5.146)
Switzerland	3.58 (3.016 to 3.95)	0.326 (0.215 to 0.438)	-2.401 (-11.896 to 8.118)
Syrian Arab Republic	2.38 (2.042 to 2.744)	0.237 (-0.452 to 0.931)	-0.218 (-5.309 to 5.146)
Taiwan (Province of China)	4.235 (3.656 to 4.651)	0.969 (0.712 to 1.227)	-0.484 (-1.381 to 0.422)

Tajikistan	2.076 (1.539 to 2.743)	-0.265 (-0.621 to 0.092)	-12.190 (-20.418 to -3.111)
Thailand	3.421 (2.845 to 4.004)	-1.299 (-1.447 to -1.151)	-2.669 (-7.857 to 2.811)
Timor-Leste	3.718 (2.847 to 4.572)	4.104 (3.663 to 4.547)	-5.606 (-12.973 to 2.385)
Togo	1.264 (1.008 to 1.566)	1.800 (1.355 to 2.247)	-2.413 (-3.045 to -1.777)
Tokelau	4.701 (3.631 to 6.986)	-0.305 (-0.445 to -0.165)	-6.015 (-12.517 to 0.971)
Tonga	5.507 (4.869 to 6.255)	0.013 (-0.101 to 0.127)	-0.184 (-0.400 to 0.032)
Trinidad and Tobago	1.224 (0.963 to 1.409)	0.790 (0.564 to 1.016)	-13.033 (-20.444 to -4.931)
Tunisia	1.952 (1.5 to 2.642)	1.353 (0.994 to 1.713)	-17.839 (-25.863 to -8.947)
Turkmenistan	0.883 (0.768 to 1)	-3.081 (-3.646 to -2.512)	-9.014 (-14.620 to -3.040)
Tuvalu	4.112 (3.23 to 5.13)	-0.386 (-0.547 to -0.225)	0.180 (0.045 to 0.316)
T 眉 rkiye	5.2 (4.579 to 5.851)	1.662 (1.327 to 1.999)	-11.561 (-17.833 to -4.810)
Uganda	0.996 (0.688 to 1.329)	1.499 (1.178 to 1.822)	-10.835 (-15.477 to -5.938)
Ukraine	1.001 (0.875 to 1.144)	-5.751 (-6.024 to -5.478)	-9.947 (-14.232 to -5.449)
United Arab Emirates	2.139 (1.821 to 2.603)	-0.022 (-0.192 to 0.149)	-10.367 (-13.083 to -7.565)
United Kingdom	5.739 (5.068 to 6.066)	1.036 (0.765 to 1.308)	-7.830 (-14.419 to -0.735)
United Republic of Tanzania	0.998 (0.796 to 1.231)	1.753 (1.474 to 2.032)	-8.138 (-9.244 to -7.019)
United States Virgin Islands	1.454 (1.044 to 1.932)	0.820 (0.668 to 0.973)	-8.421 (-9.731 to -7.092)
United States of America	5.705 (4.978 to 6.045)	1.432 (1.148 to 1.718)	-7.427 (-14.581 to 0.326)
Uruguay	5.109 (4.653 to 5.42)	1.177 (0.963 to 1.391)	-6.885 (-14.097 to 0.933)
Uzbekistan	0.869 (0.759 to 0.989)	-3.620 (-4.440 to -2.793)	-4.008 (-10.728 to 3.217)
Vanuatu	4.481 (2.774 to 5.913)	-0.066 (-0.210 to 0.079)	-5.181 (-10.923 to 0.931)
Venezuela (Bolivarian Republic of)	2.197 (1.916 to 2.431)	1.369 (0.870 to 1.871)	-13.888 (-21.960 to -4.981)
Viet Nam	5.047 (4.153 to 6.14)	0.508 (0.314 to 0.703)	-3.156 (-6.047 to -0.175)
Yemen	1.558 (1.16 to 1.886)	2.082 (1.827 to 2.337)	-3.880 (-5.518 to -2.213)
Zambia	0.878 (0.732 to 1.051)	2.379 (1.918 to 2.842)	-14.189 (-19.546 to -8.474)
Zimbabwe	0.756 (0.587 to 0.917)	0.788 (0.248 to 1.331)	-15.447 (-22.004 to -8.339)

EAPC: Estimated Annual Percentage Change; COPD: Chronic Obstructive Pulmonary Disease; EAPCs for ASRs from 1990 to 2019 (%, 95% UI) were calculated using GBD 2021.

Table S6. The percent in all-cause DALYs and EAPC for percent of COPD at national level, 1990-2021 and 2019-2021.

location	Percent, in 2021 (95% UI)	EAPC for percent from 1990 to 2021 (95% CI)	EAPC for percent from 2019 to 2021 (95% CI)
Afghanistan	0.662 (0.509 to 0.838)	-0.388 (-0.616 to -0.159)	-4.901 (-6.624 to -3.147)
Albania	1.587 (1.285 to 2.028)	0.853 (0.675 to 1.032)	-7.365 (-11.053 to -3.524)
Algeria	1.250 (1.067 to 1.458)	2.619 (2.411 to 2.827)	-6.591 (-10.061 to -2.987)
American Samoa	2.532 (2.251 to 2.848)	-0.106 (-0.243 to 0.031)	1.137 (0.863 to 1.411)
Andorra	2.634 (2.114 to 3.175)	-0.006 (-0.190 to 0.180)	-6.013 (-13.103 to 1.655)
Angola	0.486 (0.412 to 0.556)	1.743 (1.511 to 1.975)	-1.976 (-3.457 to -0.472)
Antigua and Barbuda	0.652 (0.576 to 0.727)	1.541 (1.350 to 1.732)	-1.390 (-2.866 to 0.108)
Argentina	2.014 (1.764 to 2.244)	0.838 (0.540 to 1.137)	-6.809 (-7.273 to -6.343)
Armenia	1.415 (1.257 to 1.588)	-1.009 (-1.603 to -0.412)	-5.869 (-32.555 to 31.376)
Australia	2.608 (2.197 to 3.022)	0.068 (-0.118 to 0.254)	-0.458 (-2.204 to 1.319)
Austria	2.608 (2.254 to 2.979)	1.762 (1.576 to 1.949)	-4.050 (-5.575 to -2.500)
Azerbaijan	0.958 (0.772 to 1.274)	-0.202 (-0.418 to 0.016)	-8.535 (-18.970 to 3.244)
Bahamas	0.606 (0.534 to 0.680)	1.672 (1.508 to 1.837)	-3.257 (-10.670 to 4.770)
Bahrain	1.268 (1.088 to 1.451)	-0.433 (-0.673 to -0.193)	-6.580 (-7.623 to -5.524)
Bangladesh	3.345 (2.774 to 3.929)	3.715 (3.559 to 3.872)	-2.136 (-5.471 to 1.317)
Barbados	0.812 (0.695 to 0.906)	1.151 (1.049 to 1.252)	-2.653 (-5.483 to 0.263)
Belarus	0.808 (0.715 to 0.907)	-4.720 (-5.054 to -4.384)	-10.107 (-11.024 to -9.181)
Belgium	3.283 (2.804 to 3.731)	-0.043 (-0.179 to 0.094)	-2.422 (-12.295 to 8.562)
Belize	1.076 (0.942 to 1.216)	2.543 (2.167 to 2.921)	-3.522 (-6.545 to -0.402)
Benin	0.460 (0.369 to 0.571)	1.195 (0.871 to 1.520)	0.331 (-1.752 to 2.458)
Bermuda	1.246 (1.073 to 1.413)	1.617 (1.427 to 1.808)	-5.873 (-10.376 to -1.144)
Bhutan	4.475 (3.507 to 5.556)	4.225 (4.000 to 4.450)	1.155 (0.595 to 1.718)
Bolivia (Plurinational State of)	0.775 (0.645 to 0.914)	2.635 (2.238 to 3.034)	-16.498 (-24.631 to -7.488)

Bosnia and Herzegovina	1.826 (1.590 to 2.096)	0.289 (-0.087 to 0.666)	-7.998 (-8.428 to -7.566)
Botswana	0.868 (0.731 to 1.046)	0.664 (-0.030 to 1.364)	-9.757 (-19.671 to 1.379)
Brazil	1.760 (1.550 to 1.958)	0.713 (0.440 to 0.987)	-6.588 (-7.447 to -5.722)
Brunei Darussalam	1.593 (1.331 to 1.881)	-0.175 (-0.269 to -0.081)	-0.444 (-2.486 to 1.641)
Bulgaria	1.277 (1.118 to 1.462)	-0.754 (-0.935 to -0.571)	-12.456 (-14.557 to -10.302)
Burkina Faso	0.322 (0.269 to 0.384)	1.823 (1.582 to 2.065)	-0.494 (-3.764 to 2.887)
Burundi	0.663 (0.496 to 0.820)	1.638 (1.113 to 2.165)	-0.746 (-1.205 to -0.284)
Cabo Verde	1.091 (0.933 to 1.270)	0.232 (-0.364 to 0.831)	-1.933 (-3.456 to -0.386)
Cambodia	1.708 (1.465 to 1.997)	4.072 (3.909 to 4.236)	-1.242 (-4.751 to 2.397)
Cameroon	0.508 (0.439 to 0.595)	0.805 (0.409 to 1.203)	-1.486 (-2.064 to -0.905)
Canada	2.731 (2.356 to 3.124)	0.637 (0.498 to 0.776)	-3.852 (-8.336 to 0.852)
Central African Republic	0.598 (0.406 to 0.791)	0.923 (0.547 to 1.300)	-0.721 (-3.462 to 2.098)
Chad	0.344 (0.263 to 0.402)	0.684 (0.410 to 0.960)	-0.306 (-2.182 to 1.607)
Chile	1.372 (1.173 to 1.568)	0.917 (0.600 to 1.234)	-6.956 (-11.410 to -2.279)
China	5.882 (5.092 to 6.661)	-0.721 (-0.904 to -0.537)	1.420 (0.804 to 2.040)
Colombia	2.160 (1.857 to 2.470)	2.253 (2.002 to 2.504)	-4.590 (-4.858 to -4.321)
Comoros	0.978 (0.757 to 1.192)	2.411 (2.195 to 2.628)	-0.636 (-4.624 to 3.518)
Congo	0.837 (0.696 to 1.025)	1.007 (0.615 to 1.401)	-1.676 (-3.255 to -0.070)
Cook Islands	2.269 (1.948 to 2.663)	0.122 (-0.049 to 0.293)	-0.008 (-0.241 to 0.226)
Costa Rica	1.566 (1.338 to 1.796)	0.798 (0.402 to 1.195)	-5.907 (-6.889 to -4.914)
Croatia	2.267 (1.976 to 2.553)	2.171 (1.986 to 2.357)	-5.566 (-6.200 to -4.928)
Cuba	2.059 (1.834 to 2.301)	2.561 (2.199 to 2.925)	-12.238 (-23.567 to 0.771)
Cyprus	2.566 (2.135 to 3.036)	0.044 (-0.072 to 0.160)	-1.950 (-2.089 to -1.812)
Czechia	2.222 (1.923 to 2.551)	2.633 (2.177 to 3.092)	-6.612 (-7.113 to -6.108)
C 么 te d'Ivoire	0.521 (0.443 to 0.623)	2.099 (1.720 to 2.480)	1.061 (-1.300 to 3.479)
Democratic People's Republic of	6.799 (5.403 to 8.591)	1.838 (1.131 to 2.551)	0.210 (-0.234 to 0.656)

Korea			
Democratic Republic of the Congo	0.853 (0.605 to 1.221)	2.570 (2.222 to 2.919)	0.948 (0.412 to 1.487)
Denmark	4.877 (4.172 to 5.563)	1.004 (0.824 to 1.184)	-0.908 (-1.999 to 0.195)
Djibouti	0.606 (0.466 to 0.759)	1.819 (1.513 to 2.126)	-2.342 (-5.431 to 0.848)
Dominica	1.123 (0.947 to 1.304)	0.677 (0.550 to 0.803)	-6.046 (-13.387 to 1.917)
Dominican Republic	0.846 (0.655 to 1.173)	3.014 (2.734 to 3.296)	0.249 (-3.695 to 4.355)
Ecuador	0.858 (0.748 to 0.981)	1.616 (1.299 to 1.934)	-9.327 (-22.379 to 5.919)
Egypt	1.058 (0.928 to 1.194)	0.109 (-0.222 to 0.442)	-5.134 (-6.380 to -3.872)
El Salvador	1.108 (0.937 to 1.284)	2.010 (1.746 to 2.274)	-5.506 (-6.683 to -4.314)
Equatorial Guinea	0.469 (0.380 to 0.601)	0.203 (-0.095 to 0.502)	-3.175 (-6.796 to 0.587)
Eritrea	0.769 (0.643 to 0.911)	2.499 (2.292 to 2.706)	-0.880 (-3.980 to 2.321)
Estonia	0.889 (0.783 to 1.010)	1.397 (1.197 to 1.597)	-7.115 (-10.892 to -3.178)
Eswatini	0.830 (0.649 to 1.014)	0.363 (-0.140 to 0.868)	-11.252 (-15.170 to -7.153)
Ethiopia	0.607 (0.490 to 0.711)	2.471 (2.200 to 2.744)	-4.181 (-5.064 to -3.289)
Fiji	1.350 (1.088 to 1.606)	-0.567 (-0.689 to -0.445)	-5.843 (-12.180 to 0.952)
Finland	1.951 (1.680 to 2.220)	1.209 (1.086 to 1.332)	-0.455 (-1.530 to 0.631)
France	1.587 (1.346 to 1.819)	-0.262 (-0.625 to 0.102)	-2.911 (-6.614 to 0.939)
Gabon	0.781 (0.651 to 1.013)	-0.174 (-0.514 to 0.166)	-3.478 (-5.762 to -1.140)
Gambia	0.742 (0.582 to 0.913)	2.489 (2.264 to 2.715)	-2.421 (-6.007 to 1.302)
Georgia	1.040 (0.907 to 1.182)	3.348 (2.796 to 3.903)	-9.528 (-13.255 to -5.642)
Germany	2.946 (2.552 to 3.321)	1.370 (1.217 to 1.524)	-2.723 (-2.815 to -2.632)
Ghana	0.513 (0.449 to 0.590)	2.834 (2.674 to 2.993)	-1.175 (-1.343 to -1.007)
Greece	2.664 (2.322 to 3.022)	2.384 (1.861 to 2.908)	-3.357 (-4.514 to -2.186)
Greenland	2.957 (2.466 to 3.497)	1.455 (1.383 to 1.527)	-1.888 (-5.762 to 2.146)
Grenada	0.840 (0.713 to 0.949)	1.161 (0.931 to 1.392)	-8.763 (-18.992 to 2.757)
Guam	1.704 (1.486 to 1.945)	0.004 (-0.233 to 0.242)	-5.106 (-10.562 to 0.683)

Guatemala	0.664 (0.587 to 0.732)	2.834 (2.547 to 3.121)	-6.005 (-7.393 to -4.596)
Guinea	0.521 (0.419 to 0.614)	1.645 (1.535 to 1.755)	-1.353 (-4.047 to 1.418)
Guinea-Bissau	0.607 (0.463 to 0.733)	1.959 (1.573 to 2.347)	-2.269 (-7.687 to 3.467)
Guyana	0.577 (0.461 to 0.686)	3.371 (3.123 to 3.619)	-7.351 (-10.003 to -4.620)
Haiti	0.669 (0.322 to 0.981)	1.765 (0.909 to 2.629)	-5.399 (-6.027 to -4.767)
Honduras	1.859 (1.527 to 2.334)	3.070 (2.697 to 3.444)	-8.210 (-10.394 to -5.972)
Hungary	3.176 (2.763 to 3.592)	2.024 (1.653 to 2.396)	-7.935 (-9.831 to -5.998)
Iceland	2.885 (2.408 to 3.323)	0.975 (0.796 to 1.154)	-0.929 (-3.848 to 2.080)
India	4.537 (3.978 to 5.062)	3.099 (2.968 to 3.230)	-5.240 (-5.410 to -5.071)
Indonesia	2.356 (2.009 to 2.732)	2.301 (2.127 to 2.475)	-4.071 (-5.577 to -2.540)
Iran (Islamic Republic of)	1.263 (1.114 to 1.421)	3.363 (3.084 to 3.643)	-10.686 (-15.551 to -5.540)
Iraq	0.605 (0.514 to 0.694)	1.599 (1.265 to 1.934)	-1.626 (-7.484 to 4.603)
Ireland	3.014 (2.521 to 3.533)	-0.829 (-1.125 to -0.531)	-4.075 (-4.313 to -3.837)
Israel	1.894 (1.609 to 2.197)	0.462 (0.282 to 0.642)	-2.734 (-3.504 to -1.958)
Italy	2.462 (2.055 to 2.822)	0.732 (0.622 to 0.843)	-2.826 (-6.777 to 1.293)
Jamaica	1.176 (1.010 to 1.353)	1.334 (0.857 to 1.813)	-5.274 (-8.847 to -1.561)
Japan	1.743 (1.459 to 2.015)	0.774 (0.657 to 0.892)	0.413 (0.087 to 0.740)
Jordan	0.825 (0.701 to 0.960)	0.925 (0.757 to 1.093)	-6.706 (-8.402 to -4.978)
Kazakhstan	2.515 (2.163 to 2.929)	1.081 (0.315 to 1.853)	-9.478 (-12.138 to -6.737)
Kenya	0.874 (0.607 to 1.379)	3.506 (3.070 to 3.943)	-4.040 (-5.738 to -2.311)
Kiribati	2.165 (1.637 to 3.298)	0.619 (0.524 to 0.715)	1.272 (1.130 to 1.414)
Kuwait	0.569 (0.476 to 0.675)	1.089 (0.723 to 1.456)	-2.297 (-9.327 to 5.279)
Kyrgyzstan	1.478 (1.296 to 1.673)	-2.978 (-3.444 to -2.510)	-4.463 (-10.840 to 2.371)
Lao People's Democratic Republic	1.906 (1.506 to 2.307)	2.806 (2.731 to 2.881)	-0.251 (-3.753 to 3.379)
Latvia	0.883 (0.760 to 1.011)	0.702 (0.387 to 1.019)	-7.984 (-14.923 to -0.479)
Lebanon	1.718 (1.491 to 1.986)	1.591 (1.241 to 1.943)	-12.143 (-13.288 to -10.982)

Lesotho	0.980 (0.734 to 1.200)	0.111 (-0.238 to 0.460)	-10.364 (-15.637 to -4.763)
Liberia	0.542 (0.459 to 0.668)	2.215 (1.904 to 2.526)	1.530 (-2.818 to 6.072)
Libya	1.157 (0.967 to 1.399)	1.444 (1.155 to 1.734)	-2.120 (-4.567 to 0.389)
Lithuania	1.072 (0.945 to 1.202)	-1.615 (-1.729 to -1.501)	-7.029 (-7.175 to -6.883)
Luxembourg	2.689 (2.297 to 3.096)	0.600 (0.507 to 0.693)	-3.822 (-8.116 to 0.672)
Madagascar	0.946 (0.753 to 1.176)	1.472 (1.276 to 1.669)	-0.096 (-1.740 to 1.576)
Malawi	0.545 (0.474 to 0.642)	3.796 (3.553 to 4.040)	-4.664 (-9.687 to 0.638)
Malaysia	1.826 (1.606 to 2.064)	0.072 (-0.087 to 0.232)	-3.409 (-6.598 to -0.112)
Maldives	2.253 (1.866 to 2.624)	0.930 (0.487 to 1.374)	-3.686 (-4.485 to -2.880)
Mali	0.588 (0.464 to 0.755)	1.831 (1.613 to 2.050)	-1.751 (-3.829 to 0.372)
Malta	1.894 (1.617 to 2.183)	-0.054 (-0.229 to 0.121)	-2.974 (-6.355 to 0.530)
Marshall Islands	2.096 (1.648 to 2.499)	0.206 (-0.080 to 0.493)	0.987 (0.753 to 1.223)
Mauritania	0.743 (0.633 to 0.865)	1.258 (0.835 to 1.683)	0.812 (-3.866 to 5.717)
Mauritius	1.364 (1.212 to 1.509)	0.138 (-0.017 to 0.294)	-1.116 (-2.767 to 0.562)
Mexico	1.319 (1.169 to 1.474)	1.460 (1.087 to 1.835)	-10.252 (-20.787 to 1.684)
Micronesia (Federated States of)	2.474 (2.117 to 2.917)	-0.229 (-0.364 to -0.095)	0.690 (0.630 to 0.751)
Monaco	1.928 (1.630 to 2.236)	0.113 (0.019 to 0.208)	-5.434 (-8.006 to -2.790)
Mongolia	0.878 (0.724 to 1.033)	-0.198 (-0.457 to 0.062)	-1.274 (-3.280 to 0.772)
Montenegro	0.646 (0.562 to 0.740)	0.652 (0.467 to 0.836)	-10.711 (-13.571 to -7.757)
Morocco	1.519 (1.267 to 1.761)	3.289 (3.150 to 3.429)	-3.892 (-10.509 to 3.214)
Mozambique	0.412 (0.346 to 0.482)	2.321 (2.147 to 2.496)	-3.476 (-5.981 to -0.904)
Myanmar	3.853 (3.150 to 4.434)	2.368 (2.142 to 2.595)	-2.504 (-5.246 to 0.317)
Namibia	1.238 (1.079 to 1.466)	0.772 (0.338 to 1.209)	-9.171 (-16.166 to -1.592)
Nauru	1.861 (1.506 to 2.507)	-0.484 (-0.652 to -0.315)	0.466 (0.296 to 0.636)
Nepal	5.371 (4.404 to 6.327)	4.119 (3.890 to 4.348)	-6.497 (-6.518 to -6.477)
Netherlands	3.817 (3.287 to 4.327)	0.851 (0.753 to 0.950)	-3.282 (-6.245 to -0.225)

New Zealand	2.828 (2.388 to 3.280)	0.097 (-0.084 to 0.277)	-0.065 (-0.804 to 0.680)
Nicaragua	0.941 (0.810 to 1.078)	3.799 (3.209 to 4.392)	-12.122 (-17.076 to -6.872)
Niger	0.386 (0.303 to 0.456)	3.305 (2.927 to 3.685)	0.275 (-0.279 to 0.832)
Nigeria	0.348 (0.296 to 0.403)	0.962 (0.673 to 1.252)	1.680 (1.008 to 2.356)
Niue	2.542 (2.163 to 3.019)	-1.104 (-1.220 to -0.988)	-6.884 (-13.487 to 0.224)
North Macedonia	1.395 (1.132 to 1.884)	0.336 (0.117 to 0.556)	-13.095 (-13.763 to -12.422)
Northern Mariana Islands	2.553 (2.183 to 2.991)	1.118 (0.894 to 1.342)	-1.206 (-6.543 to 4.435)
Norway	3.748 (3.163 to 4.310)	2.894 (2.550 to 3.239)	-0.611 (-1.931 to 0.726)
Oman	0.703 (0.601 to 0.829)	1.073 (0.822 to 1.326)	-8.756 (-11.412 to -6.021)
Pakistan	1.661 (1.400 to 1.961)	0.610 (0.437 to 0.784)	-3.660 (-7.195 to 0.010)
Palau	3.060 (2.723 to 3.477)	0.465 (0.375 to 0.554)	1.556 (1.246 to 1.867)
Palestine	0.824 (0.713 to 0.949)	0.854 (0.386 to 1.325)	-4.343 (-6.962 to -1.649)
Panama	1.270 (1.075 to 1.447)	1.004 (0.718 to 1.291)	-3.268 (-10.242 to 4.248)
Papua New Guinea	2.810 (2.244 to 3.419)	0.388 (0.249 to 0.528)	-4.103 (-9.249 to 1.334)
Paraguay	0.972 (0.835 to 1.140)	1.888 (1.701 to 2.076)	-9.912 (-14.988 to -4.532)
Peru	0.562 (0.451 to 0.688)	2.841 (2.326 to 3.359)	-18.358 (-22.928 to -13.518)
Philippines	1.612 (1.442 to 1.794)	1.744 (1.540 to 1.948)	-7.071 (-15.770 to 2.527)
Poland	1.531 (1.354 to 1.702)	0.085 (-0.078 to 0.249)	-6.365 (-8.124 to -4.572)
Portugal	2.642 (2.242 to 3.030)	0.801 (0.596 to 1.007)	-2.615 (-4.894 to -0.282)
Puerto Rico	2.407 (2.059 to 2.736)	1.425 (1.127 to 1.724)	-1.071 (-3.466 to 1.384)
Qatar	0.653 (0.550 to 0.775)	-0.057 (-0.256 to 0.142)	-2.716 (-3.293 to -2.135)
Republic of Korea	2.064 (1.689 to 2.451)	2.234 (1.983 to 2.485)	-0.114 (-1.143 to 0.926)
Republic of Moldova	1.130 (1.015 to 1.253)	-2.495 (-2.825 to -2.164)	-6.763 (-7.047 to -6.478)
Romania	1.800 (1.590 to 2.005)	-0.921 (-1.208 to -0.634)	-8.295 (-8.352 to -8.238)
Russian Federation	1.100 (1.008 to 1.203)	-1.414 (-1.790 to -1.036)	-10.016 (-10.112 to -9.920)
Rwanda	0.945 (0.761 to 1.181)	3.253 (2.106 to 4.413)	-2.383 (-8.517 to 4.162)

Saint Kitts and Nevis	1.013 (0.895 to 1.146)	1.318 (1.170 to 1.466)	-3.286 (-7.327 to 0.931)
Saint Lucia	1.672 (1.421 to 1.919)	2.034 (1.833 to 2.236)	-7.420 (-13.748 to -0.629)
Saint Vincent and the Grenadines	0.772 (0.695 to 0.859)	3.024 (2.749 to 3.299)	-1.970 (-5.017 to 1.175)
Samoa	3.034 (2.457 to 3.608)	-0.127 (-0.271 to 0.017)	5.679 (1.656 to 9.861)
San Marino	1.685 (1.396 to 2.007)	0.501 (0.310 to 0.693)	-9.122 (-21.935 to 5.795)
Sao Tome and Principe	2.384 (1.962 to 2.942)	2.812 (2.681 to 2.943)	-2.210 (-3.475 to -0.929)
Saudi Arabia	1.068 (0.925 to 1.229)	1.183 (1.021 to 1.345)	-0.956 (-5.156 to 3.431)
Senegal	0.809 (0.683 to 0.947)	3.091 (2.624 to 3.560)	-2.428 (-2.504 to -2.351)
Serbia	1.993 (1.695 to 2.317)	0.722 (0.529 to 0.917)	-7.287 (-8.029 to -6.538)
Seychelles	1.719 (1.508 to 1.954)	-0.470 (-0.566 to -0.374)	-4.128 (-5.510 to -2.726)
Sierra Leone	0.483 (0.395 to 0.603)	1.958 (1.571 to 2.346)	0.988 (-3.681 to 5.883)
Singapore	1.131 (0.945 to 1.305)	-2.443 (-2.615 to -2.270)	0.227 (-2.171 to 2.685)
Slovakia	1.159 (1.000 to 1.349)	1.066 (0.901 to 1.230)	-8.413 (-13.706 to -2.795)
Slovenia	1.878 (1.632 to 2.120)	-0.542 (-0.732 to -0.351)	-4.121 (-7.333 to -0.798)
Solomon Islands	1.802 (1.541 to 2.042)	0.815 (0.708 to 0.923)	0.215 (-0.093 to 0.524)
Somalia	0.411 (0.281 to 0.513)	0.752 (0.216 to 1.290)	-3.749 (-4.045 to -3.453)
South Africa	1.151 (1.058 to 1.254)	0.798 (0.283 to 1.316)	-8.253 (-10.503 to -5.947)
South Sudan	0.410 (0.308 to 0.503)	-0.198 (-0.565 to 0.170)	-1.421 (-2.268 to -0.567)
Spain	3.429 (2.891 to 3.921)	0.249 (0.085 to 0.413)	-3.315 (-8.231 to 1.864)
Sri Lanka	3.423 (2.812 to 4.126)	1.406 (1.165 to 1.648)	-3.363 (-3.799 to -2.925)
Sudan	0.863 (0.633 to 1.089)	2.259 (2.170 to 2.348)	-1.946 (-5.331 to 1.560)
Suriname	0.969 (0.822 to 1.120)	1.508 (1.353 to 1.664)	-8.060 (-15.431 to -0.046)
Sweden	2.770 (2.352 to 3.193)	1.610 (1.332 to 1.888)	-2.209 (-5.857 to 1.581)
Switzerland	2.315 (1.957 to 2.664)	0.361 (0.288 to 0.433)	-2.043 (-6.780 to 2.934)
Syrian Arab Republic	1.616 (1.392 to 1.882)	0.963 (0.139 to 1.794)	7.251 (0.647 to 14.288)
Taiwan (Province of China)	2.247 (1.940 to 2.550)	0.961 (0.781 to 1.141)	0.492 (0.142 to 0.843)

Tajikistan	1.046 (0.816 to 1.339)	0.200 (-0.108 to 0.508)	-4.769 (-8.631 to -0.744)
Thailand	2.066 (1.762 to 2.361)	-0.434 (-0.581 to -0.287)	-0.468 (-4.156 to 3.362)
Timor-Leste	1.764 (1.390 to 2.153)	5.021 (4.602 to 5.441)	-2.624 (-7.067 to 2.032)
Togo	0.720 (0.604 to 0.858)	2.722 (2.336 to 3.110)	1.733 (1.343 to 2.125)
Tokelau	2.328 (1.865 to 3.135)	-0.343 (-0.640 to -0.046)	-12.437 (-24.503 to 1.558)
Tonga	2.724 (2.385 to 3.091)	0.292 (0.159 to 0.425)	0.095 (-0.065 to 0.255)
Trinidad and Tobago	0.814 (0.679 to 0.934)	1.424 (1.252 to 1.596)	-7.047 (-12.683 to -1.047)
Tunisia	1.466 (1.217 to 1.782)	2.368 (2.080 to 2.657)	-10.959 (-17.124 to -4.335)
Turkmenistan	0.649 (0.562 to 0.740)	-1.389 (-1.852 to -0.922)	-4.528 (-7.657 to -1.293)
Tuvalu	2.405 (1.922 to 2.969)	0.086 (-0.093 to 0.266)	0.183 (0.061 to 0.305)
T 眉 rkiye	3.053 (2.620 to 3.522)	2.581 (2.314 to 2.849)	-6.687 (-10.076 to -3.170)
Uganda	0.482 (0.367 to 0.604)	2.070 (1.771 to 2.369)	-5.297 (-8.507 to -1.975)
Ukraine	0.921 (0.807 to 1.043)	-4.674 (-4.958 to -4.389)	-7.119 (-10.222 to -3.909)
United Arab Emirates	1.310 (1.086 to 1.584)	0.138 (-0.065 to 0.341)	-4.536 (-6.346 to -2.691)
United Kingdom	3.597 (3.144 to 4.046)	0.622 (0.470 to 0.774)	-5.708 (-9.914 to -1.305)
United Republic of Tanzania	0.540 (0.454 to 0.643)	2.496 (2.241 to 2.751)	-1.359 (-2.318 to -0.390)
United States Virgin Islands	0.963 (0.760 to 1.170)	1.523 (1.401 to 1.646)	-4.399 (-6.983 to -1.744)
United States of America	3.678 (3.257 to 4.051)	1.130 (0.928 to 1.333)	-4.608 (-9.502 to 0.551)
Uruguay	2.937 (2.603 to 3.274)	1.095 (0.936 to 1.254)	-4.527 (-7.806 to -1.132)
Uzbekistan	0.578 (0.506 to 0.658)	-2.123 (-2.741 to -1.501)	-1.320 (-5.307 to 2.836)
Vanuatu	2.479 (1.603 to 3.199)	0.348 (0.173 to 0.524)	-2.465 (-5.827 to 1.018)
Venezuela (Bolivarian Republic of)	1.229 (1.047 to 1.375)	1.963 (1.613 to 2.313)	-4.683 (-8.337 to -0.884)
Viet Nam	2.770 (2.306 to 3.291)	1.505 (1.310 to 1.701)	-0.941 (-2.937 to 1.096)
Yemen	0.754 (0.605 to 0.888)	2.470 (2.300 to 2.640)	2.223 (-0.182 to 4.686)
Zambia	0.499 (0.430 to 0.577)	3.010 (2.584 to 3.437)	-5.803 (-9.450 to -2.010)
Zimbabwe	0.487 (0.406 to 0.566)	1.445 (0.929 to 1.964)	-7.687 (-12.386 to -2.735)

EAPC: Estimated Annual Percentage Change; COPD: Chronic Obstructive Pulmonary Disease; EAPCs for ASRs from 1990 to 2019 (%, 95% UI) were calculated using GBD 2021.
